# ﻿Checklist of Rust Fungi (Basidiomycota, Pucciniales) and their hosts in Indiana, United States of America

**DOI:** 10.3897/mycokeys.121.148853

**Published:** 2025-08-29

**Authors:** Terry J. Torres-Cruz, Mehrdad Abbasi, D. Rabern Simmons, M. Catherine Aime

**Affiliations:** 1 Department of Botany and Plant Pathology, Purdue University, West Lafayette, Indiana, USA Purdue University West Lafayette United States of America; 2 Faculty of Land and Food Systems, The University of British Columbia, Vancouver, British Columbia, Canada The University of British Columbia Vancouver Canada

**Keywords:** Arthur Fungarium, plant pathogen, Tippecanoe County, uredinology

## Abstract

Rust fungi (Pucciniales) comprise a large group of ecologically and economically important plant pathogens distributed globally where their hosts grow. The first published study of rusts in Indiana was carried out 131 years ago, and a revised checklist of all Pucciniales for Indiana has not been compiled since the works of Jackson from 1917 to 1920. Efforts to compile a checklist five years ago revealed a dire need for revision due to taxonomic and nomenclatural changes. We examined historical records, including online databases and literature, as well as new and historical collections in the Arthur Fungarium (PUR) at Purdue University. We provide an annotated checklist of all known species of rust fungi occurring in 90/92 Indiana counties. Names in prior records have been updated to reflect the current classifications for this group, with cross-reference to older names. A total of 301 Pucciniales taxa across 32 genera and their host species have been verified. This work serves as a resource for statewide plant disease diagnosticians and records the extant rust fungal biodiversity in Indiana to guide research efforts, resource management, and conservation.

## ﻿Introduction

Rust fungi (Pucciniales, formerly Uredinales) represent the largest natural group of phytopathogens and can cause devastating diseases in agricultural, horticultural, and forest habitats, resulting in millions of dollars in damage annually (e.g., rusts of wheat, corn, coffee, and soybeans). There are also some species that have been used as biocontrol against weeds, such as *Phragmidium violaceum* (Vargas-Gaete et al. 2019) and *Puccinia* species (Tanner et al. 2015; Bean et al. 2024). They are obligate biotrophs of plants (Arthur 1934; Savile 1971), requiring a living host for development, and have complex life cycles that may require up to seven spore types produced in five different spore stages and alternation between two phylogenetically unrelated host plants to complete their life cycles (Aime et al. 2018). Rusts are distributed across all geographical areas on wild and cultivated plants. It is expected that infectious plant disease ranges will shift under climate change (Caubel et al. 2017; Dudney et al. 2021; Miller et al. 2022), and current lists of species diversity will allow the study of changes in their distribution. Our work seeks to document the current knowledge of rust species present in Indiana.

Rust collection in Indiana began almost 200 years ago. References date back to a specimen labelled *Aecidium dircatatum* (now known as *A. hydnoideum*) on *Dirca palustris* (Thymelaeaceae), collected by Lewis David von Schweinitz during a visit to Hope in 1831 (McCain and Hennen 1982), and records of a rust destroying the foliage of blackberries in Henry and Wayne counties in 1872–1874 (Furnas 1874). However, the first explicit report of a rust fungus in the state was made by John M. Coulter in 1876, who documented a rust on *Lespedeza violacea* (Fabaceae), presumably near Hanover (Jackson 1915; McCain and Hennen 1982). We must also acknowledge the work and legacy of Joseph Charles Arthur that made uredinology flourish in the state of Indiana. Arthur named 29 genera and 309 species of rust fungi from North America and influenced the work of uredinologists like Frank D. Kern and his successors as directors of the Arthur Fungarium (PUR), George B. Cummins and Joe F. Hennen (McCain and Hennen 1982). Indiana became a state strong in uredinology history, heavily influenced by the PUR, housed at Purdue University. This fungarium represents the major center in the United States for taxonomic studies of rust fungi (McCain and Hennen 1982; Kaishian et al. 2024).

**Figure 1.**
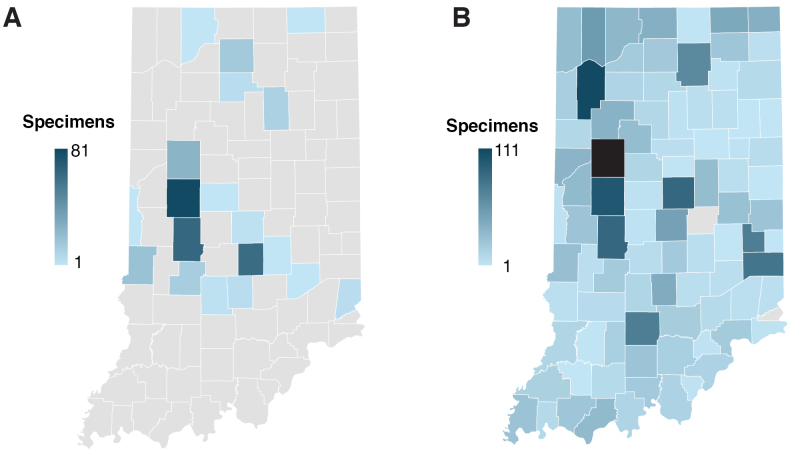
Distribution of specimens evaluated by Indiana counties **A** specimens studied by Underwood (1893) per Indiana county (n = 320) **B** specimens evaluated in this study accessioned in the Arthur Fungarium (PUR) collection (n = 4516; See Suppl. material 1 for detailed metadata for each specimen). Scale bars represent number of specimens studied per Indiana county. Grey color represents no rust reported for that county. Tippecanoe County (filled in black in panel B) was not included in the scale bar representation in panel B for comparison purposes between the two studies, as it was disproportionately represented with 2605 specimens. Individual maps generated with Map charts in Microsoft Excel version 16.88 (Microsoft Corporation, 2024).

The first account of the rusts of Indiana was presented at the annual meeting of the Indiana Academy of Science in 1890 by E. M. Fisher on the Parasitic Fungi of Indiana, with a considerable number of species mentioned; however, this work was never published (Jackson 1915). A list of those species was obtained by Dr. L. M. Underwood and was included in the first published compilation of Indiana rust fungi by Underwood (1893). It listed 88 rust species observed across 19 counties, with most observations reported from Montgomery, Putnam, Johnson, and Tippecanoe counties (Fig. 1A). Additional lists were later published, including a list of rusts of Tippecanoe County in 1896 by Lillian Snyder and supplemented in 1898 with lists from Madison and Noble counties, as well as lists for Hamilton and Marion counties by G. W. Wilson in 1905, and two complete state lists by J. C. Arthur (Jackson 1915). An updated list was published by Jackson (1915), reporting 141 rusts, 34 of which were new reports for Indiana. The number of rusts for Indiana was later updated to 155 (Jackson 1917) and 165 (Jackson 1920). Since the efforts by Jackson, various rusts and new host records have been reported as part of the sixth annual A. H. Smith Great Lakes Foray (Schoknecht 1981), as specimens evaluated in rust biogeography studies (McCain and Hennen 1981), and studies of rusts on Cyperaceae and Juncaceae in Indiana (McCain 1984). In recent years, efforts have been carried out to document all fungi in the state, including a checklist of Indiana microfungi (Bates et al. 2019). However, the taxonomy for Pucciniales in this list needs considerable revision. Despite the significant efforts to document rusts in Indiana from the past 100 years, a checklist of all Pucciniales in the state has not been compiled since the works of Jackson from 1917 to 1920. This study was undertaken to generate an updated list with a modern re-evaluation.

Checklists are vital sources of information that set a benchmark for decision-making in research efforts, resource management, and policy (Reyserhove et al. 2020). Checklists have been valuable in monitoring, modeling, and predicting species presence and distribution in diverse groups of organisms. These include pollinators (IPBES 2016), rare species (IUCN 2020), and invasive alien species (Seebens et al. 2017; Reyserhove et al. 2020). They are instrumental in studies related to biodiversity conservation initiatives (Lowman et al. 2018; Parker et al. 2018; Menchetti et al. 2021; Lacher et al. 2022), discovery of new species (Pierson et al. 2014; Cantonwine et al. 2019; Nicolai et al. 2020; Maharani et al. 2022), and changes in response to climate change (Pearman et al. 2024). Furthermore, the use of herbarium records is highly valuable in ecological and epidemiological studies and modeling, such as assisting with the listing of rare taxa (Molano-Flores et al. 2023), providing data for biodiversity and landscape monitoring (Çelekli and Zariç 2023, informing conservation biology (Greve et al. 2016), setting baseline data particularly for poorly inventoried regions (Greve et al. 2016; Park et al. 2022), and supporting the study of phenological (Jones and Daehler 2018; Williams et al. 2021) and global environmental changes (Heberling et al. 2019; Lang et al. 2019). Access to the Arthur Fungarium, in this study, presented a valuable opportunity to assess and document the presence of rusts across Indiana with the purpose of generating a curated dataset to inform future ecological, epidemiological, and conservation work. This project verified records for potential misidentifications, known synonymies, and use of incorrect or obsolete names to reflect the most current classification for these rusts and the report of new records for the state. We provide a complete, up-to-date record of the Pucciniales species and their hosts known from Indiana.

## ﻿Methods

This checklist was compiled from a variety of sources, including published literature (e.g., Arthur 1934), online databases [MyCoPortal: https://mycoportal.org/portal/collections/list.php (MyCoPortal 2025 and the U.S. National Fungus Collections database: https://nt.ars-grin.gov/fungaldatabases/ (Farr and Rossman 2018) and unpublished herbarium records. Data for the Arthur Fungarium (PUR) are maintained in the open-source software “Specify 7” by the Specify Collections Consortium (https://specifysoftware.org). Sources for each listing are provided within the list. The authors of fungal names follow the conventions in Index Fungorum (http://www.indexfungorum.org/names/AuthorsOfFungalNames.asp), and the current name of host plants follows the conventions used by the PLANTS database (http://plants.usda.gov) and Plants of the World Online (http://www.plantsoftheworldonline.org/). Herbarium specimens were studied to confirm dubious names or records, as well as for species represented by only a single statewide report.

The checklist is arranged alphabetically by genus, species, and subspecies-level taxa. Each species/subspecies is annotated with host family, species, and source of record. The current names of rust species are printed in bold type. Names not printed in bold are synonyms or other non-current names. References were inserted following the host name. The checklist does not include species that were the result of inoculation studies in greenhouses and that are not naturally occurring in the area (i.e., *Hemileia vastatrix*—PUR66308 and *Phakopsora zeae*—PUR51735).

We generated a specimen number by county list using location information from verified vouchers in the Arthur Fungarium (Suppl. material 1). Other sources of observations were not included in this analysis, since the level of detail for location varied among specimens. Metadata for records originating from PUR accessions are available in Suppl. material 2.

Maps depicting the number of observations per Indiana county were generated using map charts in Microsoft Excel version 16.88 (Microsoft Corporation, 2024). Additionally, all taxon names on the checklist were cross-checked against MycoBank and Index Fungorum by running a search for each name. Search results accuracy was categorized based on whether the correct current name was present in the search results as the current name or listed as a synonym, if an older name or another name was referenced as the current name in the database, or if a search of the current name or old name returned no results. The results from these searches are available in Suppl. material 3.

## ﻿Results and discussion

Our checklist of rust fungi in Indiana includes a total of 301 current names, 339 synonyms and non-current names, and 13 ambiguous names. It documents 301 Pucciniales taxa (275 species, 21 varieties, 4 subspecies, and 1 forma specialis) distributed on 900+ host taxa. Most of the specimens from Indiana housed in the Arthur Fungarium are from Tippecanoe County (57.6%), with 2,605 out of 4,516 specimens collected in the same county where the fungarium is located. The next most sampled counties show a markedly smaller percentage, with Jasper (2.45%; 111 specimens), Montgomery (2.24%; 101 specimens), Hamilton (1.97%; 89 specimens), Putnam (1.97%; 89 specimens), Franklin (1.77%; 80 specimens), Fayette (1.64%; 74 specimens), Lawrence (1.64%; 74 specimens), and Kosciusko (1.44%; 65 specimens) counties. A total of 28% of counties (19/90) have between 20 and 53 specimens vouchered in the PUR (from highest specimen number to lowest: Porter, Marion, LaGrange, Brown, St. Joseph, Steuben, White, Warren, LaPorte, Fountain, Spencer, Lake, Carroll, Madison, Vigo, Henry, Posey, Orange, Parke, Wayne, Noble, Warrick, Marshall, Crawford, and Johnson). While another 28% of counties (19/90) have between 10 and 19 specimens accessioned per county (from highest specimen number to lowest: Jefferson, Owen, Starke, Jackson, Newton, Martin, Washington, Gibson, Clark, Harrison, Perry, Pulaski, Clinton, Knox, Monroe, Benton, Cass, Fulton, and Vanderburgh), 41% of the Indiana counties represented in the PUR collection have less than 10 specimens vouchered in the PUR per county (Fig. 1B). A detailed list of all counties and their respective number of specimens in the PUR is provided in Suppl. material 2.

The recorded distribution of rust fungi in Indiana has been attributed to the distribution of the collectors and is likely representative of changes in land use (e.g., conversion to agricultural land in a large part of the state). McCain and Hennen (1981) pointed out that over 70% of Indiana rust fungi at the time occurred within easy collecting distance of West Lafayette, where the PUR is located (e.g., Fig. 1A). The distribution data was skewed away from eastern and southern Indiana, with information at the time only for 44 of the 92 Indiana counties, evidently exemplifying the distribution of collectors rather than of taxa (McCain 1984). Although this disproportionate representation from Tippecanoe County persists—an issue that continues to this day—the PUR currently houses specimens for 90/92 counties in Indiana, only lacking representatives from Hancock and Ohio counties. This highlights the advances in Pucciniales knowledge for Indiana in the past 40+ years and the efforts to conduct fieldwork across the state. Yet, it is still clear that systematic statewide specimen collection is lacking to generate valid biogeographical or phenological hypotheses (McCain 1984).

Our checklist includes a total of 339 Pucciniales taxon names that had been previously reported for Indiana that are currently obsolete names, as well as 13 ambiguous taxon names. Taxonomic confusion has characterized rust fungi due to the development of multiple classification schemes, homoplasy in diagnostic characters, and the use of separate naming systems for sexual and asexual morphs under prior nomenclatural codes. Additionally, more than 200 generic names have been described in Pucciniales, but only about one-third of those names are accepted (Aime and McTaggart 2021). Taxonomy and nomenclature of many of the rusts reported in Indiana have changed over time and not always kept up to date in records. This became evident in the rusts listed in the recent checklist of Indiana microfungi (Bates et al. 2019). This study provides updated names with the most correct identification based on a re-evaluation of the dataset considering current taxonomy.

**Figure 2.**
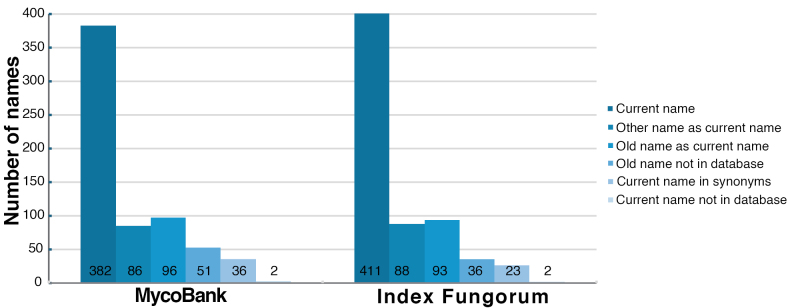
MycoBank and Index Fungorum taxonomic accuracy in comparison to the curated list of taxa in our Indiana rust fungi checklist (n = 653) after conducting a search for each taxon name in our list in both repositories. Search results accuracy was categorized based on whether the correct current name was present in the search results as the current name or listed as a synonym, if an older name or another name was referenced as the current name in the database, or if a search of the current name or old name returned no results.

Index Fungorum (https://www.indexfungorum.org/) and MycoBank (https://www.mycobank.org/) are regularly consulted by mycologists and plant pathologists to assign current taxonomy to their rust fungi samples. Nevertheless, we note that these public name repositories may still contain inaccurate names (Suppl. material 3). Both repositories seem to follow similar patterns of accuracy/inaccuracy (Fig. 2). Of the 653 names listed on our checklist (301 current names, 339 synonyms and non-current names, and 13 ambiguous names), only 58.5% (MycoBank) and 63% (Index Fungorum) of these names directly refer to the correct name when conducting a search in each database. The misidentifications and use of obsolete names are usually not congruent between the two databases. Discrepancy issues between these repositories are known and extend to disagreements in terms of placing genera to family- and order-level, based on differential updates and taxonomic opinion (Durkin et al. 2020). Our goal is that this resource, with the most up-to-date taxonomy, serves as a reference point for correct identification of rusts from Indiana to plant diagnosticians and researchers.

### ﻿Indiana checklist of rust fungi (Pucciniales)

In this checklist, 301 rust taxa from Indiana are reported. A list of host species, taxonomic/nomenclatural synonyms of each rust taxon, and references are also included.

*Aecidium albiperidium* Arthur

See: *Puccinia caricina* DC., s.lat.

*Aecidium aquilegiae* Pers.

See: *Puccinia persistens* Plowr. s.lat.

*Aecidium asperifolii* Pers.

See: *Puccinia recondita* Roberge ex Desm.

*Aecidium asterum* Schwein.

See: *Puccinia dioicae* Magnus, s.lat.

*Aecidium berberidis* Pers.

See: *Puccinia graminis* Pers.

***Aecidium boehmeriae* Arthur**

*Boehmeria cylindrica* (L.) Sw. (Urticaceae) (Jackson 1915; Anderson and Anderson 1919; USDA 1960) PUR: 42992

***Aecidium compositarum* Mart.**

*Eupatorium perfoliatum* L. (Asteraceae) (Snyder 1896)

Note: Probably aecial state of *Puccinia eleocharidis* Arthur.

***Aecidium cyparissiae* DC.**

*Euphorbia commutata* Engelm. ex A. Gray (Euphorbiaceae) (Underwood 1896)

Note: Probably refers to *Aecidium tithymali* Arthur.

***Aecidium dakotense* Griffiths**

*Thalictrum dioicum* L. (Ranunculaceae) (Jackson 1920) PUR: 6760

*Aecidium dicentrae* Trel.

See: *Aplopsora dicentrae* (Mains & H.W. Anderson) Buriticá & J.F. Hennen

*Aecidium dracontii* Schwein. [as ‘*dracontinatum*’ in protologue]

See: *Uromyces ari-triphylli* (Schwein.) Seeler

*Aecidium erigeronatum* Schwein.

See: *Puccinia dioicae* Magnus, s.lat.

*Aecidium eupatorii* Dietel

See: *Puccinia eleocharidis* Arthur

*Aecidium euphorbiae* Pers. ex J.F. Gmel.

See: *Uromyces proëminens* (DC.) Lév.

*Aecidium geranii* DC.

See: *Uromyces geranii* (DC.) Lév.

*Aecidium grossulariae* (J.F. Gmel.) DC.

See: *Puccinia caricina* DC., s.lat.

*Aecidium hepaticatum* Schwein.

See: *Tranzschelia arthurii* Tranzschel & M.A. Litv.

*Aecidium hydnoideum* Berk. & M.A. Curtis

See: *Puccinia dioicae* Magnus, s.lat.

*Aecidium impatientis* Schwein.

See: *Puccinia impatientis* (Schwein.) Arthur

*Aecidium lycopi* W.R. Gerard

See: *Puccinia angustata* Peck

*Aecidium mariae-wilsoniae* Peck [as ‘*mariae-wilsoni*’]

See: *Puccinia ellisiana* Thüm.

*Aecidium oenotherae* Peck

See: *Puccinia dioicae* Magnus, s.lat.

*Aecidium onobrychidis* Burrill

See: *Puccinia andropogonis* Schwein.

*Aecidium pedatatum* Schwein

See: *Uromyces andropogonis* Tracy

*Aecidium pentastemonis* Schwein.

See: *Puccinia andropogonis* Schwein.

*Aecidium physalidis* Burrill

See: *Puccinia kansensis* Ellis & Barthol.

*Aecidium pteleae* Berk. & M.A. Curtis

See: *Puccinia windsoriae* Schwein.

*Aecidium pustulatum* M.A. Curtis

See: *Puccinia andropogonis* Schwein.

*Aecidium ranunculacearum* DC.

*Anemone pensylvanica* L. (Ranunculaceae) (Snyder 1896)

Notes: *A. ranunculacearum* is an ambiguous name and may refer to *Puccinia magnusiana*, *P. recondita*, or *Uromyces dactylidis*.

*Aecidium ranunculi* Schwein.

See: *Puccinia eatoniae* Arthur

*Aecidium sambuci* Schwein.

See: *Puccinia sambuci* (Schwein.) Arthur

***Aecidium tithymali* Arthur**

*Euphorbia commutata* Engelm. ex A. Gray (=*Tithymalus commutatus* (Engelm. ex A. Gray) Klotzsch & Garcke (Euphorbiaceae) (Jackson 1917; Arthur 1918; Anderson and Anderson 1919; USDA 1960) PUR: 43105, 43110, 43111, 43112, 43113, 43114, 47301, N6674.

*Aecidium trillii* Burrill

See: *Uromyces halstedii* De Toni

*Aecidium verbenicola* Ellis & Kellerm. (Also reported as *Aecidium verbenae* Speg. [author as ‘Spreng.’]

See: *Puccinia vilfae* Arthur & Holw.

*Allodus ambigua* (Alb. & Schwein.) Arthur

See: *Puccinia difformis* Kunze

*Allodus claytoniata* (Schwein.) Arthur

See: *Puccinia mariae-wilsoniae* G.W. Clinton

***Allodus podophylli* (Schwein.) Arthur**

*Podophyllum peltatum* L. (Berberidaceae) (Underwood 1893; Snyder 1896, 1898; Arthur 1899; Jackson 1915; Anderson and Anderson 1919) PUR: 35717, 35720, 35721, 35727, 35731, 35732, 35733, 35734, 35735, 35736, 35737, 35741, 35742, 35743, 35744, 35750, 35751, 35753, 35754, 35756, 35764, 35767, 35768, 35770, 35771, 35773, 35777, 35780, 35781, 35794, 35795, 35796, 35797, 35803, 35804, 35805, 35807, 35812, 35813, 35814, 35816, 35818, 46794, 46795, 46810, 65820, 88839, 89279, N617, N2151, N2152, N2153, N4516, N4530, N4668, N5872, N5873, N5874, N5875, N5876, N5877, N5878

Host not reported (Van Hook 1910)

*Allodus tenuis* (Schwein.) Arthur

See: *Puccinia tenuis* Burrill

***Aplopsora dicentrae* (Mains & H.W. Anderson) Buriticá & J.F. Hennen**

*Dicentra cucullaria* (L.) Bernh. (Fumariaceae) (Underwood 1893; Anderson and Anderson 1919; Buriticá 1998); (=*Bicuculla cucullaria* (L.) Millsp.) (Jackson 1917)

*Laportea canadensis* (L.) Weddell (Urticaceae) (Buriticá 1998)

***Aplopsora tanakae* (S. Ito) Buriticá & J.F. Hennen**

*Amphicarpaea bracteata* (L.) Fernald (Fabaceae) (Emmons et al. 1960; Cummins 1962; Cooke 1975; Buriticá 1998)

*Aregma disciflora* (Tode) Arthur

See: *Phragmidium mucronatum* (Pers.) Schltdl.

*Aregma fragariae* (DC.) Arthur

See: *Phragmidium tormentillae* Fuckel

*Aregma speciosum* Fr. [as ‘speciosa’]

See: *Phragmidium speciosum* (Fr.) Cooke

***Bubakia crotonis* (Cooke) Arthur**

*Croton monanthogynus* Michx. (Euphorbiaceae) (Jackson 1915)

*Croton* sp. PUR: N2586

*Bullaria bardanae* (Corda) Arthur

See: *Puccinia bardanae* (Wallr.) Corda

*Bullaria bullata* (Pers.) Arthur

See: *Puccinia angelicae* (Schumach.) Fuckel

*Bullaria chrysanthemi* (Roze) Arthur

See: Puccinia tanaceti var. tanaceti DC.

*Bullaria globosipes* (Peck) H.S. Jacks.

See: *Puccinia globosipes* Peck

*Bullaria hieracii* (Schumach.) Arthur

See: *Puccinia hieracii* (Röhl.) H. Mart.

*Bullaria kuhniae* (Schwein.) Kern

See: *Puccinia kuhniae* Schwein.

*Bullaria kuhniae* (Schwein.) H.S. Jacks. (nomen invalidum)

See: *Puccinia kuhniae* Schwein.

*Bullaria taraxaci* Arthur

See: *Puccinia hieracii* (Röhl.) H. Mart.

*Caeoma agrimoniae* Schwein.

See: *Pucciniastrum agrimoniae* (Dietel) Tranzschel

*Caeoma nitens* Schwein.

See: *Gymnoconia nitens* (Schwein.) Arthur

*Caeomurus caladii* (Schwein.) Kuntze

See: *Uromyces ari-triphylli* (Schwein.) Seeler

*Caeomurus caryophyllinus* (Schrank) Kuntze

See: *Uromyces dianthi* (Pers.) Niessl

*Caeomurus euphorbiae* (Cooke & Peck) Kuntze

See: *Uromyces proëminens* (DC.) Lév.

*Caeomurus gaurinus* (Peck) Arthur

See: *Uromyces plumbarius* Peck

*Caeomurus graminicola* (Burrill) Kuntze [as ‘*graminicolus*’]

See: *Puccinia graminicola* (Burrill) Demers & Castl.

*Caeomurus howei* (Peck) Kuntze

See: *Uromyces asclepiadis* Cooke

*Caeomurus hedysari-paniculati* (Schwein.) Arthur

See: *Uromyces hedysari-paniculati* (Schwein.) Farl.

*Caeomurus hyperici-frondosi* (Schwein.) Arthur

See: *Uromyces triquetrus* Cooke

*Caeomurus junci* (Tul. & C. Tul.) Kuntze

See: *Uromyces junci* (Desm.) Tul. & C. Tul.

*Caeomurus lespedezae-procumbentis* (Schwein.) Arthur

See: *Uromyces lespedezae-procumbentis* (Schwein.) M.A. Curtis

*Caeomurus peryginus* (Halst.) Kuntze [as ‘*perigynius*’]

See: *Uromyces perigynius* Halst.

*Caeomurus phaseoli* (Pers.) Arthur

See: Uromyces appendiculatus var. appendiculatus (Pers.) Unger

*Caeomurus pisi* (Pers.) Gray

This is an illegitimate name (Art. 53.1)

See: *Uromyces viciae-fabae* (Pers.) J. Schröt.

*Caeomurus polygoni* (Pers.) Kuntze

See: *Uromyces polygoni-avicularis* (Pers.) G.H. Otth

*Caeomurus rudbeckiae* (Arthur & Holw.) Kuntze

See: *Uromyces rudbeckiae* Arthur & Holw

*Caeomurus terebinthi* (DC.) Kuntze

See: *Pileolaria brevipes* Berk. & Ravenel

*Caeomurus trifolii* (R. Hedw.) Gray, nom. ambig.

See: *Uromyces trifolii*; U. trifolii-repentis var. trifolii-repentis and U. trifolii-repentis var. fallens

*Trifolium medium* L. (Fabaceae) (Arthur 1899) [Doubtful record]

***Calyptospora columnaris* (Alb. & Schwein.) J.G. Kühn**

*Abies balsamea* (L.) Mill. (Pinaceae)

*Cerotelium tanakae* S. Ito

See: *Aplopsora tanakae* (S. Ito) Buriticá & J.F. Hennen

*Chrysomyxa albida* J.G. Kühn

See: *Kuehneola uredinis* (Link) Arthur

***Chrysomyxa ledicola* (Peck) Lagerh.**

*Picea mariana* (Mill.) Britton, Sterns & Poggenb. (Pinaceae) PUR: 4729

***Coleosporium apocynaceum* Cooke**

*Amsonia* cv. ‘Blue Ice’ (Apocynaceae) (Abbasi et al. 2016a)

***Coleosporium asterum* (Dietel) Syd. & P. Syd.**

*Aster salicifolius* Lam. (Asteraceae) (Jackson 1915)

*A. shortii* Hook (Jackson 1915)

*Callistephus chinensis* (L.) Nees (=*Callistephus hortensis* Cass.) (Asteraceae)

*C. hortensis* Cass. (Jackson 1915; Pipal 1915; Anderson and Anderson 1919)

*Pinus echinata* Mill. (Pinaceae)

*Pinus* sp.

*P. taeda* L.

*Solidago altissima* L. (Asteraceae) (Anderson and Anderson 1919; Emmons et al. 1960; Schoknecht 1981)

*S. arguta* Aiton (Underwood 1893; Arthur 1899; Jackson 1915; Anderson and Anderson 1919)

*S. bicolor* L. (Anderson and Anderson 1919; Jackson 1920)

*S. caesia* L. (Underwood 1893; Arthur 1899; Jackson 1915; Anderson and Anderson 1919; Emmons et al. 1960; Schoknecht 1981)

*S. canadensis* (cf.) L. (Arthur 1899; Anderson and Anderson 1919)

*S. canadensis* L. (Underwood 1893; Jackson 1915)

*S. erecta* Pursh (Jackson 1920)

*S. flexicaulis* L. (Arthur 1899; Jackson 1915); (=*S. latifolia* L.) (Underwood 1893; Anderson and Anderson 1919)

*S. gigantea* Aiton (=S. gigantea subsp. serotina (Kuntze) McNeill) (McCain et al. 1990); (=*S. serotina* Aiton) (Underwood 1893; Arthur 1899; Jackson 1915)

*S. juncea* Aiton (Jackson 1920; Cooke 1967)

*S. nemoralis* Aiton (McCain et al. 1990)

*S. patula* Muhl. ex Willd. (Underwood 1893; Arthur 1899; Jackson 1915; Anderson and Anderson 1919)

*S. rugosa* Mill. (Underwood 1893; Arthur 1899; Jackson 1915)

*Solidago* sp. (Snyder 1896)

*S. uliginosa* Nutt.

*S. ulmifolia* Muhl. ex Willd. (Jackson 1915; Anderson and Anderson 1919; Emmons et al. 1960)

*Symphyotrichum cordifolium* (L.) G.L. Nesom (=*Aster cordifolius* L. and *A. sagittifolius* Wedemeyer ex Willd.) (Asteraceae) (Underwood 1893; Arthur 1899; Jackson 1915; Anderson and Anderson 1919)

*S. drummondii* (Lindl.) G.L. Nesom; var. *drummondii (Lindl.)* G.L. Nesom (=*A. drummondii* Lindl.) (Jackson 1915)

S. ericoides (L.) G.L. Nesom; var. ericoides (=*A. ericoides* L.) (Jackson 1915; Anderson and Anderson 1919)

*S. laeve* (L.) A. Löve & D. Löve

S. lanceolatum (Willd.) G.L. Nesom; ssp. lanceolatum var. lanceolatum (=*A. paniculatus* Lam.) (Underwood 1893; Arthur 1899; Jackson 1915; Anderson and Anderson 1919)

*S. lateriflorum* (L.) A. Löve & D. Löve

*S. novae-angliae* (L.) G.L. Nesom; (=*A. novae-angliae* L.) (Underwood 1893; Arthur 1899; Jackson 1915; Anderson and Anderson 1919)

S. novi-belgii (L.) G.L. Nesom var. novi-belgii (=*A. longifolius* Lam.) (Jackson 1915)

S. oolentangiense (Riddell) G.L. Nesom var. oolentangiense (=*A. azureus* Lindl.) (Underwood 1893; Arthur 1899; Jackson 1915; Anderson and Anderson 1919)

S. pilosum var. pilosum (Willd.) G.L. Nesom (=*A. pilosus* Willd.)

*S. praealtum* (Poir.) G.L. Nesom (=*A. salicifolius* Aiton) (Underwood 1893); var. praealtum (Poir.) G.L. Nesom (=*A. praealtus* Poir.) (McCain et al. 1990)

S. puniceum (L.) Á. Löve & D. Löve; var. puniceum (L.) Á. Löve & D. Löve (=*A. firmus* Nees) (McCain et al. 1990); (=*A. puniceus* L.) (Underwood 1893; Arthur 1899; Jackson 1915); (=*S. firmum* (Nees) G.L. Nesom)

*S. shortii* (Lindl.) G.L. Nesom (=*A. shortii* Lindl.) (Underwood 1893; Anderson and Anderson 1919)

*Symphyotrichum* sp. (=*Aster* sp.) (Snyder 1898)

*S. tradescantii* (L.) G.L. Nesom (=*A. tradescantii* L.) (Underwood 1893; Arthur 1899; Jackson 1915)

***Coleosporium campanulae* (Pers.) Tul.**

*Campanula rapunculoides* L. (Campanulaceae) (McCain and Hennen 1981; McCain et al. 1990)

*Campanulastrum americanum* (L.) Small (=*Campanula americana* L.) (Campanulaceae) (Jackson 1915; Anderson and Anderson 1919; Cooke 1967)

***Coleosporium delicatulum* (Arthur & F. Kern) Hedgc. & Long**

*Euthamia graminifolia* (L.) Nutt. (Asteraceae) (Jackson 1915); var. graminifolia (L.) Nutt. (=*Solidago graminifolia* (L.) Salisb.) (Emmons et al. 1960)

*Solidago lanceolata* L. (Asteraceae) (Anderson and Anderson 1919)

***Coleosporium elephantopi* Thüm. [as ‘ *elephantopodis*** ’]

*Elephantopus carolinianus* Willd. (Asteraceae) (Jackson 1915)

***Coleosporium helianthi* (Schweinitz) Arthur**

*Helianthus decapetalus* L. (Asteraceae) (Jackson 1915)

*H. microcephalus* Torr. & A. Gray (McCain et al. 1990)

*H. giganteus* L. (USDA 1960)

*H. hirsutus* Raf. (Jackson 1915)

*Silphium integrifolium* Michx. (Asteraceae) (Jackson 1915)

*S. perfoliatum* L.

*S. terebinthinaceum* Jacq. (Jackson 1915)

*Coleosporium hydrangeae* (Berk. & M.A. Curtis) L. Snyder

See: *Pucciniastrum hydrangeae* (Magnus) Arthur

***Coleosporium ipomoeae* (Schwein.) Burrill**

*Ipomoea pandurata* (L.) G. Mey. (Convolvulaceae) (Underwood 1896; Arthur 1899; Jackson 1915; Anderson and Anderson 1919)

*Ipomoea* sp. (Snyder 1896)

***Coleosporium montanum* (Arthur & F. Kern) McTaggart & Aime**

*Solidago* sp. (Asteraceae) (McTaggart and Aime 2018) PUR: N11689, N21953

***Coleosporium pinicola* (Arthur) Arthur**

*Pinus virginiana* Mill. (Pinaceae) (USDA 1960)

*Coleosporium rubi* Ellis & Holw.

See: *Kuehneola uredinis* (Link) Arthur

***Coleosporium solidaginis* (Schwein.) Thüm.**

*Solidago canadensis* (Asteraceae) PUR: N23076, N23056

*Coleosporium sonchi-arvensis* (Pers.) Lév.

*Hieracium* sp. (Asteraceae) (Underwood 1893) [Doubtful record]

See: *Coleosporium asterum* (Dietel) Syd. & P. Syd. and *C. vernoniae* Berk. & M.A. Curtis

*Coleosporium terebinthinaceae* (Schwein.) Arthur

See: *Coleosporium helianthi* (Schweinitz) Arthur

***Coleosporium tussilaginis* (Pers.) Lév.**

*Packera aurea* (L.) Á. Löve & D. Löve (=*Senecio aureus* L.) (Asteraceae) (McCain et al. 1990)

*Senecio* sp. (Asteraceae) PUR: 89361

***Coleosporium vernoniae* Berk. & M.A. Curtis**

*Elephantopus carolinianus* Raeusch. (Asteraceae)

*Pinus echinata* Mill. (Pinaceae)

*P. nigra* Arnold (USDA 1960)

*P. rigida* Mill.

*P. virginiana* Mill.

*Vernonia arkansana* DC. (Asteraceae)

*V. fasciculata* Michx. (Underwood 1893; Arthur 1899; Jackson 1915; Anderson and Anderson 1919)

V. gigantea (Walter) Trel. ; subsp. gigantea (=*V. altissima* Nutt.) (Jackson 1915; Anderson and Anderson 1919; Emmons et al. 1960; Schoknecht 1981)

*Vernonia × illinoensis* Gleason [*gigantea × missurica*] (Jackson 1920)

*V. missurica* Raf.

*V. noveboracensis* (L.) Michx. (Underwood 1893; Arthur 1899; Jackson 1915; Van Hook 1927)

*Vernonia* sp. (Cooke 1967)

***Coleosporium viburni* Arthur**

*Viburnum lentago* L. (Caprifoliaceae)

*Viburnum* sp. (Ruhl et al. 1982)

***Coleosporium* sp.**

*Pinus* sp. (Pinaceae) (Ruhl et al. 1983)

***Cronartium comandrae* Peck**

*Comandra umbellata* (L.) Nutt. (Santalaceae)

***Cronartium fusiforme* Hedgc. & N.R. Hunt ex Cummins**

*Pinus* sp. (Pinaceae) (Ruhl et al. 1982)

***Cronartium harknessii* E. Meinecke**

*Pinus* sp. (Pinaceae) (Ruhl et al. 1983)

***Cronartium quercuum* (Berk.) Miyabe ex Shirai**

*Quercus alba* L. (Fagaceae)

*Q. bicolor* Willd.

*Q. macrocarpa* Michx.

*Q. michauxii* Nutt.

*Q. muehlenbergii* Engelm.

*Q. pagoda* Raf.

*Q. phellos* L.

*Q. rubra* L.

***Cronartium ribicola* J.C. Fisch.**

*Ribes cynosbati* L. (Grossulariaceae) (Emmons et al. 1960)

*Ribes* sp.

R. aureum var. villosum DC. (=*R. odoratum* H.L. Wendl.) (Wilson et al. 2014)

*Dasyspora anemones-virginianae* (Schwein.) Arthur

See: *Puccinia anemones-virginianae* Schwein.

*Dasyspora asteris* (Duby) Arthur

See: *Puccinia cnici-oleracei* Pers. ex Desm.

*Dasyspora circaeae* (Pers.) Arthur

See: *Puccinia circaeae* Pers.

*Dasyspora dayi* (Clinton) Arthur

See: *Puccinia dayi* Clinton

*Dasyspora glechomatis* (DC.) Arthur [as *gleocomatis*]

See: *Puccinia glechomatis* DC.

*Dasyspora lobeliae* (W.R. Gerard) Arthur

See: *Puccinia lobeliae* W.R. Gerard

*Dasyspora malvacearum* (Bertero) Arthur

See: *Puccinia malvacearum* Bertero ex Mont.

*Dasyspora physostegiae* (Peck & Clinton) H.S. Jacks.

See: *Puccinia physostegiae* Peck & Clinton

*Dasyspora ranunculi* (Kuntze) Arthur

See: *Puccinia andina* Dietel & Neger

*Dasyspora saxifragae* (Schltdl.) Arthur

See: *Puccinia heucherae* (Schwein.) Dietel

*Dasyspora seymeriae* (Burrill) Arthur [as ‘*seymouriae*’ by Arthur (1906)]

See: *Puccinia seymeriae* Burrill

*Dasyspora silphii* (Schwein.) Arthur

See: *Puccinia silphii* Schwein.

*Dasyspora xanthii* (Schwein.) Arthur

See: *Puccinia xanthii* Schwein.

*Dicaeoma aletridis* (Berk. & M.A. Curtis) Kuntze

See: *Puccinia aletridis* Berk. & M.A. Curtis

*Dicaeoma andropogi* (Schwein.) Kuntze

See: *Puccinia andropogonis* Schwein.

*Dicaeoma andropogonis* (Schwein.) Kuntze

See: *Puccinia andropogonis* Schwein.

*Dicaeoma anemones* (Pers.) Arthur

See: *Tranzschelia pseudofusca* M. Scholler & M. Abbasi

*Dicaeoma anemones-virginianae* (Schwein.) Arthur

See: *Puccinia anemones-virginianae* Schwein.

*Dicaeoma angustatum* (Peck) Kuntze

See: *Puccinia angustata* Peck

*Dicaeoma antirrhini* (Dietel & Holw.) H.S. Jacks.

See: *Puccinia antirrhini* Dietel & Holw.

*Dicaeoma apocryptum* (Ellis & Tracy) Kuntze

See: *Puccinia apocrypta* Ellis & Tracy

*Dicaeoma argentatum* (Schultz) Kuntze

See: *Puccinia argentata* (Schultz) G. Winter

*Dicaeoma asparagi* (DC.) Kuntze

See: *Puccinia asparagi* DC.

*Dicaeoma asperifolii* (Pers.) Kuntze

See: *Puccinia recondita* Roberge ex Desm. s.str.

*Dicaeoma asperifolii* (Pers.) Kuntze, sensu Arthur (1899), nom. ambig.

See: *Puccinia impatientis* (Schwein.) Arthur and *P. recondita* Roberge ex Desm. s.str.

*Avena sativa* L (Poaceae) (Arthur 1899) [Doubtful record]

*Dicaeoma asteris* (Duby) Kuntze

See: *Puccinia cnici-oleracei* Pers. ex Desm.

*Dicaeoma bolleyanum* (Sacc.) Kuntze

See: *Puccinia sambuci* (Schwein.) Arthur

*Dicaeoma calthae* (Link) Kuntze

See: *Puccinia calthae* Link

*Dicaeoma canaliculatum* (Schwein.) Kuntze

See: *Puccinia canaliculata* (Schwein.) Lagerh.

*Dicaeoma cephalanthi* (Seym.) H.S. Jacks.

See: *Puccinia seymouriana* Arthur

*Dicaeoma circaeae* (Pers.) Kuntze

See: *Puccinia circaeae* Pers.

*Dicaeoma cnici* H. (Mart.) Arthur

See: *Puccinia cnici* H. Mart.

*Dicaeoma clematidis* (DC.) Arthur, nom. ambig.

This is an ambiguous name applied by different authors to different taxa.

*Aquilegia* sp. (Ranunculaceae) (Jackson 1915)

*Clematis virginiana* L. (Ranunculaceae) (Jackson 1915)

*Thalictrum thalictroides* (L.) Eames & B. Boivin (=*Syndesmon thalictroides* (L.) Hoffmanns. ex Britton (Ranunculaceae) (Jackson 1915)

*Elymus repens* (L.) Gould (=*Agropyron repens* (L.) P. Beauv.) (Poaceae) (Jackson 1915)

*Bromus ciliatus* L. (Poaceae) (Jackson 1915)

*B. arvensis* L. (=*Bromus japonicus* Thunb.) (Jackson 1915)

B. ciliatus var. purgans (L.) A. Gray (=*B. purgans* L.) (Jackson 1915)

*B. secalinus* L. (Jackson 1915)

*Hordeum jubatum* L. (Poaceae) (Jackson 1915)

Glyceria grandis S. Watson var. grandis (=*Panicularia grandis* (S. Watson) Nash (Poaceae) (Jackson 1915)

*Dicaeoma conoclinii* (Seym.) Kuntze

See: *Puccinia conoclinii* Seym. ex Burrill

*Dicaeoma convolvuli* (Pers.) Kuntze

See: *Puccinia convolvuli* (Pers.) Castagne

*Dicaeoma cyperi* (Arthur) Kuntze

See: *Puccinia cyperi* Arthur

*Dicaeoma cypripedii* (Arthur) Arthur

See: *Puccinia cypripedii* Arthur

*Dicaeoma dayi* (Clinton) Kuntze

See: *Puccinia dayi* Clinton

*Dicaeoma dochmia* (Berk. & M.A. Curtis) Kuntze

See: *Puccinia dochmia* Berk. & M.A. Curtis

*Dicaeoma eatoniae* Arthur

See: *Puccinia eatoniae* Arthur

*Dicaeoma eleocharidis* (Arthur) Kuntze

See: *Puccinia eleocharidis* Arthur

*Dicaeoma ellisianum* (Thüm.) Kuntze

See: *Puccinia ellisiana* Thüm.

*Dicaeoma emaculatum* (Schwein.) Kuntze

See: *Puccinia emaculata* Schwein.

*Dicaeoma epiphyllum* (L.) Kuntze

See: *Puccinia poarum* Nielsen

[Jackson’s (1920) report on *Alopecurus geniculatus* L. is *Puccinia poae-nemoralis* G.H. Otth]

*Dicaeoma eriophori* (Thüm.) Kuntze

See: *Puccinia eriophori* Thüm.

*Dicaeoma extensicola* (Plowr.) Kuntze

See: *Puccinia dioicae* Magnus. s.lat.

*Dicaeoma flosculosorum* (Alb. & Schwein.) H. Mart.

See: *Puccinia hieracii* (Röhl.) H. Mart.

*Dicaeoma fraxini* (Schwein.) Arthur

See: *Puccinia sparganioides* Ellis & Barthol.

*Dicaeoma galiorum* (Link) Arthur, nom. ambig.

See: *Puccinia punctata* Link; P. punctata var. troglodytes (Lindr.) Arthur and *P. difformis* Kunze

*Dicaeoma grossulariae* (Schumach.) H.S. Jacks.

See: *Puccinia caricina* DC., s.lat.

*Dicaeoma helianthii* (Schwein.) Kuntze

See: *Puccinia helianthi* Schwein.

*Dicaeoma heliopsidis* (Schwein.) Kuntze

See: *Puccinia helianthi* Schwein.

*Dicaeoma hibisciatum* (Schwein.) Arthur

See: *Puccinia schedonnardi* Kellerm. & Swingle

*Dicaeoma impatientis* (Schwein.) Arthur

See: *Puccinia impatientis* (Schwein.) Arthur

*Dicaeoma iridis* (DC.) Kuntze

See: *Puccinia iridis* Wallr.

*Dicaeoma kuhniae* (Schwein.) Kuntze

See: *Puccinia kuhniae* Schwein.

*Dicaeoma lateripes* (Berk. & Ravenel) Kuntze

See: *Puccinia lateripes* Berk. & Ravenel

*Dicaeoma lobeliae* (W.R. Gerard) Arthur

See: *Puccinia lobeliae* W.R. Gerard

*Dicaeoma ludibundum* (Ellis & Everh.) Kuntze

See: *Puccinia dioicae* Magnus, s.lat.

*Dicaeoma majanthae* (Schumach.) Arthur

See: *Puccinia sessilis* J. Schröt.

*Dicaeoma melicae* (P. Syd. & Syd.) Arthur

See: *Puccinia melicae* P. Syd. & Syd.

*Dicaeoma menthae* (Pers.) Gray

See: *Puccinia menthae* Pers.

*Dicaeoma minutissimum* (Arthur) H.S. Jacks.

See: *Puccinia minutissima* Arthur

*Dicaeoma montanense* (Ellis) Kuntze

See: *Puccinia montanensis* Ellis

*Dicaeoma obscurum* (J. Schröt.) Kuntze

See: *Puccinia obscura* J. Schröt.

*Dicaeoma obtectum* (Peck) Kuntze

See: *Puccinia obtecta* Peck

*Dicaeoma orbicula* (Peck & Clinton) Kuntze

See: *Puccinia orbicula* Peck & Clinton

*Dicaeoma pammelii* (Trel.) Arthur

See: *Puccinia emaculata* Schwein.

*Dicaeoma patruelis* (Arthur) H.S. Jacks.

See: *Puccinia dioicae* Magnus, s.lat.

*Dicaeoma peckii* (De Toni) Arthur

See: *Puccinia dioicae* Magnus, s.lat.

*Dicaeoma physostegiae* (Peck & Clinton) Kuntze

See: *Puccinia physostegiae* Peck & Clinton

*Dicaeoma poculiforme* (Jacq.) Kuntze

See: *Puccinia graminis* Pers.

*Dicaeoma podophylli* (Schwein.) Kuntze

See: *Allodus podophylli* (Schwein.) Arthur

*Dicaeoma polygoni-amphibii* (Pers.) Arthur

See: *Puccinia polygoni-amphibii* Pers.

*Dicaeoma polygoni-convolvuli* (Hedw.) Arthur

See: *Puccinia polygoni-amphibii* Pers.

*Dicaeoma prenanthis* (Pers.) Kuntze

See: *Puccinia orbicula* Peck & Clinton

*Dicaeoma punctatum* (Link) Arthur

See: *Puccinia punctata* Link and P. punctata var. troglodytes (Lindr.) Arthur

*Dicaeoma pustulatum* (M.A. Curtis) Arthur

See: *Puccinia andropogonis* Schwein.

*Dicaeoma ranunculi* (Seym.) Kuntze

See: *Puccinia andina* Dietel & Neger

*Dicaeoma rhamni* (J.F. Gmel.) Kuntze, nom. ambig.

See: *Puccinia coronata* Corda, s.lat., P. coronata var. avenae f. sp. avenae Urban & Marková and *P. coronati-calamagrostidis* M. Liu & Hambl.

*Dicaeoma ruelleae* (Berk. & Broome) Kuntze

See: *Puccinia lateripes* Berk. & Ravenel

*Dicaeoma sambuci* (Schwein.) Arthur

See: *Puccinia sambuci* (Schwein.) Arthur

*Dicaeoma saniculae* (Grev.) Kuntze

See: *Puccinia marylandica* Lindr.

*Dicaeoma silphii* (Schwein.) Kuntze

See: *Puccinia silphii* Schwein.

*Dicaeoma smilacis* (Schwein.) Kuntze

See: *Puccinia smilacis* Schwein.

*Dicaeoma sorghi* (Schwein.) Kuntze

See: *Puccinia sorghi* Schwein.

*Dicaeoma tenue* (Schwein.) Kuntze

See: *Puccinia tenuis* Burrill

*Dicaeoma thalictri* (Chevall.) Kuntze

See: *Tranzschelia thalictri* (Chev.) Dietel

*Dicaeoma triticinum* (Erikss.) H.S. Jacks.

See: Puccinia persistens subsp. triticina (Erikss.) Z. Urb. & J. Marková

*Dicaeoma troglodytes* (Lindr.) H.S. Jacks.

See: Puccinia punctata var. troglodytes (Lindr.) Arthur

*Dicaeoma urticae* (Schumach.) Kuntze

This name as applied by Arthur (1899) is ambiguous and probably refers to *Puccinia urticata*

See: *Puccinia urticata* F. Kern

*Dicaeoma verbenicola* (Ellis & Kellerm.) Arthur [reported as ‘*verbenicolum*’]

See: *Puccinia vilfae* Arthur & Holw.

*Dicaeoma vernoniae* (Schwein.) Kuntze (reported by Jackson 1915, but this combination was never made by Kuntze)

See: *Puccinia longipes* Lagerh.

*Dicaeoma vexans* (Farl.) Kuntze

See: *Puccinia vexans* Farl.

*Dicaeoma vilfae* (Arthur & Holw.) Arthur

See: *Puccinia vilfae* Arthur & Holw.

*Dicaeoma violae* (Schumach.) Kuntze

See: *Puccinia violae* (Schumach.) DC.

*Dicaeoma vulpinodis* (Dietel & Holw.) Kuntze

See: *Puccinia dioicae* Magnus, s.lat.

*Dicaeoma windsoriae* (Schwein.) Kuntze

See: *Puccinia windsoriae* Schwein.

*Dicaeoma xanthii* (Schwein.) Kuntze

See: *Puccinia xanthii* Schwein.

*Diorchidium lateripes* Magnus

See: Puccinia lateripes var. strepentis G.F. Laundon

*Earlea speciosa* (Fr.) Arthur

See: *Phragmidium speciosum* (Fr.) Bonard

*Endocronartium harknessii* (J.P. Moore) Y. Hirats.

See: *Cronartium harknessii* E. Meinecke

*Endophyllum tuberculatum* (Ellis & Kellerm.) Arthur & Fromme

See: *Pucciniosira tuberculata* (Ellis & Kellerm.) Buriticá & J.F. Hennen

*Frommea obtusa* (F. Strauss) Arthur

See: *Phragmidium tormentillae* Fuckel

*Frommeëlla duchesneae* (Arthur) Yohem, Cummins & Gilb.

See: *Phragmidium mexicanum* (Mains) H.Y. Yun, Minnis & Aime

Frommeëlla mexicana var. indicae J.W. McCain & J.F. Hennen

See: *Phragmidium mexicanum* (Mains) H.Y. Yun, Minnis & Aime

*Frommeëlla tormentillae* (Fuckel) Cummins & Y. Hirats.

See: *Phragmidium tormentillae* Fuckel

*Gymnoconia interstitialis* (Schltdl.) Lagerh.

See: *Gymnoconia peckiana* (Howe) Trotter

***Gymnoconia nitens* (Schwein.) Arthur**

*Rubus allegheniensis* cf. Porter

*R. allegheniensis* Porter (Rosaceae) (Jackson 1920)

R. argutus var. floridus (Tratt.) L.H. Bailey

*R. flagellaris* cf. Willd.

*R. flagellaris* Willd.; (=*R. villosus* Aiton) (Snyder 1898); (=*R. procumbens* Muhl.) (Jackson 1920)

*R. frondosus* Bigelow (=*R. sativus* Brainerd) (Jackson 1920)

*R. hispidus* L.

*R. occidentalis* L. (Jackson 1920)

*Rubus* sp.

Host not reported (Van Hook 1910)

***Gymnoconia peckiana* (Howe) Trotter**

*Rubus allegheniensis* Porter (Rosaceae) (Jackson 1915; Anderson and Anderson 1919; Emmons et al. 1960)

*R. flagellaris* Willd. (=*R. procumbens* Muhl.) (Jackson 1915); (=*R. villosus* Aiton) (Underwood 1893; Snyder 1896; Arthur 1899; Anderson and Anderson 1919)

R. idaeus L. ssp. strigosus (Michx.) Focke (=*Ru. strigosus* Michx.) (Jackson 1915)

*R. occidentalis* L. (Underwood 1893; Arthur 1899; Jackson 1915; Anderson and Anderson 1919)

*Rubus* spp. (Underwood 1893; Pipal 1915; Ruhl et al. 1980; Ruhl et al. 1981)

***Gymnosporangium bethelii* Kern**

*Crataegus cerronis* Nels. (Rosaceae) PUR: 10499

*C. coccinea* L. PUR: 10467

*C. pringlei* Sarg. PUR: 10510

*C. punctata* Jacq. PUR: 10511, 10512

*Crataegus* sp. PUR: 10525, 10521

C. chrysocarpa var. chrysocarpa Ashe (=*C. coccinea* L.)

*C. phaenopyrum* (L. f.) Medik. (=*C. cordata* (Mill.) Ait. nom. rej.) PUR: 10477, 10494, 10501

*Sorbus americana* Marshall (Rosaceae) PUR: 10531, 10532, 10533 [Doubtful record]

***Gymnosporangium biseptatum* Ellis**

*Amelanchier canadensis* (L.) Medik. (Rosaceae)

*A. ovalis* Medik. *(= A. vulgaris* Moench) Medik.

*Amelanchier × intermedia* Spach (pro sp.) [*arborea × canadensis*] Spach.

***Gymnosporangium clavariiforme* (Jacq.) DC.**

*Amelanchier canadensis* (L.) Medik. (Rosaceae) (McCain et al. 1990)

*A. erecta* Blanch.

*Amelanchier × intermedia* Spach (pro sp.) [*arborea × canadensis*] Spach.

***Gymnosporangium clavipes* Cooke & Peck**

*Amelanchier canadensis* (L.) Medik. (Rosaceae)

*A. erecta* Blanch.

*A. laevis × humilis* (Jackson 1920)

*Crataegus crus-galli* L. (Rosaceae) PUR: N11564

*C. mollis* Scheele (Jackson 1915)

*C. punctata* Jacq.

*C.* sp. (Ruhl et al. 1983)

*C. tomentosa* Anon.

*Cydonia oblonga* Mill. (=*C. vulgaris* (L.) Pers.) (Rosaceae) (Jackson 1915; Pipal 1915; Ruhl et al. 1980)

*Juniperus communis* L. (Cupressaceae)

*J. virginiana* L. (Jackson 1920; Van Hook 1921; McCain and Hennen 1981)

*Mespilus germanica* L. (Rosaceae) (Walker and Earhart 1962)

*Pyrus calleryana* Decne. (Rosaceae) (Creswell et al. 2016)

***Gymnosporangium confusum* Plowr.**

Crataegus chrysocarpa var. chrysocarpa Ashe (=*C. coccinea* L.) (Rosaceae)

***Gymnosporangium corniculans* Kern**

*Amelanchier canadensis* (L.) Medik. (Rosaceae)

***Gymnosporangium cornutum* Arthur ex F. Kern**

*Sorbus americana* Marshall (Rosaceae)

***Gymnosporangium davisii* Kern**

*Photinia melanocarpa (Michx.) K.R. Robertson & Phipps* (=*Aronia melanocarpa* (Michx.) Ellis) (Rosaceae)

*Gymnosporangium ellisii* (Berkeley) Ellis

See: *Gymnotelium myricatum* (Schwein.) Arthur

***Gymnosporangium exiguum* Kern**

*Crataegus pringlei* Sarg. (Rosaceae)

***Gymnosporangium exterum* Arthur & Kern**

*Gillenia stipulata* (Muhl. ex Willd.) Baill. (Rosaceae) (Jackson 1920; USDA 1960)

*Juniperus virginiana* L. (Cupressaceae)

***Gymnosporangium floriforme* Thaxt.**

Crataegus chrysocarpa var. chrysocarpa Ashe (=*C. coccinea* L.) (Rosaceae)

*Gymnosporangium germinale* (Schwein.) F. Kern

See: *Gymnosporangium clavipes* Cooke & Peck

***Gymnosporangium globosum* Farl.**

*Crataegus anduennae* Sarg. (Rosaceae) (Jackson 1920) [Doubtful host]

C. chrysocarpa var. chrysocarpa Ashe (=*C. coccinea* L.) (Underwood 1893; Arthur 1899; Jackson 1915; Anderson and Anderson 1919)

*C. crus-galli* L. (Arthur 1899; Jackson 1915; Anderson and Anderson 1919)

*C. erythropoda* Ashe

*Crataegus × lavallei* Hérincq ex Lavallee

*C. mollis* Scheele (Arthur 1899; Jackson 1915)

*C. pringlei* Sarg. (Jackson 1915)

*C. punctata* Jacq. (Underwood 1893; Arthur 1899; Jackson 1915; Anderson and Anderson 1919) PUR: 10771

*Crataegus* sp. (Ruhl et al. 1980, 1981; Snyder 1896; Anderson and Anderson 1919)

*C. subvillosa* Schrad. ex Torr. & A. Gray (Snyder 1898)

*Juniperus virginiana* L. (Cupressaceae) (Underwood 1893; Arthur 1899; Jackson 1915; Cooke 1967)

*Malus sylvestris* (L.) Mill. (Rosaceae)

*M. angustifolia* (Aiton) Michx. (USDA 1960)

*M. coronaria* (L.) Mill. (=*M. glaucescens* Rehd.)

*Pyrus calleryana* (Rosaceae) (PURN24059)

***Gymnosporangium inconspicuum* Kern**

*Amelanchier erecta* Blanch. (Rosaceae)

***Gymnosporangium juniperi-virginianae* Schwein.**

Ben Davis Apple (Rosaceae) (Van Hook 1924)

*Crataegus punctata* Jacq. (Rosaceae)

C. chrysocarpa var. chrysocarpa Ashe (=*C. coccinea* L.)

*Juniperus virginiana* L. (Cupressaceae) (Underwood 1893; Snyder 1896; Arthur 1899; Jackson 1915; Anderson and Anderson 1919; Emmons et al. 1960; Ruhl et al. 1981, 1983)

*Malus coronaria* (L.) Mill. (Rosaceae) (Arthur 1899; Jackson 1915); (=*Pyrus coronaria* L.) (Underwood 1893; Snyder 1896)

*M. ioensis* (Alph. Wood) Britton (Jackson 1915)

*Malus* sp. [‘CRABAPPLE’] (Ruhl et al. 1980, 1981)

*M. sylvestris* (L.) Mill. (Ruhl et al. 1980, 1982, 1983); (=*M. malus* (L.) Britton and *Pyrus malus* L.) (Arthur 1899; Jackson 1915; Pipal 1915; Anderson and Anderson 1919)

*Pyrus communis* L. (Rosaceae) (Underwood 1893; Arthur 1899) [Doubtful host, likely a *Malus* sp.]

Host not reported (Van Hook 1910)

***Gymnosporangium kernianum* Bethel**

*Amelanchier canadensis* (L.) Medik. (Rosaceae)

*Gymnosporangium libocedri* (P. Henn.) Kern

See: *Gymnotelium blasdaleanum* (Dietel & Holw.) Arthur

*Gymnosporangium macropus* Link

See: *Gymnosporangium juniperi-virginianae* Schwein.

***Gymnosporangium nelsonii* Arthur**

*Amelanchier canadensis* (L.) Medik. (Rosaceae)

*A. erecta* Blanch.

*A. ovalis (=vulgaris* Moench) Medik.

*Malus coronaria* (L.) Mill. (Rosaceae)

*M. ioensis* (Alph. Wood) Britton

*Sorbus americana* Marshall (Rosaceae)

***Gymnosporangium nidus-avis* Thaxt.**

*Malus sylvestris* (L.) Mill. (Rosaceae) (USDA 1960) [Doubtful host, likely an *Amelanchier* sp.]

*Gymnosporangium speciosum* Peck

See: *Gymnotelium speciosum* (Peck) Aime & McTaggart

***Gymnosporangium trachysorum* Kern**

*Crataegus cerronis* Nels. (Rosaceae)

*C. punctata* Jacq.

***Gymnotelium blasdaleanum* (Dietel & Holw.) Arthur**

*Amelanchier ovalis (= A. vulgaris* Moench) Medik. (Rosaceae)

*Crataegus pringlei* Sarg. (Rosaceae)

***Gymnotelium myricatum* (Schwein.) Arthur**

*Morella cerifera* (L.) Small (=*Myrica cerifera* L.) (Myricaceae)

***Gymnotelium speciosum* (Peck) Aime & McTaggart**

*Philadelphus coronarius* L. (Saxifragaceae)

***Hyalopsora polypodii* (Pers.) Magnus**

*Cystopteris fragilis* (L.) Bernh. (Cystopteridaceae) (Underwood 1893; Jackson 1915; Emmons et al. 1960)

*Kuehneola obtusa* (F. Strauss) Arthur

See: *Phragmidium tormentillae* Fuckel

***Kuehneola uredinis* (Link) Arthur**

*Rubus allegheniensis* Porter (Rosaceae) (Jackson 1915; Anderson and Anderson 1919; Emmons et al. 1960)

*R. cuneifolius* Pursh (Underwood 1893; Arthur 1899; Jackson 1915)

*R. flagellaris* Willd. (=*R. procumbens* Muhl.) (Jackson 1915); (=*R. villosus* Aiton) (Underwood 1893; Arthur 1899)

*R. hispidus* L. (Jackson 1915)

*Rubus* sp. (Pipal 1915)

***Melampsora abietis-caprearum* Tubeuf**

*Salix sericea* Marshall (Salicaceae) (USDA 1960)

*Salix* sp. (Cooke 1967)

**Melampsora aff. abietis-caprearum**

Note: Similar to *M. abietis-caprearum* but with amphigenous uredinia and telia

*Salix interior* Rowlee (PURN15351)

***Melampsora abietis-canadensis* C.A. Ludw. ex Arthur**

[Considered as a taxonomic synonym for *M. medusae* (Cellerino 1999)]

*Populus grandidentata* Michx. (Salicaceae) (Underwood 1893; Arthur 1899; Jackson 1915)

*P. heterophylla* L. (Jackson 1915)

*P. tremuloides* Michx. (Jackson 1915)

*P. deltoides* W. Bartram ex Marshall(Jackson 1915)

*Tsuga canadensis* (L.) Carriere (Pinaceae)

*Melampsora bigelowii* Thüm.

See: *Melampsora paradoxa* Diet. et Holw.

***Melampsora epitea* Thüm., sensu Hylander et al. 1953**

*Salix cordata* Michx. (Salicaceae) (Underwood 1893)

*S. eriocephala* Michx.

*S. interior* Rowlee; (=*S. longifolia* Muhl.) (Underwood 1893)

*S. nigra* Marshall (Underwood 1893)

*S. sericea* Marshall (reported as *Salix* sp.) (Snyder 1898)

*Salix* sp. (Snyder 1898)

Note: *Melampsora epitea* is a species complex comprised of at least 14 cryptic species in North America; without examination of the original material, it is not possible to determine to which precise species these reports refer (Bennett et al. 2011).

***Melampsora euphorbiae* (Schwein.) Castagne**

*Euphorbia commutata* Engelm. ex A. Gray (Euphorbiaceae)

*E. cyparissias* L. (USDA 1960)

***Melampsora euphorbiae-gerardianae* W. Müll.**

*Euphorbia commutata* Engelm. ex A. Gray (Euphorbiaceae); (=*Tithymalus commutatus* (Engelm. ex A. Gray) Klotzsch & Garcke) (Jackson 1917)

*Euphorbia* sp. (USDA 1960)

*Melampsora farinosa* (Pers.) J. Schröt., sensu Arthur 1899

Note: this is a European species and a synonym of *M. caprearum*. However, the concept defined by Arthur (1899) includes two different species viz. *M. epitea* and *M. paradoxa* and it is most likely these reports refer to one or both of these.

*Salix cordata* Michx. (Salicaceae) (Arthur 1899)

*S. discolor* Muhl. (Arthur 1899)

*S. fluviatilis* Nutt. (Arthur 1899)

*S. nigra* Marshall (Arthur 1899; Van Hook 1912)

***Melampsora ferrinii* Toome & Aime**

*Salix babylonica* L. (Salicaceae)

***Melampsora lini* (Ehrenb.) Lév.**

*Linum usitatissimum* L. (Linaceae)

***Melampsora medusae* Thüm.**

*Larix decidua* Mill. (Pinaceae) (USDA 1960)

*L. laricina* (Du Roi) K. Koch

*Populus balsamifera* L. (Salicaceae) (Underwood 1893; Jackson 1915; Anderson and Anderson 1919; Emmons et al. 1960)

*P. deltoides* Bartram ex Marshall (Arthur 1899; Jackson 1915; Anderson and Anderson 1919; Newcombe et al. 2000); ssp. monilifera (Aiton) Eckenwalder (=*P. monilifera* Aiton) (Underwood 1893; Snyder 1896)

*P. grandidentata* Michx. (Jackson 1915)

*P. nigra* L. (McCain et al. 1990)

*Populus* sp. (Anderson and Anderson 1919)

*P. tremuloides* Michx. (Underwood 1893; Snyder 1898; Arthur 1899; Jackson 1915; Anderson and Anderson 1919)

*Pseudotsuga menziesii* (Mirb.) Franco (Pinaceae)

Host not reported (Van Hook 1910)

***Melampsora paradoxa* Dietel & Holw.**

*Larix decidua* Mill. (Pinaceae) (USDA 1960)

*Salix amygdaloides* Andersson (Salicaceae) (Jackson 1915)

*S. bebbiana* Sarg. (McCain et al. 1990)

*S. caroliniana* Michx. (=*S. wardii* (Bebb) Bebb) (Jackson 1915)

*S. cordata* Michx. (Jackson 1915; Anderson and Anderson 1919)

*S. discolor* Muhl. (Underwood 1893; Snyder 1896; Jackson 1915; Anderson and Anderson 1919)

*S. interior* Rowlee (=*S. longifolia* Muhl.) (Jackson 1915; Anderson and Anderson 1919)

*S. nigra* Marshall (Anderson and Anderson 1919; Van Hook 1928)

*Salix* sp. (Anderson and Anderson 1919; USDA 1960)

*Melampsora populina* (Jacq.) Lév., sensu Underwood, 1893 [Followed by Arthur (1899)]

See: *Melampsora abietis-canadensis* C.A. Ludw. ex Arthur and *M. medusae* Thüm.

*Melampsora populina* (Jacq.) Lév.

See: *Melampsora medusae* Thüm.

*Melampsora salicina* (DC.) Lev., sensu Underwood, 1893

Note: This name as applied by Underwood (1893) could refer to either *Melampsora epitea* Thüm. or *M. paradoxa* Dietel & Holw.

*Salix* sp. (Salicaceae) (Underwood 1893)

*Melampsora salicis-capreae* (Pers.) G. Winter

See: *Melampsora paradoxa* Dietel & Holw.

***Melampsora* sp.**

*Populus* sp. (Salicaceae) (Ruhl et al. 1981)

*Melampsorella caryophyllacearum* (DC.) J. Schröt.

See: *Melampsorella elatina* (Alb. & Schwein.) Arthur

***Melampsorella elatina* (Alb. & Schwein.) Arthur**

Cerastium arvense subsp. strictum (L.) Ugborogho (Caryophyllaceae)

*Melampsoridium betulae* Arthur

See: *Melampsoridium betulinum* (Pers.) Kleb.

***Melampsoridium betulinum* (Pers.) Kleb.**

*Betula alleghaniensis* Britton (Betulaceae); var. alleghaniensis Britton (=*B. lutea* Michx. f.) (Jackson 1915; Pipal 1915; McCain et al. 1990)

*Micropuccinia andina* (Dietel & Neger) Arthur & H.S. Jacks.

See: *Puccinia andina* Dietel & Neger

***Milesia marginalis* Faull & W.R. Watson**

*Dryopteris marginalis* (L.) A. Gray (Dryopteridaceae)

*Dryopteris* sp.

***Naohidemyces vaccinii* (Jørst.) S. Sato, Katsuya & Y. Hirats. ex Vanderweyen & Fraiture**

*Tsuga canadensis* (L.) Carrière (Pinaceae) (Jackson 1920; USDA 1960)

*Nigredo amphidyma* (Syd. & P. Syd.) Arthur

See: *Uromyces amphidymus* Syd. & P. Syd.

*Nigredo appendiculata* (Pers.) Arthur

See: Uromyces appendiculatus var. appendiculatus (Pers.) Unger and *U. vignae* Barclay

*Nigredo caladii* (Schwein.) Arthur

See: *Uromyces ari-triphylli* (Schwein.) Seeler

*Nigredo caryophyllina* (Schrank) Arthur

See: *Uromyces dianthi* (Pers.) Niessl

*Nigredo fabae* (Pers.) Arthur

See: *Uromyces viciae-fabae* (Pers.) J. Schröt.

*Nigredo fallens* (Desm.) Arthur

See: Uromyces trifolii-repentis var. fallens (Desm.) Arthur.

*Nigredo hedysari-paniculati* (Schwein.) Arthur

See: *Uromyces hedysari-paniculati* (Schwein.) Farl.

*Nigredo howei* (Peck) Arthur

See: *Uromyces asclepiadis* Cooke

*Nigredo hyperici-frondosi* (Schwein.) Arthur

See: *Uromyces triquetrus* Cooke

*Nigredo lespedezae-procumbentis* (Schwein.) Arthur

See: *Uromyces lespedezae-procumbentis* (Schwein.) M.A. Curtis

*Nigredo medicaginis* (Pass.) Arthur

See: *Uromyces striatus* J. Schröt.

*Nigredo minuta* (Dietel) Arthur

See: *Uromyces minutus* Dietel

*Nigredo perigynia* (Halst.) Arthur

See: *Uromyces perigynius* Halst.

*Nigredo plumbaria* (Peck) Arthur

See: *Uromyces plumbarius* Peck

*Nigredo polygoni* (Pers.) Arthur

See: *Uromyces polygoni-avicularis* (Pers.) G.H. Otth

*Nigredo polemonii* (Peck) Arthur

See: *Uromyces acuminatus* Arthur

*Nigredo proëminens* (DC.) Arthur

See: *Uromyces proëminens* (DC.) Lév.

*Nigredo rhynchosporae* (Ellis) Arthur [as ‘*rhyncosporae*’]

See: *Uromyces rhynchosporae* Ellis

*Nigredo scirpi* (Castagne) Arthur

See: Uromyces lineolatus subsp. nearcticus Savile

*Nigredo silphii* (Burrill) Arthur

See: *Uromyces silphii* (Syd. & P. Syd.) Arthur

*Nigredo spermacoces* (Schwein.) Arthur

See: *Uromyces spermacoces* (Schwein.) M.A. Curtis

*Nigredo trifolii* (R. Hedw.) Arthur

Note: As applied by Arthur (N. American Flora 7: 255) this likely refers to *U. trifolii-repentis* (not *U. trifolii*).

See: *Uromyces trifolii-repentis* Liro and U. trifolii-repentis var. fallens (Arthur) Cummins

*Nigredo valens* (F. Kern) Arthur

See: *Uromyces valens* F. Kern

***Phakopsora crotonis* (Burrill) Arthur**

*Croton capitatus* Michx. (Euphorbiaceae) (McCain et al. 1990)

*C. monanthogynus* Michx. (McCain et al. 1990; Buriticá 1999)

***Phakopsora pachyrhizi* Syd. & P. Syd.**

*Glycine max* (L.) Merr. (Fabaceae)

***Phragmidium americanum* (Peck) Dietel**

*Rosa carolina* L. (Rosaceae) (Jackson 1915)

*R. rubiginosa* L. (Jackson 1915)

*R. setigera* Michx. (Jackson 1915; Anderson and Anderson 1919)

*R. virginiana* Mill. (Jackson 1915)

*Rosa* spp. (Jackson 1915; Pipal 1915)

*Phragmidium disciflorum* (Tode) J. James

See: *Phragmidium mucronatum* (Pers.) Schltdl.

*Phragmidium fragariae* (DC.) Rossmann

See: *Phragmidium tormentillae* Fuckel

***Phragmidium ivesiae* Syd. & P. Syd.**

*Potentilla recta* L. (Rosaceae) (Savile 1976)

***Phragmidium mexicanum* (Mains) H.Y. Yun, Minnis & Aime**

*Duchesnea indica* (Andrews) Focke (Rosaceae) (Ruhl and McCain 1986)

***Phragmidium montivagum* Arthur**

(Rosaceae) (PURN24227)

***Phragmidium mucronatum* (Pers.) Schltdl.**

*Rosa carolina* L. (Rosaceae) (Underwood 1893; Arthur 1899)

*R. humilis* Marshall (Arthur 1899)

*R. lucida* Ehrh. (Underwood 1893)

*R. setigera* Michx. (Underwood 1893; Arthur 1899)

*Rosa* spp. (Underwood 1893; Jackson 1915; Pipal 1915; Lee 1929) PUR: 8197

*Phragmidium rosae-setigerae* Dietel

See: *Phragmidium americanum* (Peck) Dietel

***Phragmidium speciosum* (Fr.) Bonard**

Rosa arkansana var. suffulta (Greene) Cockerell (=*R. suffulta* Greene) (Rosaceae)

*R. carolina* L. (Jackson 1915; Snyder 1896; Arthur 1899)

*R. humilis* Marshall (Arthur 1899)

*R. nitida* Willd.

*Rosa* sp. (Van Hook 1924)

*R. virginiana* Mill. (Jackson 1915)

*Phragmidium subcorticium* (Schrank) G. Winter

See: *Phragmidium mucronatum* (Pers.) Schltdl.

***Phragmidium tormentillae* Fuckel**

*Potentilla canadensis* L. (Rosaceae) (Underwood 1893; Snyder 1896; Arthur 1899; Jackson 1915; Anderson and Anderson 1919; Jackson 1920; Van Hook 1923, 1929)

*P. simplex* Michx. (Emmons et al. 1960; Ruhl and McCain 1986)

*Phragmidium triarticulatum* Berk. & M.A. Curtis

See: *Phragmidium tormentillae* Fuckel

***Pileolaria brevipes* Berk. & Ravenel**

*Toxicodendron pubescens* Mill. (=*Rhus toxicodendron* L.) (Anacardiaceae) (Underwood 1893; Snyder 1896; Anderson and Anderson 1919) [Doubtful host]

*T. radicans* (L.) Kuntze (=*Rhus radicans* L.) (Arthur 1899; Jackson 1915); subsp. radicans (=*R. radicans* L.) (Cooke 1967)

***Pileolaria cotini-coggygriae* Tai & Cheo**

*Cotinus coggygria* Scop. (Kaishian et al. 2023; PUR058489)

*Pileolaria toxicodendri* (Berk. & Ravenel) Arthur

See: *Pileolaria brevipes* Berk. & Ravenel

*Polythelis fusca* Arthur

See: *Tranzschelia pseudofusca* M. Scholler & M. Abbasi

*Polythelis thalictri* (Chevall.) Arthur

See: *Tranzschelia thalictri* (Chev.) Dietel

***Prospodium transformans* (Ellis & Everh.) Cummins**

*Tecoma stans* (L.) Juss. ex Kunth (Bignoniaceae)

***Puccinia acetosae* (Schumach.) Körn.**

*Rumex acetosella* L. (Polygonaceae) (Jackson 1917; USDA 1960)

*Puccinia albiperidia* Arthur

See: *Puccinia caricina* DC., s.lat.

***Puccinia aletridis* Berk. & M.A. Curtis**

*Aletris farinosa* L. (Nartheciaceae) (Jackson 1915; USDA 1960)

***Puccinia amphigena* Dietel**

*Ammophila breviligulata* Fernald (Poaceae) (USDA 1960)

*Calamovilfa longifolia* (Hook.) Scribn. (Poaceae) (Cummins and Greene 1961); var. magna Scribn. & Merr. (McCain et al. 1990)

*Smilax herbacea* L. (Smilacaceae) (Cummins and Greene 1961)

*S. tamnoides* L. (=*hispida* Muhl. ex Torr.) (Cummins and Greene 1961)

***Puccinia andina* Dietel & Neger**

Ranunculus hispidus var. nitidus (Chapm.) T. Duncan (=*R. septentrionalis* Poir.) (Ranunculaceae) (Arthur 1899; Jackson 1915; USDA 1960)

*Puccinia andropogi* Schwein.

See: *Puccinia andropogonis* Schwein

***Puccinia andropogonis* Schwein.**

*Aesculus glabra* Willd. (Hippocastanaceae)

*A. hippocastanum* L.

*Andropogon gerardii* Vitman (Poaceae); (=*A. furcatus* Muhl. ex Willd.) (Snyder 1896; Arthur 1899; Jackson 1915)

*Andropogon* sp. (Underwood 1893)

*Comandra umbellata* (L.) Nutt. (Santalaceae) (Underwood 1893; Jackson 1915; Anderson and Anderson 1919)

*Orbexilum onobrychis* (Nutt.) Rydb. (=*Psoralea onobrychis* Nutt.) (Fabaceae) (Underwood 1893)

*Penstemon digitalis* Nutt. ex Sims (Plantaginaceae)

*P. hirsutus* (L.) Willd. (Jackson 1915; USDA 1960) PUR: 88314

*P. laevigatus* Aiton; (=*P. penstemon* (L.) Britton) (Jackson 1920)

P. glaber var. alpinus (Torr.) A. Gray (=*P. alpinus* Torr.)

*P. pubescens* Aiton [as ‘*Pentstemon pubescens*’] (Snyder 1896)

*Schizachyrium scoparium* (Michx.) Nash (Poaceae) (Jackson 1915); (=*Andropogon scoparius* Michx., as [‘*scoparus*’]) (Snyder 1896; Arthur 1899)

*Zanthoxylum americanum* Mill. (Rutaceae) (USDA 1960; McCain et al. 1990)

Puccinia andropogonis var. pentastemonis (Schwein.) Arthur

See: *Puccinia andropogonis* Schwein.

***Puccinia anemones-virginianae* Schwein.**

*Anemone canadensis* L. (Ranunculaceae) (Cooke 1967; McCain and Hennen 1981)

*A. cylindrica* A. Gray (Snyder 1896; Arthur 1899; Jackson 1915)

*Anemone* sp.

*A. virginiana* L. (Jackson 1915; Anderson and Anderson 1919)

***Puccinia angelicae* (Schumach.) Fuckel**

*Taenidia integerrima* (L.) Drude (Apiaceae) (Jackson 1915; USDA 1960)

***Puccinia angustata* Peck**

*Eriophorum virginicum* L. (Cyperaceae)

*E. viridicarinatum* (Engelm.) Fernald

*Lycopus americanus* Muhl. ex W.P.C. Barton (=*L. sinuatus* Elliott) (Lamiaceae) (Snyder 1898; Jackson 1915)

*Lycopus* sp. (Underwood 1893)

*L. uniflorus* Michx. (Jackson 1915)

*Pycnanthemum virginianum* (L.) T. Dur. & B.D. Jacks. ex B.L. Rob. & Fernald (Lamiaceae) (USDA 1960); (=*P. lanceolatum* Pursh); (=*Koellia virginiana* (L.) MacMill.) (Jackson 1920)

*Scirpus atrovirens* Willd. (Cyperaceae) (Underwood 1893; Snyder 1896; Arthur 1899; Jackson 1915; Anderson and Anderson 1919; Savile 1972)

*S. cyperinus* (L.) Kunth (Arthur 1899); (=*Eriophorum cyperinum* L.) (Underwood 1893)

*Scirpus* sp.

*Puccinia anomala* Rostr.

See: *Puccinia hordei* G.H. Otth

***Puccinia antirrhini* Dietel & Holw.**

*Antirrhinum majus* L. (Plantaginaceae) (Jackson 1915; Pipal 1915; Anderson and Anderson 1919) PUR: 42777, 42487, 42489,42490, 42492, 42493

*Antirrhinum* sp. PUR: N10830

***Puccinia apocrypta* Ellis & Tracy**

*Asperella hystrix* (L.) Humb. in Roem. & Usteri (Poaceae) (Underwood 1893)

*Elymus hystrix* L. (=*Hystrix patula* Moench) (Poaceae) (USDA 1960); var. hystrix (=*H. hystrix* (L.) Millsp.) (Arthur 1899)

***Puccinia argentata* (Schultz) G. Winter**

*Impatiens capensis* Meerb. (=*I. fulva* Nutt.) (Balsaminaceae) (Snyder 1896); (=*I. biflora* Walter) (Arthur 1899; Jackson 1915)

*I. flava* (Underwood 1893) [Doubtful host, likely refers to *I. fulva*]

*I. capensis* Meerb. (reported as *I. flava*) (Underwood 1893)

*I. pallida* Nutt. (USDA 1960)

*Puccinia aristidae* Tracy

See: *Puccinia subnitens* Dietel

***Puccinia arundinariae* Schweinitz**

*Smilax* sp. (Smilacaceae) (Cummins 1971) PUR: 45961

***Puccinia asparagi* DC.**

*Allium cepa* L. (Amaryllidaceae) (Ruhl et al. 1983) [Doubtful host]

*Asparagus officinalis* L. (Asparagaceae) (Jackson 1915; Anderson and Anderson 1919; Ruhl et al. 1980)

*Asparagus* sp. (Pipal 1915)

Host not reported (Van Hook 1910)

*Puccinia asperifolii* (Pers.) Wettst. [as’ *asperifolia*’ by Pipal (1915)]

See: *Puccinia recondita* Roberge ex Desm. s.str.

*Puccinia asteris* Duby

See: *Puccinia cnici-oleracei* Pers. Ex Desm.

*Puccinia asterum* (Schwein.) F. Kern

See: *Puccinia dioicae* Magnus, s.lat.

***Puccinia atrofusca* (Dudley & C.H. Thomps.) Holw.**

*Artemisia* (=*Dracunculoides* L.) *dracunculus* L. (Asteraceae)

*Artemisia* sp.

***Puccinia bardanae* (Wallr.) Corda**

*Arctium lappa* L. (Asteraceae) (Jackson 1915; Anderson and Anderson 1919) [Doubtful host, likely refers to *A. minus*]

*A. minus* (Hill) Bernh. (Savile 1970); PUR: N2751, 38010, 38119, 38120, 38124, 38125, 38126, 38141, 38143, 38154, 49274, 61540, 88329, 89269, N1943, N4508, N4509, N6319

*Puccinia bolleyana* Saccardo

See: *Puccinia sambuci* (Schwein.) Arthur

*Puccinia brachypodii* G.H. Otth

See: *Puccinia poae-nemoralis* G.H. Otth

Puccinia brachypodii var. arrhenatheri (Kleb.) Cummins & H.C. Greene

See: *Puccinia magelhaenica* Peyr.

Puccinia brachypodii var. poae-nemoralis (G.H. Otth) Cummins & H.C. Greene

See: *Puccinia poae-nemoralis* G.H. Otth

***Puccinia bromina* Erikss., s.lat.**

*Bromus pubescens* Muhl. ex Willd. (Poaceae) PUR: 25031, 25039, 25041

*B. secalinus* L. PUR: 25083

Puccinia calcitrapae var. bardanae (Wallr.)

See: *Puccinia bardanae* (Wallr.) Corda

Puccinia calcitrapae var. centaureae (DC.) Cummins

See: Puccinia laschii var. laschii Lagerh.

***Puccinia calthae* Link**

*Caltha palustris* L. (Ranunculaceae) (Jackson 1915)

***Puccinia canaliculata* (Schwein.) Lagerh.**

*Ambrosia trifida* L. (Asteraceae) (1, Jackson 1915; Kern 1919)

*Cyperus esculentus* L. (Cyperaceae) (Jackson 1915; Kern 1919; Van Hook 1929)

*C. filiculmis* Vahl. (Jackson 1915)

*C. odoratus* L.; (=*Cyperus engelmannii* Steud.) (Cyperaceae) (Jackson 1915); (=*C. speciosus* Vahl) (Jackson 1920)

*Cyperus* sp.

*C. strigosus* L. (Snyder 1896; Jackson 1915; Kern 1919)

*C. schweinitzii* Torr. (Jackson 1915)

Xanthium strumarium L. var. canadense (Mill.) Torr. & A. Gray (=*X. commune* Britton) (Asteraceae) (Jackson 1915); var. glabratum (DC.) Cronquist (=*X. americanum* Walter) (Jackson 1915)

*Xanthium* sp. (Kern 1919)

*Puccinia caricis* (Schumach.) Rebent.

See: *Puccinia caricina* DC.

***Puccinia caricina* DC., s.lat.**

*Carex blanda* Dewey (Cyperaceae)

*C. bullata* Schkuhr ex Willd. (Cyperaceae) (Underwood 1893)

*C. cephalophora* Muhl. ex Willd. (Jackson 1920)

*C. conoidea* Schkuhr ex Willd. (Jackson 1920)

*C. crinita* Lam. (Jackson 1915)

*C. davisii* Schwein & Torr. (McCain and Hennen 1981)

*C. digitalis* Willd. (Jackson 1915)

*C. emoryi* Dewey

*C. frankii* Kunth (Snyder 1898)

*C. gracilescens* Steud. (McCain and Hennen 1981)

*C. hirtifolia* Mack. (Jackson 1915) PUR: 27716; (=*C. pubescens* Muhl. ex Schkuhr) (Anderson and Anderson 1919; Jackson 1920)

*C. hitchcockiana* Dewey (Jackson 1915)

*C. laxiflora* Lam. (Jackson 1915)

*C. lupuliformis* Sartwell ex Dewey (Jackson 1920)

*C. lurida* Wahlenb. (Underwood 1893)

*C. rosea* Schkuhr ex Willd.

*C. siccata* Dewey (=*Carex foenea* Willd.) (Underwood 1893)

*Carex* sp. (Snyder 1896)

*C. sparganioides* Muhl. ex Willd. (Jackson 1920)

*C. squarrosa* L. (Jackson 1915)

*C. stricta* Lam. (Jackson 1915)

*C. tetanica* Schkuhr (Jackson 1915)

*C. trichocarpa* Muhl. ex Willd.

*Ribes americanum* Mill. [as’*americana*’] (Grossulariaceae) (Jackson 1920)

*R. aureum* Pursh

*R. cynosbati* L. (Underwood 1893; Snyder 1898; Anderson and Anderson 1919; McCain and Hennen 198; PUR: 27120 – Greenhouse)

*R. cynosbati* (=*Grossularia cynosbati* (L.) Mill.) (Jackson 1915)

*R. floridum* L’Herit (Anderson and Anderson 1919) [Doubtful host]

*R. hirtellum* Michx.; (=*G. hirtella* (Michx.) Spach) (Jackson 1920)

*R. missouriense* Nutt. (=*G. missouriensis* (Nutt.) Coville & Britton) (Jackson 1915)

R. oxyacanthoides L. ssp. oxyacanthoides (=*G. oxyacanthoides* (L.) Mill.) (Jackson 1915; Anderson and Anderson 1919); ssp. setosum (Lindl.) Sinnott (=*G. setosa* (Lindl.) Coville & Britton) (Jackson 1915)

*R. rotundifolium* Michx. (Underwood 1893); (=*G. rotundifolia* (Michx.) Coville & Britton) (Jackson 1915)

*R. rubrum* L. (=*R. vulgare* Lam.) (Jackson 1920)

*Ribes* sp. (Van Hook 1929)

*R. uva-crispa* L.

**Puccinia caricina var. limosae (Magnus) Jørst.**

*Lysimachia thyrsiflora* L. (=*Naumburgia thyrsiflora* (L.) Duby) (Primulaceae) (Jackson 1917)

*Puccinia caricis-erigerontis* Arthur

See: *Puccinia dioicae* Magnus, s.lat.

***Puccinia caulicola* Tracy & Gall.**

*Salvia lanceolata* Lam. (Lamiaceae)

***Puccinia chloridis* Speg.**

*Asclepias incarnata* L. (Apocynaceae)

*A. syriaca* L.

*Puccinia chrysanthemi* Roze

See: Puccinia tanaceti var. tanaceti DC.

***Puccinia circaeae* Pers.**

*Circaea lutetiana* L. (Onagraceae) (Underwood 1893; Snyder 1896; Arthur 1899; Jackson 1915; Anderson and Anderson 1919); subsp. canadensis (L.) Asch. & Magnus (=*C. quadrisulcata* (Maxim.) Franch. & Savigny) (Emmons et al. 1960)

*Puccinia cirsii* Lasch

See: Puccinia laschii var. laschii Lagerh.

*Puccinia clematidis* (DC.) Lagerh., nom. ambig.

*Anemone virginiana* L. (Ranunculaceae) (Jackson 1920)

*Pascopyrum smithii* (Rydb.) Á. Löve (=*Agropyron smithii* Rydb.) (Poaceae) (Jackson 1920)

**Puccinia cnici var. cnici H. Mart.**

*Cirsium lanceolatum* Hill (Asteraceae) (Jackson 1915; Anderson and Anderson 1919)

*C. vulgare* (Savi) Ten. (Savile 1970)

***Puccinia cnici-oleracei* Pers. ex Desm.**

*Aster* sp. (Asteraceae) (Underwood 1893; Snyder 1898; Anderson and Anderson 1919)

*Calendula officinalis* L. (Asteraceae) PUR: 42243 - Greenhouse

*Dimorphotheca cuneata* (Thunb.) Less. (Asteraceae)*

*Symphyotrichum cordifolium* (L.) G.L. Nesom (=*Aster cordifolius* L.) (Asteraceae) (Underwood 1893; Arthur 1899; Jackson 1915; Anderson and Anderson 1919); (=*A. sagittifolius* Wedemeyer ex Willd.) (Arthur 1899; Jackson 1915)

*S. lanceolatum* (=*A. paniculatus* Lam.) (Arthur 1899); subsp. lanceolatum var. lanceolatum (Willd.) G.L. Nesom (=*A. paniculatus* Lam.) (Underwood 1893; Jackson 1915; Anderson and Anderson 1919); subsp. lanceolatum var. lanceolatum (=*A. diffusus*) (Snyder 1896)

S. lateriflorum var. lateriflorum (L.) Á. Löve & D. Löve (=*A. lateriflorus* (L.) Britton) (Arthur 1899; Jackson 1920)

*S. novae-angliae* (L.) G.L. Nesom (=*A. novae-angliae* L.) (Jackson 1915)

S. novi-belgii (L.) G.L. Nesom var. novi-belgii (=*A. longifolius* Lam.) (Jackson 1915)

S. oolentangiense var. oolentangiense (Riddell) G.L. Nesom (=*A. azureus* Lindl.) (Jackson 1915)

S. praealtum var. praealtum (Poir.) G.L. Nesom (=*A. salicifolius* Aiton) (Arthur 1899; Jackson 1920)

S. puniceum var. puniceum (L.) Á. Löve & D. Löve (=*A. puniceus* L.) (Jackson 1915)

*S. shortii (Lindl.)* G.L. Nesom (=*A shortii* Lindl.) (Arthur 1899)

***Puccinia cockerelliana* Bethel ex Arthur**

*Thalictrum dioicum* L. (Ranunculaceae)

**Puccinia conoclinii var. conoclinii Seym. ex. Burrill**

*Conoclinium coelestinum* (L.) DC. (=*Eupatorium coelestinum* (L.) DC) (Asteraceae) (Jackson 1915)

*Eupatorium* sp. (Asteraceae) (USDA 1960)

*Fleischmannia incarnata* (Walter) R.M. King & H. Rob. (=*E. incarnatum* Walter) (Asteraceae) (Jackson 1920; Cummins et al. 1969)

***Puccinia convolvuli* (Pers.) Castagne**

*Calystegia sepium* (L.) R. Br. (Convolvulaceae) (Jackson 1915); (=*Convolvulus sepium* L.) (Underwood 1893; Snyder 1896; Arthur 1899; Anderson and Anderson 1919); [reported as *Polygonum dumetorum* L.] (Snyder 1896)

*Convolvulus arvensis* L. (Convolvulaceae) (USDA 1960)

*Convolvulus* sp.

***Puccinia coronata* Corda, s.lat.**

*Alopecurus aequalis* Sobol. (Poaceae) (USDA 1960); (= *A. aristulatus* Michx.) (Jackson 1920)

*Cinna arundinacea* L. (Poaceae) (Jackson 1915; USDA 1960)

*Frangula caroliniana* (Walter) A. Gray (=*Rhamnus caroliniana* Walter) (Rhamnaceae) (Jackson 1915; USDA 1960)

*F. purshiana* (DC.) A. Gray

*Hordeum jubatum* L. (Poaceae)

*Rhamnus alnifolia* L’Hér. (Rhamnaceae) (Jackson 1920)

*R. cathartica* L. (Van Hook 1925)

*R. lanceolata* Pursh (Arthur 1899; USDA 1960)

Host not reported (Van Hook 1910)

Also see: Puccinia coronata var. avenae f. sp. avenae Urban & Marková, *P. coronati-brevispora* M. Liu & Hambl.

**Puccinia coronata var. avenae f. sp. avenae Urban & Marková**

*Avena sativa* L. (Poaceae) (Snyder 1896, 1898; Arthur 1899; Jackson 1915; Pipal 1915; Anderson and Anderson 1919; PUR: N1190 – Greenhouse)

*Lolium perenne* L. (Poaceae)

*Lolium* sp. (Poaceae)

*Poa pratensis* L. (Poaceae)

***Puccinia coronatae-brevispora* M. Liu & Hambl.**

*Bromus* sp. (Poaceae) PUR: N11047

Puccinia coronata var. avenae Fraser & Ledinham

See: Puccinia coronata var. avenae f. sp. avenae Urban & Marková

***Puccinia coronatae-calamagrostidis* M. Liu & Hambl. [as ‘ *coronati-calamagrostidis*** ’]

*Calamagrostis canadensis* (Michx.) P. Beauv. (Poaceae) (Underwood 1893; Arthur 1899; Jackson 1915)

***Puccinia crandallii* Pammel & H.H. Hume**

*Symphoricarpos albus* (L.) S.F. Blake (Caprifoliaceae)

**Puccinia cyani var. cyani Pass.**

*Centaurea cyanus* L. (Asteraceae) (USDA 1960; Savile 1970)

***Puccinia cyperi* Arthur**

*Conyza canadensis* (L.) Cronquist (Asteraceae)

*Cyperus echinatus* (L.) Alph. Wood (=*C. ovularis* (Michx.) Torr.) (Cyperaceae) (McCain et al. 1990)

C. lupulinus subsp. lupulinus (Spreng.) Marcks (=*C. filiculmis* Vahl) (Kern 1919; Jackson 1920); subsp. macilentus (Fernald) Marcks (=C. filiculmis var. macilentus Fernald) (McCain et al. 1990)

*C. schweinitzii* Torr. (Kern 1919; Jackson 1920)

*Cyperus* sp. (Underwood 1893)

*C. strigosus* L. (Underwood 1893; Snyder 1896; Arthur 1899; Anderson and Anderson 1919; Kern 1919; Lopez-Franco and Hennen 1990)

*Erigeron annuus* (L.) Pers. (Asteraceae)

*E. strigosus* Muhl. ex Willd.

***Puccinia cypripedii* Arthur**

*Calopogon tuberosus* (L.) Britton, Sterns & Poggenb. (Orchidaceae); var. tuberosus (=*Limodorum tuberosum* L.) (Jackson 1915)

*Calopogon* sp. (McCain et al. 1990)

***Puccinia dayi* Clinton**

*Lysimachia ciliata* L. (Primulaceae); (=*Steironema ciliatum* (L.) Baudo) (Primulaceae) (Underwood 1893; Arthur 1899; Jackson 1915; Anderson and Anderson 1919)

***Puccinia difformis* Kunze**

*Galium aparine* L. (Rubiaceae) (Arthur 1899; Jackson 1915)

***Puccinia dioicae* Magnus, s.lat.**

*Agoseris glauca* (Pursh) Raf. (Asteraceae)

*Brachychaeta sphacelata* (Raf.) Britton (Asteraceae) (Jackson 1920)

*Calylophus serrulatus (Nutt.) P.H. Raven* (=*Oenothera serrulata* Nutt.) (Onagraceae)

Carex albicans Willd. ex Spreng. var. albicans (=*C. varia* Muhl. ex Willd.) (Cyperaceae) (Jackson 1920)

*C. annectens* (E.P. Bicknell) E.P. Bicknell (McCain and Hennen 1981; Schoknecht 1981)

*C. brevior* (Dewey) Mack.

*C. cephaloidea* (Dewey) Dewey (Jackson 1915)

*C. cephalophora* Muhl. ex Willd. (Jackson 1915)

Carex cf. frankii Kunth

*C. communis* L.H. Bailey (McCain and Hennen 1981)

*C. conoidea* Schkuhr ex Willd. (Jackson 1915)

*C. festucacea* Schkuhr ex Willd. (Jackson 1915)

*C. jamesii* Schwein.

*C. laeviculmis* Meinsh. (Cyperaceae)

*C. laeviconica* Dewey (Jackson 1920)

*C. lanuginosa* Michx. (Jackson 1915)

*C. lupuliformis* Sartwell ex Dewey (Jackson 1920)

*C. muskingumensis* Schwein. (Jackson 1920)

*C. nebrascensis* Dewey (Jackson 1915)

*C. normalis* Mack. (Jackson 1920)

*C. oligocarpa* Schkuhr ex Willd. (Jackson 1915)

*C. pellita* Muhl. ex Willd. (=*C. lanuginosa* auct. non Michx.)

*C. pensylvanica* Lam. (Jackson 1915; USDA– inoculation using aeciospores from *Dirca palustris*)

*C. rosea* Schkuhr (McCain and Hennen 1981)

*C. siccata* Dewey (=*C. foenea* Willd.) (Jackson 1915)

*Carex* sp. (Underwood 1893; Jackson 1915; Cooke 1967)

*C. sparganioides* Muhl. ex Willd. (Snyder 1896; Arthur 1899; Arthur 1909; Jackson 1915)

*C. stenolepis* Less. ex Steud. (Underwood 1893)

*C. stipata* Muhl. ex Willd. (Arthur 1909; Jackson 1915; Jackson 1920)

*C. straminea* Willd. ex Schkuhr (Underwood 1893; Jackson 1915)

*C. tetanica* Schkuhr (Jackson 1915)

*C. tribuloides* Wahlenb. (Jackson 1920)

*C. trichocarpa* Muhl. ex Willd. (Jackson 1915)

*C. umbellata* Schkuhr ex Willd. (=*C. abdita* E.P. Bicknell) (Jackson 1920)

*C. virescens* Muhl. ex Willd. (Underwood 1893)

*C. vulpinoidea* Michx. (Cyperaceae) (Underwood 1893; Snyder 1896; Arthur 1899; Arthur 1909; Jackson 1915)

*C. willdenowii* Schkuhr ex Willd. (Jackson 1920)

Conyza canadensis var. canadensis (L.) Cronquist (=*Erigeron canadensis* L.) (Asteraceae); (=*Leptilon canadense* (L.) Britton) (Jackson 1915)

*Dirca palustris* L. (Thymelaeaceae) (Underwood 1893; Jackson 1917; Anderson and Anderson 1919; Van Hook 1927) PUR: 24 specimens

*Dulichium arundinaceum* (L.) Britton (Cyperaceae) (Jackson 1915)

*D. spathaceum* Rich. (Underwood 1893)

*Erigeron annuus* (L.) Pers. (Asteraceae) (Jackson 1915; Anderson and Anderson 1919) PUR: 28800

*E. pulchellus* Michx. (Jackson 1920)

E. strigosus var. strigosus Muhl. ex Willd. (=*E. ramosus* (Walter) Britton, Sterns & Poggenb.) (Jackson 1915)

*Euthamia graminifolia* (L.) Nutt. (Asteraceae)

*Lactuca canadensis* L. (Asteraceae) (Jackson 1915; Anderson and Anderson 1919; Jackson 1920)

*L. floridana* (L.) Gaertn. (Jackson 1915) [Doubtful host, likely is *L. canadensis*]

*L. sativa* L. (Jackson 1915; USDA 1960)

*L. virosa* L. (Jackson 1915)

*Oenothera biennis* L. (Onagraceae) (Underwood 1893; Snyder 1896; Jackson 1915; Anderson and Anderson 1919)

*Phryma leptostachya* L. (Phrymaceae)

*Sericocarpus* sp. (Asteraceae) (McCain et al. 1990)

*Solidago altissima* L. (Asteraceae) (Jackson 1920)

*S. arguta* Aiton (Anderson and Anderson 1919)

*S. caesia* L. (Underwood 1893; Jackson 1915; Anderson and Anderson 1919)

*S. canadensis* L. (Underwood 1893; Jackson 1915; Savile 1972)

*S. flexicaulis* L. (Jackson 1915); (=*S. latifolia* L.) (Underwood 1893)

*S. gigantea* Aiton (=*S. serotina* Aiton) (Jackson 1915)

*S. juncea* Aiton

*S. patula* Muhl. ex Willd. (Jackson 1915) PUR: 88315

*S. rugosa* Mill.

*Solidago* sp. (Underwood 1893; McCain and Hennen 1981)

*S. ulmifolia* Muhl. ex Willd. (Jackson 1915)

S. missouriensis var. fasciculata Holz. (=*S. glaberrima* M. Martens)

*Symphyotrichum cordifolium* (L.) G.L. Nesom (=*Aster cordifolius* L.) (Asteraceae) (Underwood 1893; Jackson 1915; Anderson and Anderson 1919); (=*A. sagittifolius* Wedemeyer ex Willd.) (Underwood 1893; Jackson 1915; Anderson and Anderson 1919)

S. drummondii var. drummondii (Lindl.) G.L. Nesom (=*A. drummondii* Lindl.) (Jackson 1915)

S. ericoides var. ericoides (L.) G.L. Nesom (=*A. ericoides* L.)

S. foliaceum var. canbyi (A. Gray) G.L. Nesom (=*A. tweedyi* Rydb.)

S. lanceolatum subsp. lanceolatum var. lanceolatum (Willd.) G.L. Nesom (=*A. paniculatus* Lam.) (Jackson 1915)

S. praealtum var. praealtum (Poir.) G.L. Nesom (=*A. salicifolius* Aiton, non Lam.)

S. puniceum var. puniceum (L.) Á. Löve & D. Löve (=*A. puniceus* L.) (Jackson 1920)

*S. shortii* (Lindl.) G.L. Nesom (=*A. shortii* Lindl.) (Anderson and Anderson 1919)

*Puccinia dispersa* Erikss. & Henning

See: *Puccinia recondita* Roberge ex Desm. s.str.

***Puccinia distichlidis* Ellis & Everh.**

*Lysimachia ciliata* L. (Primulaceae)

***Puccinia dochmia* Berk. & M.A. Curtis**

Note: Doubtful record, likely refers to *Puccinia schedonnardi*

*Muhlenbergia diffusa* (Muhl.) Farw. (Poaceae) (Underwood 1893; Arthur 1899)

*M. sylvatica* (Torr.) Torr. ex A. Gray (Arthur 1899)

*Puccinia dulichii* P. Syd. & Syd.

See: *Puccinia dioicae* Magnus, s.lat.

***Puccinia eatoniae* Arthur**

*Myosotis verna* Nutt. (=*M. virginica* (L.) Britton, Sterns & Poggenb.) (Boraginaceae) (USDA 1960)

*Ranunculus abortivus* L. (Ranunculaceae) (Underwood 1893; Jackson 1915; Anderson and Anderson 1919)

*Sphenopholis intermedia* (Rydb.) Rydb. (Poaceae)

*S. obtusata* (Michx.) Scribn.

*Sphenopholis* sp.

*Sphenopholis × pallens* (Biehler) Scribn. (pro sp.) [*obtusata × pensylvanica*] (Jackson 1915)

***Puccinia eleocharidis* Arthur**

*Ageratina altissima* (L.) R.M. King & H. Rob. (Asteraceae)

*Eupatorium maculatum* (L.) E.E. Lamont (Asteraceae) (Jackson 1915; Kern 1919)

*E. perfoliatum* L. (Jackson 1915; Kern 1919)

*E. purpureum* (L.) E.E. Lamont (Snyder 1897; Jackson 1915; Kern 1919) PUR: 26729

*Eleocharis obtusa* (Willd.) Schult. (Cyperaceae) (Savile 1972; Jackson 1920; Kern 1919)

*Eleocharis palustris* (L.) Roem. & Schult. (Underwood 1893; Snyder 1896; Arthur 1899; Arthur 1909; Jackson 1915; Anderson and Anderson 1919; Kern 1919)

***Puccinia ellisiana* Thüm.**

*Schizachyrium scoparium* (Michx.) Nash (Poaceae) (Jackson 1915)

*Viola nuttallii* Pursh (Violaceae)

V. palmata L. var. cucullata A. Gray (Underwood 1893)

*V. sororia* Willd.; (=*Viola papilionacea* Pursh) (Jackson 1915)

***Puccinia emaculata* Schwein.**

*Euphorbia corollata* L. (Euphorbiaceae); (=*Tithymalopsis corollata* (L.) Klotzsch) (Jackson 1915)

*E. marginata* Pursh

*Panicum capillare* L. (Poaceae) (Underwood 1893; Snyder 1896; Arthur 1899; Jackson 1915; Anderson and Anderson 1919; Ramachar and Cummins 1965; Demers et al. 2017)

*P. flexile* (Gattinger) Scribn.

*P. miliaceum* L. (Jackson 1915; USDA 1960)

*Panicum* sp. (USDA 1960)

*P. virgatum* L. (Jackson 1915)

*Puccinia epiphylla* (L.) Wettst.

See: *Puccinia poarum* Nielsen

***Puccinia eriophori* Thüm.**

*Eriophorum angustifolium* Honck. (Cyperaceae) (Jackson 1915)

*E. virginicum* L. (Jackson 1915)

***Puccinia eumacrospora* Cummins**

*Smilax tamnoides* L. (=*S. hispida* Muhl. ex Torr.) (Smilacaceae)

*Puccinia extensicola* Plowr.

See: *Puccinia dioicae* Magnus, s.lat.

Puccinia extensicola var. asteris (Thüm.) Arthur

See: *Puccinia dioicae* Magnus, s.lat.

Puccinia extensicola var. hieraciata (Schwein.) Arthur

See: *Puccinia dioicae* Magnus, s.lat.

Puccinia extensicola var. solidaginis Arthur

See: *Puccinia dioicae* Magnus, s.lat.

*Puccinia flaveriae* H.S. Jacks.

See: *Puccinia melampodii* Dietel & Holw.

*Puccinia flosculosorum* Röhl.

See: *Puccinia hieracii* (Röhl.) H. Mart.

*Puccinia fraxinata* (Link) Arthur

See: *Puccinia sparganioides* Ellis & Barthol.

*Puccinia galii* (Pers.) Schwein.

See: *Puccinia punctata* Link and P. punctata var. troglodytes (Lindr.) Arthur

***Puccinia gentianae* (F. Strauss) Link**

*Gentiana andrewsii* Griseb. (Gentianaceae)

***Puccinia glechomatis* DC.**

*Agastache nepetoides* (L.) Kuntze (Lamiaceae) (Jackson 1915; Anderson and Anderson 1919)

*Glechoma hederacea* L. (Lamiaceae) (Scholler 2000; Böllmann and Scholler 2006)

***Puccinia globosipes* Peck**

*Lycium barbarum* L. (=*L. halimifolium* Mill.) (Solanaceae) (Jackson 1915); (=*halimifolium* Mill.; *vulgare* Dunal) (Cash 1952)

*Puccinia globosum* (Farl.) Kuntze

See: *Gymnosporangium globosum* Farl.

***Puccinia goldsbroughii* M. Abbasi & Aime**

*Melica nitens* (Scribn.) Nutt. ex Piper (Poaceae) (Abbasi and Aime 2016)

*Melica* sp. (Poaceae) (Abbasi and Aime 2016)

***Puccinia graminicola* (Burrill) Demers & Castl.**

*Panicum virgatum* L. (Poaceae) (Arthur 1899; Jackson 1920; Kenaley et al. 2018) PUR: 11737

Unknown grasses (Poaceae) (Underwood 1893)

***Puccinia graminis* Pers.**

*Agrostis alba* L. (Poaceae) (Jackson 1915; Anderson and Anderson 1919)

*A. gigantea* Roth

*Agrostis* sp. (Underwood 1893; Arthur 1899)

*Alopecurus aequalis* Sobol. (Poaceae) (USDA 1960); var. aequalis Sobol. (=*A. aristulatus* Michx.) (Jackson 1920)

*A. pratensis* L.

*Arrhenatherum elatius* (L.) P. Beauv. ex J. Presl & C. Presl (Poaceae) (Jackson 1915; USDA 1960)

*Avena sativa* L. (Poaceae) (Underwood 1893; Snyder 1896; Arthur 1899; Jackson 1915; Anderson and Anderson 1919)

*Berberis canadensis* Mill. (Berberidaceae)

*B. vulgaris* L. (Underwood 1893; Arthur 1899; Jackson 1915; Jackson 1920)

*Bromus carinatus* Hook. & Arn. (Poaceae)

*B. ciliatus* L.

*B. kalmii* A. Gray (=*Bromus purgans* L.) (Jackson 1920; USDA 1960)

*B. marginatus* Nees ex Steud. PUR: N5237 - Greenhouse

*B. secalinus* L. (Jackson 1915)

*B. tomentellus* Boiss. PUR: N5238 - Greenhouse

*Cinna arundinacea* L. (Poaceae) (Jackson 1915; USDA 1960)

*Dactylis glomerata* L. (Poaceae) (Snyder 1896; Arthur 1899; Jackson 1915; USDA 1960; Cooke 1986)

Elymus alaskanus subsp. latiglumis (Scribn. & J.G. Sm.) Á. Löve (Poaceae)

*E. canadensis* L. (Jackson 1920)

*E. elymoides* (Raf.) Swezey

*E. repens* (L.) Gould; (=*Agropyron repens* (L.) P. Beauv.) (Jackson 1920)

*E. trachycaulus* (Link) Gould ex Shinners

*E. virginicus* L. (Jackson 1920)

*Festuca idahoensis* Elmer (Poaceae)

*Hordeum jubatum* L. (Poaceae) (Snyder 1896; Arthur 1899; Jackson 1915) PUR: N1436

*H. vulgare* L. (Jackson 1915)

*Hordeum* sp. (Pipal 1915)

*Koeleria macrantha* (Ledeb.) Schult. (Poaceae)

*Oryzopsis asperifolia* Michx. (Poaceae) PUR: 62066 - Greenhouse

*Pascopyrum smithii* (Rydb.) Á. Löve (Poaceae)

*Phalaris arundinacea* L. (Poaceae)

*Phleum pratense* L. (Poaceae) (Jackson 1915; Pipal 1915; Anderson and Anderson 1919)

*Poa alpina* L. (Poaceae) PUR: 62060, 62062, N5161, N5262 - Greenhouse

*P. annua* L. PUR: N5263 - Greenhouse

*P. compressa* L. (Underwood 1893; Arthur 1899)

*P. cusickii* Vasey PUR: N5264 - Greenhouse

*P. iridifolia* Hauman PUR: N5265 - Greenhouse

*P. pratensis* L. (Underwood 1893; Arthur 1899; Abbasi et al. 2005)

*P. secunda* J. Presl PUR: 61061, 62067, 62069, N5272 N5280, N5281, N5282, N5283 - Greenhouse

*P. palustris* L. (USDA 1960)

*Pseudoroegneria spicata* (Pursh) Á. Löve (Poaceae)

*Schedonorus pratensis* (Huds.) P. Beauv. (Poaceae); (=*Festuca elatior* L.) (Jackson 1920)

*Secale cereale* L. (Poaceae) (Jackson 1915; Pipal 1915)

*Trisetum spicatum* (L.) K. Richt. (Poaceae) PUR: N5275 - Greenhouse

*Triticum aestivum* L. (Poaceae) (Ruhl et al. 1982; Abbasi et al. 2005); (=*Triticum vulgare* Vill.) (Underwood 1893; Snyder 1898; Arthur 1899; Jackson 1915; Pipal 1915; Anderson and Anderson 1919)

Host not reported (Van Hook 1910)

**Puccinia graminis subsp. graminicola Z. Urb.**

*Poa pratensis* L. (Poaceae)

*Schedonorus arundinaceus* (Schreb.) Dumort., nom. cons. (=*S. phoenix* (Scop.) Holub) (Poaceae)

***Puccinia granulispora* Ellis & Galloway**

*Allium cernuum* Roth (Amaryllidaceae)

***Puccinia grindeliae* Peck**

*Gutierrezia sarothrae* (Pursh) Britton & Rusby (Asteraceae)

*Lygodesmia juncea* (Pursh) D. Don ex Hook. (Asteraceae)

*Solidago canadensis* L. (Asteraceae)

*Puccinia grossulariae* Lagerh.

See: *Puccinia caricina* DC., s.lat.

***Puccinia helianthi* Schwein.**

*Helianthus annuus* L. (Asteraceae) (Underwood 1893; Arthur 1899; Jackson 1915; Anderson and Anderson 1919)

H. cf. hirsutus

*H. decapetalus* L. (Jackson 1920); (=*H. tracheliifolius* Mill.) (Underwood 1893; Arthur 1899; Jackson 1915; Anderson and Anderson 1919)

*H. divaricatus* L. (Underwood 1893; Arthur 1899; Jackson 1915; Anderson and Anderson 1919; Emmons et al. 1960)

*H. giganteus* L. (Jackson 1915)

*H. grosseserratus* M. Martens (Underwood 1893; Snyder 1896; Arthur 1899; Jackson 1915; Anderson and Anderson 1919)

*H. hirsutus* Raf. (Jackson 1915; Anderson and Anderson 1919)

*Helianthus × laetiflorus Pers*. (pro sp.) [*pauciflorus × tuberosus*] (Jackson 1920; Emmons et al. 1960)

*Helianthus × laetiflorus* (=*H. scaberrimus* Elliott) (Jackson 1920)

*H. maximiliani* Schrad. (McCain et al. 1990)

*H. microcephalus* Torr. & A. Gray (McCain and Hennen 1981)

*H. mollis* Lam. (Jackson 1915)

*H. occidentalis* Riddell (Jackson 1915)

*H. parviflorus* Hornem. (Anderson and Anderson 1919)

*H. pauciflorus* Nuttall

*H. petiolaris* Nutt. (Jackson 1915)

*Helianthus* sp. (Underwood 1893)

*H. s strumosus* L. (Underwood 1893; Arthur 1899; Jackson 1915; Anderson and Anderson 1919)

*H. tomentosus* Michx.

*H. tuberosus* L. (Jackson 1915)

*Helianthus × glaucus* Small (pro sp.) [*divaricatus × microcephalus*]

*Heliopsis helianthoides* (L.) Sweet (Asteraceae) (Jackson 1915); var. scabra (Dunal) Fernald (=*Heliopsis scabra* Dunal) (Underwood 1893; Arthur 1899; USDA 1960)

Host not reported (Van Hook 1910)

*Puccinia heliopsidis* Schwein.

See: *Puccinia helianthi* Schwein.

***Puccinia hemerocallidis* Thüm.**

*Hemerocallis* sp. (Liliaceae) PUR: N1322 (Hernández et al. 2002)

*H.* cv. ‘Strawberry Candy’ PUR: 11602

***Puccinia heterospora* Berk. & M.A. Curtis**

*Sida spinosa* L. (Malvaceae)

***Puccinia heucherae* (Schwein.) Dietel**

*Saxifraga pensylvanica* L. (=*Micranthes pensylvanica* (L.) Haw.) (Saxifragaceae) (Jackson 1915)

*Mitella diphylla* L. (Saxifragaceae)

*Puccinia hibisciata* (Schwein.) Kellerm.

See: *Puccinia schedonnardi* Kellerm. & Swingle

***Puccinia hieracii* (Röhl.) H. Mart.**

*Chrysanthemum* sp. (Asteraceae)

*Cnicus lanceolatus* (L.) Willd. (=*Carduus lanceolatus* L.) (Asteraceae) (Underwood 1893; Arthur 1899) [Doubtful host]

*Hieracium canadense* Michx. (Asteraceae) (Jackson 1920)

*H. gronovii* L.

*H. scabrum* Michx. (Jackson 1915; Anderson and Anderson 1919)

*Taraxacum dens-leonis* Desf. (Asteraceae) (Snyder 1898) [Doubtful host]

*T. officinale* F.H. Wigg. (Underwood 1893; Snyder 1896; Anderson and Anderson 1919; Parmelee and Savile 1981); ssp. officinale (=*Leontodon taraxacum* L.) (Jackson 1915)

*T. laevigatum* (Willd.) DC. (=*T. erythrospermum* (Willd.) DC.) (Jackson 1915)

*T. taraxacum* H. Karst. (Arthur 1899)

*Puccinia hieraciata* (Schwein.) H.S. Jacks.

See: *Puccinia dioicae* Magnus, s.lat.

***Puccinia hordei* G.H. Otth**

*Hordeum vulgare* L. (Poaceae) (Jackson 1920)

*Ornithogalum umbellatum* L. (Asparagaceae)

*Puccinia hydnoidea* (Berk. & M.A. Curtis) Arthur

See: *Puccinia dioicae* Magnus, s.lat.

***Puccinia hydrophylli* Peck & Clinton**

*Hydrophyllum virginianum* L. (Hydrophyllaceae)

***Puccinia hyssopi* Schwein.**

*Agastache nepetoides* (L.) Kuntze (Lamiaceae)

***Puccinia impatientis* (Schwein.) Arthur**

*Agrostis perennans* (Walter) Tuck. (Poaceae) (Jackson 1915; Emmons et al. 1960)

*Elymus canadensis* L. (Poaceae) (Jackson 1915)

*E. virginicus* L. (Underwood 1893; Snyder 1896; Arthur 1899; Jackson 1915; Mains 1933); (=*E. striatus* Willd.) (Jackson 1915; Anderson and Anderson 1919)

*E. hystrix* L. (=*Hystrix patula* Moench) (USDA 1960); (=*H. hystrix* (L.) Millsp) (Jackson 1915)

*Impatiens capensis* Meerb. (Balsaminaceae); (=*I. fulva* Nutt.) (Underwood 1893)

*I. pallida* Nutt. (Snyder 1896; Jackson 1915; Van Hook 1928, 1929)

*Impatiens* sp.

*I. capensis* Meerb. (=*I. biflora* Walter) (Jackson 1915; Anderson and Anderson 1919)

*Puccinia indusiata* Dietel & Holw., ined.

See: *Puccinia cyperi* Arthur

*Puccinia interstitialis* Tranzsch.

See: *Gymnoconia peckiana* (Howe) Trotter

***Puccinia interveniens* (Peck) Bethel**

Achnatherum nelsonii subsp. dorei (Barkworth & Maze) Barkworth (Poaceae)

*Sidalcea candida* A. Gray (Malvaceae)

***Puccinia iridis* Wallr.**

*Iris fulva* Ker Gawl. (Iridaceae) (USDA 1960)

*Iris* sp. (USDA 1960)

*I. versicolor* L. (Jackson 1915; Anderson and Anderson 1919)

*Iris × filifolia-xiphium* Anon. (USDA 1960)

*I. xiphioides* Ehrh. (USDA 1960)

*I. xiphium* L. (USDA 1960)

*Puccinia juniperi-virginianae* (Schwein.) Arthur

See: *Gymnosporangium juniperi-virginianae* Schwein.

***Puccinia kansensis* Ellis & Barthol.**

*Buchloe dactyloides* (Nutt.) Engelm. (Poaceae)

*Physalis heterophylla* Nees (Solanaceae) (Jackson 1917)

P. longifolia var. subglabrata (Mack. & Bush) Cronquist

***Puccinia kuhniae* Schwein.**

Brickellia eupatorioides var. eupatorioides (L.) Shinners (=*Kuhnia eupatorioides* L.) (Asteraceae) (Underwood 1893; Snyder 1896; Arthur 1899; Jackson 1915; Jackson 1920; Cummins et al. 1969)

*Kuhnia* sp. (Asteraceae) (Cummins 1978)

**Puccinia laschii var. laschii Lagerh.**

*Arctium minus* (Hill) Bernh. (Asteraceae) [Doubtful host]

*Cirsium* sp. (Asteraceae)

*C. hillii* (Canby) Fernald (=C. pumilum subsp. hillii (Canby) J.W. Moore & Frankton (Savile 1970)

*C. undulatum* (Nutt.) Spreng. [as ‘*undulatus*’] (Jackson 1920)

***Puccinia lateripes* Berk. & Ravenel**

Ruellia caroliniensis subsp. ciliosa var. cinerascens (Fernald) Kartesz & Gandhi (=*R. ciliosa* Pursh) (Acanthaceae) (Jackson 1915; Anderson and Anderson 1919; Laundon 1963)

*R. strepens* L. (Underwood 1893; Arthur 1899; Jackson 1915; Anderson and Anderson 1919; Laundon 1963)

**Puccinia lateripes var. lateripes Berk. & Ravenel**

Ruellia caroliniensis subsp. ciliosa var. cinerascens (Fernald) Kartesz & Gandhi (=*R. ciliosa* Pursh) (Acanthaceae) (Laundon 1963)

**Puccinia lateripes var. strepentis G.F. Laundon**

*Ruellia strepens* L. (Acanthaceae) (Snyder 1896; Laundon 1963)

***Puccinia leptochloae* Arthur & Fromme**

*Talinum paniculatum* (Jacq.) Gaertn. (Portulacaceae)

***Puccinia liatridis* Bethel.**

*Liatris pycnostachya* Michx. (Asteraceae) (USDA 1960)

***Puccinia liliacearum* Duby**

*Ornithogalum umbellatum* L. (Asparagaceae) (Abbasi et al. 2016b)

***Puccinia lithospermi* Ellis & Kellerm.**

*Evolvulus nuttalliana* R & S. (Convolvulaceae)

***Puccinia lobeliae* W.R. Gerard**

*Lobelia siphilitica* L. (Campanulaceae) (Underwood 1893; Snyder 1896; Arthur 1899; Jackson 1915; Anderson and Anderson 1919)

**Puccinia longipes var. brevipes (Dietel) Z. Urb.**

*Vernonia altissima* (Asteraceae) (Arthur 1934)

**Puccinia longipes var. longipes Lagerh.**

*Vernonia fasciculata* Michx. (Asteraceae) (Underwood 1893; Arthur 1899; Jackson 1915; Cash 1952)

*V. gigantea* (Walter) Trel. (Urban 1971)

*Vernonia* sp. (Urban 1971)

*Puccinia ludibunda* Ellis & Everh.

See: *Puccinia dioicae* Magnus, s.lat.

***Puccinia ludovicianae* Fahrend.**

*Artemisia ludoviciana* Nutt. (Asteraceae)

*Puccinia lysimachiata* (Link) Kern

See: Puccinia caricina var. limosae (Magnus) Jørst.

***Puccinia magelhaenica* Peyr.**

*Berberis fendleri* A. Gray (Berberidaceae)

*Koeleria macrantha* (Ledeb.) Schult. (Poaceae)

*Mahonia* sp. (Berberidaceae)

***Puccinia magnusiana* Körn.**

*Anemone canadensis* L. (Ranunculaceae)

*Puccinia majanthae* Arthur & Holw.

See: *Puccinia sessilis* J. Schröt.

***Puccinia malvacearum* Bertero ex Mont.**

*Alcea rosea* L. (Malvaceae) (Jackson 1915); [as ‘*Althaea*’] (Ruhl et al. 1981, 1982, 1983; Pipal 1915; Anderson and Anderson 1919)

*Malva neglecta* cf. Wallr.

*M. neglecta* Wallr. (Malvaceae) (McCain and Hennen 1981)

*M. rotundifolia* L. (Jackson 1915; Lee 1929)

Host not reported (Van Hook 1910)

***Puccinia mariae-wilsoniae* G.W. Clinton [as ‘*mariae-wilsoni***’]

*Claytonia virginica* L. (Portulacaceae) (Jackson 1915; Anderson and Anderson 1919; Arthur 1934)

Host not reported (Van Hook 1934)

***Puccinia marylandica* Lindr.**

*Sanicula canadensis* L. (Apiaceae) (Underwood 1893; Arthur 1899; Jackson 1915; Anderson and Anderson 1919; Jackson 1920; Emmons et al. 1960)

*S. marilandica* L. (USDA 1960)

*Sanicula* sp. (Cooke 1967)

*Puccinia maydis* Carr.

See: *Puccinia sorghi* Schwein.

***Puccinia melampodii* Dietel & Holw.**

*Calendula officinalis* L. (Asteraceae) (USDA 1960)

***Puccinia melicae* P. Syd. & Syd.**

*Melica mutica* Walter (Poaceae) (Jackson 1915)

***Puccinia menthae* Pers.**

*Blephilia ciliata* (L.) Benth. (Lamiaceae) (Jackson 1915; USDA 1960)

*B. hirsuta* (Pursh) Benth. (Underwood 1893; Snyder 1896; Arthur 1899; Jackson 1915; Anderson and Anderson 1919; USDA 1960)

*Cunila origanoides* (L.) Britton (Lamiaceae) (Jackson 1915; Arthur 1899; Emmons et al. 1960); (=*C. mariana* L.) (Underwood 1893)

*Hedeoma pulegioides* (L.) Pers. (Lamiaceae)

*Koellia flexuosa* MacMill. (Lamiaceae) (Jackson 1920)

*Mentha arvensis* L. (=*M. canadensis* L.) (Lamiaceae) (Underwood 1893; Arthur 1899; Jackson 1915; Anderson and Anderson 1919)

*Mentha × piperita* L. (pro sp.) [*aquatica × spicata*]

*M. spicata* L. (Jackson 1915)

*Monarda clinopodia* L. (Lamiaceae)

*M. didyma* L.

*M. fistulosa* L. (Underwood 1893; Snyder 1896; Arthur 1899; Jackson 1915; Anderson and Anderson 1919; Emmons et al. 1960; Cooke 1967)

*M. punctata* L. (Jackson 1920)

*Monarda* sp. (Underwood 1893)

*Pycnanthemum flexuosum* (Walter) Britton, Sterns & Poggenb. (Lamiaceae)

*P. lanceolatum* Pursh (Underwood 1893)

*P. muticum* (Michx.) Pers. (Underwood 1893)

*Pycnanthemum* sp. (Snyder 1896)

*P. virginianum* (L.) T. Dur. & B.D. Jacks. ex B.L. Rob. & Fernald; (=*Koellia virginiana* (L.) MacMill.) (Arthur 1899; Jackson 1915)

P. verticillatum var. pilosum (Nutt.) Cooperr. (=*P. pilosum* Nutt.); (=*Koellia pilosa* (Nutt.) Britton) (Arthur 1899; Jackson 1915)

***Puccinia minutissima* Arthur**

*Decodon verticillatus* (L.) Elliott (Lythraceae) (Jackson 1915; USDA 1960)

*Carex lasiocarpa* Ehrh. (Cyperaceae) (Jackson 1915)

***Puccinia modiolae* P. Syd & Syd.**

*Althaea* sp. (Malvaceae) (Aime and Abbasi 2018)

***Puccinia monoica* Arthur**

*Arabis* sp. (Brassicaceae)

*Koeleria macrantha* (Ledeb.) Schult. (Poaceae)

*Trisetum spicatum* (L.) K. Richt. (Poaceae)

***Puccinia montanensis* Ellis**

*Elymus canadensis* L. (Poaceae) (Jackson 1915; USDA 1960; Cummins and Greene 1966)

*E. hystrix* L.

*Elymus* sp.

*Puccinia muhlenbergiae* Arthur & Holw.

See: *Puccinia schedonnardi* Kellerm. & Swingle

*Puccinia nigrovelata* Ellis & Tracy

See: *Puccinia canaliculata* (Schwein.) Lagerh.

*Puccinia nolitangeris* Corda [reported as ‘*nolitangere*’]

See: *Puccinia argentata* (Schultz) G. Winter

***Puccinia novopanici* Demers, Miao Liu & Hambl.**

*Panicum virgatum* (Poaceae) PUR: N22686

***Puccinia obliqua* Berk. & M.A. Curtis**

Gonolobus suberosus var. granulatus (Scheele) Krings & Q.Y. Xiang (Apocynaceae)

***Puccinia obscura* J. Schröt.**

*Luzula bulbosa* (Alph. Wood) Smyth & Smyth (Juncaceae) (McCain et al. 1990)

*L. campestris* (L.) DC. (=*Juncoides campestre* (L.) Kuntze) (Jackson 1915; Anderson and Anderson 1919)

L. multiflora subsp. frigida (Buchenau) Krecz. (=*L. intermedia* (Thuill.) A. Nelson)

*L. multiflora* (Ehrh.) Lej.

***Puccinia obtecta* Peck**

*Bidens connata* Muhl. ex Willd. (Asteraceae)

*B. frondosa* L. (Jackson 1920; USDA 1960; Savile 1972)

*B. coronata* (L.) Britton (=*B. trichosperma* (Michx.) Britton) (Jackson 1920)

*Schoenoplectus lacustris* (L.) Palla (=*Scirpus lacustris* L.) (Cyperaceae) (Arthur 1899)

*S. pungens* (Vahl) Palla

*S. tabernaemontani* (C.C. Gmel.) Palla (=*Scirpus validus* Vahl) (Jackson 1915; Anderson and Anderson 1919; Savile 1972)

*S. americanus* (Pers.) Volkart ex Schinz & R. Keller (=*Scirpus americanus* Pers.) (Jackson 1915; Savile 1972)

***Puccinia orbicula* Peck & Clinton**

*Prenanthes alba* L. (Asteraceae) (Underwood 1893; Snyder 1896); (=*Nabalus albus* (L.) Hook.) (Arthur 1899; Jackson 1915)

*Prenanthes* sp. (Asteraceae)

***Puccinia pammelii* (Trel.) Arthur**

*Euphorbia corollata* (Kenaley et al. 2018)

*Puccinia panici* Dietel

See: *Puccinia emaculata* Schwein.

*Puccinia patruelis* Arthur

See: *Puccinia dioicae* Magnus, s.lat.

***Puccinia pattersoniae* P. Syd. & Syd.**

*Tripsacum dactyloides* (L.) L. (Poaceae)

*Puccinia peckii* (De Toni) Kellerm.

See: *Puccinia dioicae* Magnus, s.lat.

*Puccinia peckiana* Howe

See: *Gymnoconia peckiana* (Howe) Trotter

***Puccinia persistens* Plowr., s.lat.**

*Actaea racemosa* L. (Ranunculaceae)

*Anemone virginiana* L. (Ranunculaceae)

*Anemonella thalictroides* (L.) Eames & B. Boivin (Ranunculaceae) (USDA 1960)

*Aquilegia canadensis* L. (Ranunculaceae)

*Aquilegia* sp. (Underwood 1893)

**Puccinia persistens subsp. agropyri (Ellis & Everh.) J. Marková & Z. Urb.**

*Bromus kalmii* A. Gray (=*purgans* auct. non L.) (Poaceae) (USDA 1960)

*Clematis drummondii* Torr. & A. Gray (Ranunculaceae)

C. hirsutissima var. scottii (Porter) Erickson

*C. ligusticifolia* Nutt.

*C. virginiana* L.

*Elymus hystrix* L. (=*Hystrix patula* Moench) (Poaceae) (USDA 1960)

**Puccinia persistens subsp. triticina (Erikss.) Z. Urb. & J. Marková**

*Triticum aestivum* L. (Poaceae) (Ruhl et al. 1981, 1982); (=*T. vulgare* Vill.) Poaceae (Jackson 1915; Pipal 1915; Anderson and Anderson 1919)

**Puccinia phragmitis var. phragmitis (Schumach.) Körn.**

*Rumex crispus* L. (Polygonaceae) (Arthur 1902)

*R. obtusifolius* L. (Arthur 1902)

***Puccinia physostegiae* Peck & Clinton**

*Physostegia parviflora* Nutt. ex A. Gray (Lamiaceae)

*P. virginiana* (L.) Benth. (Arthur 1899; Jackson 1915; USDA 1960; Cooke 1967); [reported as ‘*virginica*’] (Snyder 1896)

***Puccinia pimpinellae* (F. Strauss) Link**

*Chaerophyllum procumbens* (L.) Crantz (Apiaceae) (McCain et al. 1990)

*Osmorhiza claytonii* (Michx.) C.B. Clarke (Apiaceae)

*O. longistylis* (Torr.) DC. (Jackson 1920)

***Puccinia poae-nemoralis* G.H. Otth**

*Alopecurus aequalis* Sobol. (Poaceae) (USDA 1960)

*A. geniculatus* L. (Jackson 1915; USDA 1960) PUR: 23412

*Catabrosa aquatica* (L.) P. Beauv. (Poaceae) (USDA 1960)

*Poa pratensis* L. (Poaceae) (McCain et al. 1990)

*Poa* sp.

***Puccinia poarum* Nielsen**

*Festuca* sp. (Poaceae)

*Hymenoxys hoopesii* (A. Gray) Bierner (Asteraceae)

*Koeleria macrantha* (Ledeb.) Schult. (=*nitida* Nutt., nom. utique rej.) (Poaceae) (McCain et al. 1990)

*Liatris punctata* Hook. (Asteraceae)

*L. scariosa* (L.) Willd.

*L. spheroidea* Michx.

*L. spicata* (L.) Willd.

*Poa pratensis* L. (Poaceae) (Snyder 1898; Arthur 1899; Jackson 1915; Pipal 1915; Anderson and Anderson 1919)

*Puccinia poculiformis* (Jacq.) Wettst.

See: *Puccinia graminis* Pers.

*Puccinia podophylli* Schwein.

See: *Allodus podophylli* (Schwein.) Arthur

***Puccinia polemonii* Dietel & Holw.**

*Polemonium reptans* L. (Polemoniaceae) (Jackson 1920; USDA 1960)

***Puccinia polygoni-amphibii* Pers.**

*Geranium maculatum* L. (Geraniaceae) (Jackson 1915)

Polygonum amphibium L. (Polygonaceae); var. emersum Michx. (=*Persicaria amphibia* (L.) Gray & *P. emersum* (Michx.) Britton) (Arthur 1899; Jackson 1915); var. emersum (=*Persicaria muehlenbergii* (S. Watson) Small & *P. muehlenbergii* S. Watson) (Underwood 1893; Jackson 1915; Anderson and Anderson 1919)

*P. convolvulus* L. (Arthur 1899; Anderson and Anderson 1919); (=*Tiniaria convolvulus* (L.) Webb & Moq.) (Jackson 1915)

*P. erectum* L. (Snyder 1896)

*P. hydropiperoides* Michx. (Snyder 1898; Arthur 1899); (=*Persicaria hydropiperoides* (Michx.) Small) (Jackson 1915)

*P. lapathifolium* L.; (=*Persicaria lapathifolia* (L.) Gray) (Jackson 1915)

*P. pensylvanicum* L. (=pensylvanicum var. laevigatum Fernald) (Arthur 1899; McCain et al. 1990); (=*Persicaria pensylvanica* (L.) Small) (Jackson 1915)

*P. punctatum* Elliott (Arthur 1899); var. punctatum (=*P. acre* Kunth) (Underwood 1893; Anderson and Anderson 1919); (=*Persicaria punctata* (Elliott) Small) (Jackson 1915)

*P. scandens* L. (Arthur 1899; Cooke 1967); (=*Tiniaria scandens* (L.) Small) (Jackson 1915)

*Polygonum* sp.

*Polygonum virginianum* L. (Cooke 1967)

*P. amphibium* var. *stipulaceum Coleman* (=*P. natans* L.)

***Puccinia polysora* Underw.**

*Zea mays* L. (Poaceae) (Ullstrup and Lavoilette 1959; Hanlin et al. 1978; Schall et al. 1983)

*Puccinia prenanthis* (Pers.) Fuckel

See: *Puccinia orbicula* Peck & Clinton

*Puccinia pringsheimiana* Kleb.

See: *Puccinia caricina* DC., s.lat.

***Puccinia procera* Dietel & Holway**

*Phacelia distans* Benth. (Hydrophyllaceae)

***Puccinia proserpinacae* Farl.**

*Proserpinaca palustris* L. (Haloragaceae)

*Puccinia pruni-spinosae* Pers., sensu Anderson and Anderson (1919)

See: *Tranzschelia arthurii* Tranzschel & M.A. Litv.

***Puccinia pseudostriiformis* M. Abbasi, Hedjar. & M. Scholler**

*Poa compressa* L. (Poaceae)

*P. nemoralis* L.

*P. pratensis* L.

*P. secunda* J. Presl

***Puccinia punctata* Link**

*Galium asprellum* Michx. (Rubiaceae) (Underwood 1893; Arthur 1899; Jackson 1915)

*G. concinnum* Torr. & A. Gray (Underwood 1893; Arthur 1899; Jackson 1915; Anderson and Anderson 1919; Cooke 1967)

*Galium* sp.

**Puccinia punctata var. troglodytes (Lindr.) Arthur**

*Galium triflorum* Michx. (Rubiaceae) (Underwood 1893; Arthur 1899; Jackson 1915; Anderson and Anderson 1919; Lee 1929; Emmons et al. 1960)

***Puccinia punctiformis* (F. Strauss) Röhl.**

*Cirsium arvense* (L.) Scop. (Asteraceae) (McCain et al. 1990)

***Puccinia purpurea* Cooke**

*Sorghum bicolor* (L.) Moench (Poaceae) (Ullstrup and Lavoilette 1959); subsp. bicolor (L.) Moench (=*S. vulgare* Pers.) (Ullstrup and Lavoilette 1959); subsp. drummondii (Nees ex Steud.) de Wet & Harlan (=*S. sudanense* (Piper) Stapf) (Ullstrup and Lavoilette 1959)

*S. halepense* (L.) Pers. (Ullstrup and Lavoilette 1959)

*Puccinia pustulata* Arthur

See: *Puccinia andropogonis* Schwein.

***Puccinia pygmaea* Erikss.**

*Calamagrostis × acutiflora* (Schrad.) Rchb. (Poaceae)

*Puccinia ranunculi* Seym., Nom. illegit.

See: *Tranzschelia pseudofusca* M. Scholler & M. Abbasi

***Puccinia recondita* Roberge ex Desm. s.str.**

*Anchusa arvensis* (L.) M. Bieb. (Boraginaceae)

*A. capensis* Thunb. PUR: 23972, 65619, 6580 – Greenhouse & Hort. Garden

*A. officinalis* L. (USDA 1960)

*Anchusa* sp. PUR: 65620 – Greenhouse

*Hordeum jubatum* L. (Poaceae) N1436

*Secale cereale* L. (Poaceae) (Snyder 1896; Arthur 1899; Arthur 1909; Jackson 1915; Anderson and Anderson 1919; Pipal 1915; USDA 1960)

*Puccinia recondita* Roberge ex Desm., sensu Cummins 1971, nom. ambig.

See: *Puccinia persistens* Plowr., s.lat.; *P. impatientis* (Schwein.) Arthur; *P. bromina* Erikss. s.lat.

*Avena sativa* L. (Poaceae) (USDA 1960) [Doubtful host]

*Bromus japonicus* Thunb. (Poaceae) (USDA 1960)

*B. kalmii* A. Gray (=*purgans* auct. non L.) (USDA 1960; Cooke 1967)

Notes: records on *Bromus* species probably refer to *Puccinia bromina*

*Elymus canadensis* L. (Poaceae)

*E. hystrix* L. (=*Hystrix patula* Moench) (Emmons et al. 1960; USDA 1960)

*E. repens* (L.) Gould

*E. trachycaulus* (Link) Gould ex Shinners

*E. villosus* Muhl. ex Willd.

*E. virginicus* L.

*Glyceria grandis* S. Watson (Poaceae) (USDA 1960) PUR: 25351

Note: probably refers to Puccinia persistens subsp. agropyri

*Pascopyrum smithii* (Rydb.) A. Löve (Poaceae)

*Ranunculus cymbalaria* Pursh (Ranunculaceae)

Note: Probably refers to *Puccinia eatoniae*

*Thalictrum alpinum* L. (Ranunculaceae)

*T. delavayi* Franch.

*T. dioicum* L.

*T. flavum* L.

*T. glaucum* Sieber ex Lecoy.

*T. pubescens* Pursh

*Thalictrum* sp.

T. delavayi var. delavayi Franch. (=*T. dipterocarpum* Franch.)

Note: records on *Thalictrum* spp. probably refer to *Puccinia cockerelliana* or *P. persistens*.

*Puccinia rhamni* (Pers. ex J.F. Gmel.) Wettst.

See: *Puccinia coronata* Corda, s.lat. and P. coronata var. avenae f. sp. avenae Urban & Marková

*Puccinia rubella* (Pers. ex J.F. Gmel.) Arthur

See: Puccinia phragmitis var. phragmitis (Schumach.) Tul.

*Puccinia rubigo-vera* (DC.) G. Winter, nom. ambig.

This is an ambiguous name used by Underwood (1893) and Snyder (1897)

See: *Puccinia impatientis* (Schwein.) Arthur, *P. recondita* Roberge ex Desm. s.str. and *P. schedonnardi* Kellerm. & Swingle

*Avena sativa* L. (Poaceae) (Underwood 1893) [Doubtful host]

Puccinia rubigo-vera var. agropyrina (Erikss.) Arthur

See: Puccinia persistens subsp. agropyri (Ellis & Everh.) J. Marková & Z. Urb.

Puccinia rubigo-vera var. apocrypta (Ellis & Tracy) Arthur

See: *Puccinia apocrypta* Ellis & Tracy

Puccinia rubigo-vera f. sp. arthuri Mains

See: Puccinia persistens subsp. agropyri (Ellis & Everh.) J. Marková & Z. Urb.

Puccinia rubigo-vera f. sp. impatiensis Mains

See: *Puccinia impatientis* (Schwein.) Arthur

Puccinia rubigo-vera f. sp. indianensis Mains

See: *Puccinia impatientis* (Schwein.) Arthur

Puccinia rubigo-vera f. secalis (Erikss. & Henn.) Mains

See: *Puccinia recondita* Roberge ex Desm. s.str.

*Puccinia ruelliae* (Berk. & Broome) Lagerh.

See: *Puccinia lateripes* Berk. & Ravenel

***Puccinia sambuci* (Schwein.) Arthur**

*Carex aureolensis* Steud. (=*C. frankii* Kunth) (Cyperaceae) (Jackson 1915; Anderson and Anderson 1919; Schoknecht 1981) PUR: 30297

*C. lupuliformis* Sartwell ex Dewey (Jackson 1920)

*C. lupulina* Muhl. ex Willd. (Jackson 1915)

*C. lurida* Wahlenb. (Arthur 1909; Jackson 1915)

*Carex* sp. (Underwood 1893; Snyder 1896; Arthur 1899)

*C. trichocarpa* Muhl. ex Willd. (Jackson 1915; Anderson and Anderson 1919)

*Sambucus racemosa* L. (Caprifoliaceae)

S. nigra subsp. canadensis (L.) R. Bolli (=*S. canadensis* L.) (Underwood 1893; Jackson 1915; Anderson and Anderson 1919; Van Hook 1928)

S. racemosa var. racemosa L. (=*S. pubens* Michx.) (Jackson 1920; USDA 1960)

*Puccinia saniculae* Grev.

See: *Puccinia marylandica* Lindr.

***Puccinia schedonnardi* Kellerm. & Swingle**

*Callirhoe involucrata* (Torr. & A. Gray) A. Gray (Malvaceae)

*Melica mutica* Walter (Poaceae) (Jackson 1920)

*Melica* sp. L. (USDA 1960)

*Muhlenbergia mexicana* (L.) Trin. (Poaceae) (Jackson 1915)

*M. racemosa* (Michx.) Britton, Sterns & Poggenb. (Jackson 1920)

*M. schreberi* J.F. Gmel. (Jackson 1915; Emmons et al. 1960; Cummins and Greene 1961); (as *M. diffusa*) (Underwood 1893) PUR: 21416

*M. sobolifera* (Muhl. ex Willd.) Trin. (Jackson 1920)

*M. sylvatica* (Torr.) Torr. ex A. Gray (=*M. umbrosa* Scribn.) (Snyder 1896; Jackson 1915)

*M. tenuiflora* (Willd.) Britton, Sterns & Poggenb. (Jackson 1915; Anderson and Anderson 1919)

*Napaea dioica* L. (Malvaceae) (Jackson 1915)

*Sphaeralcea angustifolia* (Cav.) G. Don (Malvaceae)

*S. coccinea* (Nutt.) Rydb.

*S. incana* Torr. ex A. Gray

*Triodia seslerioides* Benth. ex Vasey (Poaceae) (Snyder 1896)

*Puccinia schroeteriana* Plowr. & Magnus

See: *Puccinia dioicae* Magnus, s.lat.

***Puccinia sessilis* J. Schröt.**

*Iris versicolor* L. (Iridaceae) (Jackson 1920; USDA 1960)

*Phalaris arundinacea* L. (Poaceae) (Jackson 1915)

***Puccinia seymeriae* Burrill**

*Dasistoma macrophylla* (Nutt.) Raf. (=*Afzelia macrophylla* (Nutt.) Kuntze) (Orobanchaceae) (Jackson 1915)

***Puccinia seymouriana* Arthur**

*Apocynum cannabinum* L. (Apocynaceae) (Jackson 1920; USDA 1960)

*Asclepias syriaca* L. (Apocynaceae)

*Cephalanthus occidentalis* L. (Rubiaceae) (Jackson 1915)

*Spartina pectinata* Bosc ex Link (Poaceae); (=*S. michauxiana* Hitchc.) (Jackson 1915)

*S. cynosuroides* (L.) Roth (Arthur 1909)

***Puccinia silphii* Schwein.**

*Silphium integrifolium* Michx. (Asteraceae) (Jackson 1915)

*S. perfoliatum* L. (Jackson 1915; Anderson and Anderson 1919; Van Hook 1923)

*Silphium* sp. (Underwood 1893; Arthur 1899; Jackson 1915)

*S. terebinthinaceum* Jacq.

***Puccinia smilacis* Schwein.**

*Apocynum cannabinum* L. (Apocynaceae)

*Smilax bona-nox* L. (Smilacaceae) (Jackson 1920)

*S. glauca* Walter (Jackson 1915)

*S. rotundifolia* L.

*S. tamnoides* L. (=*hispida* Muhl. ex Torr.) (Emmons et al. 1960)

***Puccinia sorghi* Schwein.**

*Oxalis corniculata* L. (Oxalidaceae) (Anderson and Anderson 1919; USDA 1960)

*O. stricta* L. (USDA 1960); (=*Xanthoxalis cymosa* (Small) Small) (Jackson 1915)

*Zea mays* L. (Poaceae) (Underwood 1893; Snyder 1898; Arthur 1899; Jackson 1915; Pipal 1915; Anderson and Anderson 1919; Ullstrup and Lavoilette 1959; Hanlin et al. 1978; Ruhl et al. 1980, 1981, 1982)

*Zea mexicana* (Schrad.) Kuntze (Poaceae)

Host not reported (Van Hook 1910)

***Puccinia sparganioidis Ellis* & Barthol.**

*Fraxinus americana* L. (Oleaceae) (McCain and Hennen 1981)

*F. pennsylvanica* Marshall (=*F. lanceolata* Borkh.) (Arthur 1902)

*F. profunda* (Bush) Bush (Jackson 1920)

*Fraxinus* sp. (Ruhl et al. 1981)

*F. viridis* F. Michx. (Van Hook 1927)

*Spartina pectinata* Bosc ex Link (Poaceae); (=*S. michauxiana* Hitchc.) (Jackson 1915)

***Puccinia splendens* Vize**

*Hymenoclea monogyra* Torr. & A. Gray (Asteraceae)

***Puccinia sporoboli* Arthur**

*Allium cernuum* Roth (Amaryllidaceae)

*A. drummondii* Regel

*Lilium philadelphicum* L. (Liliaceae)

*Sporobolus cryptandrus* (Torr.) A. Gray (Poaceae) (Snyder 1896)

**Puccinia sporoboli var. robusta Cummins & H.C. Greene**

*Calamovilfa longifolia* (Hook.) Scribn. (Poaceae) (Cummins and Greene 1961)

*Maianthemum racemosum* (L.) Link (Asparagaceae)

*M. stellatum* (L.) Link (=*Smilacina stellata* (L.) Desf.) (Cummins and Greene 1961)

*Yucca glauca* Nutt. (Agavaceae)

***Puccinia stipae* Arthur**

*Grindelia squarrosa* (Pursh) Dunal (Asteraceae)

*Gutierrezia sarothrae* (Pursh) Britton & Rusby (Asteraceae)

*Hesperostipa spartea* (Trin.) Barkworth (Poaceae)

*Senecio spartioides* Torr. & Gray (Asteraceae)

*Symphyotrichum ericoides* (L.) G.L. Nesom (Asteraceae)

*S. novae-angliae* (L.) G.L. Nesom

*S. porteri* (A. Gray) G.L. Nesom

***Puccinia striiformis* Westend.**

*Triticum aestivum* L. (Poaceae) (McCain and Hennen 1981; Ruhl et al. 1983; Abbasi et al. 2005)

Also see: ‘*Puccinia pseudostriiformis* M. Abbasi, Hedjar. & M. Scholler’ for reports on *Poa* spp.

***Puccinia subnitens* Dietel**

*Abronia fragrans* Nutt. ex Hook. (Nyctaginaceae)

*Atriplex prostrata* Bouchér ex DC. (=*A. hastata* L.) (Chenopodiaceae)

*Capsella bursa-pastoris* (L.) Medik. (Brassicaceae)

*Chenopodium album* L. (Chenopodiaceae) (USDA 1960)

*Cleome hassleriana* Chod. (=*C. spinosa* auct. non Jacq.) (Capparidaceae) (USDA 1960)

*C. serrulata* Pursh (USDA 1960)

Descurainia pinnata var. brachycarpa (Richardson) Detling (Brassicaceae)

*Erysimum capitatum* (Dougl. ex Hook.) Greene (Brassicaceae)

*Lepidium densiflorum* Schrad. (Brassicaceae)

*Lepidium* sp. (USDA 1960)

*L. virginicum* L.

*Polygonum aviculare* L. (Polygonaceae)

*Sarcobatus vermiculatus* (Hook.) Torr. (Chenopodiaceae)

***Puccinia substerilis* Ellis & Everh.**

*Nassella viridula* (Trin.) Barkworth (Poaceae)

***Puccinia substriata* Ellis & Barthol.**

*Paspalum setaceum* Michx. (=P. setaceum var. muhlenbergia (Nash) D.J. Banks) (Poaceae)

*Solanum carolinense* L. (Solanaceae)

**Puccinia substriata var. imposita (Arthur) Ramachar & Cummins**

*Solanum carolinense* L. (Solanaceae)

*S. melongena* L.

*Puccinia tanaceti* DC., sensu Underwood (1893) [also followed by Snyder (1897)]

See: *Puccinia helianthi* Schwein.

**Puccinia tanaceti var. tanaceti DC.**

*Chrysanthemum × morifolium* Ramat. (pro sp.) (Asteraceae)

C. morifolium (Ramat.) Hemsl. var. sinense (Sabine) Makino (=*C. sinense* Sabine) (Jackson 1915)

*Chrysanthemum* sp. (Pipal 1915)

**Puccinia tanaceti var. dracunculina (Fahrend.) Cummins**

*Artemisia* (=*Dracunculoides* L.) *dracunculus* L. (Asteraceae)

*Puccinia taraxaci* Plowr.

See: *Puccinia hieracii* (Röhl.) H. Mart.

***Puccinia tenuis* (Schwein.) Burrill**

Ageratina altissima var. altissima (L.) R.M. King & H. Rob. (=*Eupatorium rugosum* Houtt.) (Asteraceae) (Emmons et al. 1960); var. roanensis (Small) Clewell & Woot. (=*E. urticifolium* Reichard) (Jackson 1915)

*Ageratum altissimum* (=*E. ageratoides* L.f.) (Asteraceae) (Underwood 1893; Snyder 1896; Arthur 1899)

*Puccinia thalictri* Chevall.

See: *Tranzschelia thalictri* (Chev.) Dietel

***Puccinia thelypodii* Cummins**

*Guillenia lasiophylla* (Hook. & Arn.) Greene (=*Thelypodium lasiophyllum* (Hook. & Arn.) Greene) (Brassicaceae)

*Puccinia thompsonii* Hume

See: *Puccinia sambuci* (Schwein.) Arthur

***Puccinia tomipara* Trel.**

*Bromus ciliatus* L. (Poaceae) (Anderson and Anderson 1919)

*Thalictrum thalictroides* (L.) Eames & B. Boivin (=*Anemonella thalictroides* (L.) Spach) (Ranunculaceae) (Anderson and Anderson 1919)

*Puccinia triodiae* Ellis & Barthol.

See: *Puccinia schedonnardi* Kellerm. & Swingle

***Puccinia tripsaci* Dietel & Holw.**

*Ceanothus americanus* L. (Rhamnaceae)

*Tripsacum* sp. (Poaceae) (USDA 1960)

*Puccinia tritici* auct. non Oerst.

See: Puccinia persistens subsp. triticina (Erikss.) Z. Urb. & J. Marková

*Puccinia triticina* Erikss.

See: Puccinia persistens subsp. triticina (Erikss.) Z. Urb. & J. Marková

*Puccinia troglodytes* Lindr. [as ‘*triglodytes*’ by Lee (1929)]

See: Puccinia punctata var. troglodytes (Lindr.) Arthur

***Puccinia tumidipes* Peck**

*Lycium halimifolium* Mill. (= *L. barbarum* L.) (Solanaceae) (Otálora and Berndt 2018)

*Puccinia urticae* Lagerh.

See: *Puccinia urticata* F. Kern.

***Puccinia urticata* F. Kern**

*Carex bullata* Schkuhr ex Willd. (Cyperaceae) (Arthur 1899)

*C. emoryi* Dewey (Jackson 1920)

*C. frankii* Kunth (Arthur 1899)

*C. lacustris* Willd. (Jackson 1915)

*C. lurida* Wahlenb. (Arthur 1899)

*C. pensylvanica* Lam. (Arthur 1899)

*C. siccata* Dewey (=*C. foenea* Willd.) (Arthur 1899)

*C. stipata* Muhl. ex Willd.

*C. straminea* Willd. ex Schkuhr (Arthur 1899)

*C. stricta* Lam. (Jackson 1915) PUR: 66442

*C. trichocarpa* Muhl. ex Willd. (Jackson 1920)

*C. virescens* Muhl. ex Willd. (Arthur 1899)

*Dulichium arundinaceum* (L.) Britton (Arthur 1899)

Urtica dioica L. ssp. gracilis (Aiton) Seland. (=*U. gracilis* Aiton) (Urticaceae) (Arthur 1899; Jackson 1915)

*Puccinia verbenicola* Arthur

See: *Puccinia vilfae* Arthur & Holw.

***Puccinia verbesinae* Schwein.**

*Verbesina occidentalis* (L.) Walter (Asteraceae) (USDA 1960)

*Puccinia vernoniae* Schwein.

See: Puccinia longipes var. brevipes (Dietel) Z. Urb.

Puccinia vernoniae var. caulicola Ellis & Everh.

See: Puccinia longipes var. longipes Lagerh.

***Puccinia veronicarum* DC., s.lat.**

*Veronicastrum virginicum* (L.) Farw. (Plantaginaceae) PUR: N6188

*Puccinia verrucosa* Link

See: *Puccinia glechomatis* DC.

***Puccinia vexans* Farl.**

*Bouteloua curtipendula* (Michx.) Torr. (Poaceae); (=*Atheropogon curtipendulus* (Michx.) Fourn.) (Jackson 1915; Jackson 1920)

*Fouquieria splendens* Engelm. (Fouquieriaceae) PUR: N5514 - Greenhouse

***Puccinia vilfae* Arthur & Holw.**

*Sporobolus compositus* (Poir.) Merr. (Poaceae); (=*S. asper* (P. Beauv.) Kunth) (Arthur 1899; Jackson 1915; Cummins and Greene 1961)

*Verbena hastata* L. (Verbenaceae) (Jackson 1920)

*V. stricta* Vent. (Snyder 1896; Jackson 1915)

*V. urticifolia* L.

*Verbena × illicita* Moldenke [*stricta × urticifolia*] (McCain et al. 1990)

***Puccinia violae* (Schumach.) DC.**

*Viola blanda* Willd. (Violaceae) (Anderson and Anderson 1919); var. palustriformis A. Gray (=V. incognita var. forbesii Brainerd)

*V. cucullata* Aiton (=*V. obliqua* Hill) (Arthur 1899; Arthur and Holway 1901; Anderson and Anderson 1919)

*V. eriocarpa* Schwein. (Jackson 1915)

*V. missouriensis* Greene (Jackson 1920)

V. palmata var. cucullata A. Gray (Underwood 1893)

*V. pubescens* Aiton (Arthur and Holway 1901; Jackson 1915; Anderson and Anderson 1919)

*V. sororia* Willd.; (=*V. papilionacea* Pursh p.p.) (Jackson 1915; Anderson and Anderson 1919)

*Viola* sp. (Underwood 1893; Cooke 1967; Ruhl et al. 1981; Schoknecht 1981)

*V. striata* Aiton (Underwood 1893; Arthur 1899; Arthur and Holway 1901; Jackson 1915; Anderson and Anderson 1919)

Host not reported (Van Hook 1910) [reported as *P. violaceae*]

***Puccinia virgata* Ellis & Everh.**

Sorghum bicolor subsp. bicolor (L.) Moench (=*S. vulgare* Pers.) (Poaceae) PUR: N7456

*Puccinia vulpinoideae* Dietel & Holw. [as’*vulpinoidis*’]

See: *Puccinia dioicae* Magnus, s.lat.

*Puccinia windsoriae* Burrill

See: *Puccinia schedonnardi* Kellerm. & Swingle

***Puccinia windsoriae* Schwein.**

*Ptelea perfoliatum* (Rutaceae) (Snyder 1896) [Doubtful host]

*P. trifoliata* L. (Underwood 1893; Jackson 1915; Anderson and Anderson 1919)

*Sieglingia seslerioides* Scribn. (Poaceae) (Arthur 1899)

*Tridens flavus* (L.) Hitchc. (=*Triodia flava* (L.) Hitchc.) (Poaceae) (Anderson and Anderson 1919; Cooke 1967, 1975); [as ‘*flava*’] (Jackson 1915)

***Puccinia xanthii* Schwein.**

*Ambrosia* sp. (Asteraceae)

*A. trifida* L. (Asteraceae) (Underwood 1893; Snyder 1896; Arthur 1899; Jackson 1915)

*Xanthium* sp. (Asteraceae)

*X. spinosum* L. (Jackson 1915; USDA 1960; McCain et al. 1990)

*X. strumarium* L. (Underwood 1893; Arthur 1899); var. canadense (Mill.) Torr. & A. Gray (McCain et al. 1990); var. canadense (=*X. commune* Britton [as ‘*communis*’]) (Jackson 1915); var. canadense (=*X. canadense* Mill.) (Underwood 1893; Snyder 1896; Arthur 1899; Anderson and Anderson 1919); var. canadense (=*X. pensylvanicum* Wallr.) (Jackson 1915); var. canadense (Mill.) Torr. & A. Gray (=*X. italicum* Moretti); var. glabratum (DC.) Cronquist (McCain et al. 1990); var. glabratum (DC.) Cronquist (=*X. americanum* Walter) (Jackson 1915)

Host not reported (Van Hook 1910)

**Puccinia xanthii f. sp. ambrosiae-trifidae S.W.T. Batra [as ‘ *ambrosia-trifidae*** ’]

*Ambrosia trifida* L. (Asteraceae) (Batra 1981)

***Puccinia* sp.**

*Poa pratensis* L. (Poaceae) (Ruhl et al. 1981)

***Pucciniastrum agrimoniae* (Dietel) Tranzschel**

*Agrimonia eupatoria* L. (Rosaceae) (Underwood 1893; Snyder 1896)

*A. gryposepala* Wallr. (Anderson and Anderson 1919; Emmons et al. 1960)

*A. hirsuta* Bickn. (Arthur 1899; Jackson 1915)

*A. microcarpa* Wallr. (Hiratsuka 1958)

*A. parviflora* Aiton (Underwood 1893; Arthur 1899; Jackson 1915; Hiratsuka 1958)

*A. pubescens* Wallr. (Cooke 1975); (=*A. mollis* (Torr. & A. Gray) Britton) (Jackson 1915)

*A. rostellata* Wallr. (Jackson 1920; Hiratsuka 1958)

*Agrimonia* sp.

Host not reported (Cooke 1967)

*Pucciniastrum americanum* (Farl.) Arthur

See: *Thekopsora americana* (Farl.) Aime & McTaggart

*Pucciniastrum goeppertianum* (Kuehn) Kleb.

See: *Calyptospora columnaris* (Alb. & Schwein.) J.G. Kühn

***Pucciniastrum hydrangeae* (Magnus) Arthur**

*Hydrangea arborescens* L. (Hydrangeaceae) (Underwood 1893;; Snyder 1896; Arthur 1899; Jackson 1915; Anderson and Anderson 1919; Cooke 1967, 1975; Schoknecht 1981)

*Hydrangea* sp.

*H. cinerea* Small (=H. arborescens subsp. discolor (Ser. ex DC.) McClint.) (McCain et al. 1990)

*H. paniculata* Siebold (USDA 1960)

*Tsuga canadensis* (L.) Carriere (Pinaceae) (USDA 1960)

*Pucciniastrum myrtilli* Arthur

See: *Naohidemyces vaccinii* (Jørst.) S. Sato, Katsuya & Y. Hirats. ex Vanderweyen & Fraiture

*Pucciniastrum vaccinii* (G. Winter) Jørst. 1952

See: *Naohidemyces vaccinii* (Jørst.) S. Sato, Katsuya & Y. Hirats. ex Vanderweyen & Fraiture

***Pucciniosira tuberculata* (Ellis & Kellerm.) Buriticá & J.F. Hennen**

*Callirhoe involucrata* (Torr. & A. Gray) A. Gray (Malvaceae) (USDA 1960)

***Ravenelia epiphylla* (Schwein) Dietel**

*Tephrosia virginiana* (L.) Pers. (=T. virginiana var. holosericea (Nutt.) Torr. & A. Gray) (Fabaceae); (=*Cracca virginiana* L.) (Jackson 1915)

*Roestelia lacerata* (Sowerby) Fr.

See: *Gymnosporangium globosum* Farl.

*Roestelia pyrate* Thaxt.

See: *Gymnosporangium juniperi-virginianae* Schwein.

*Teleutospora rudbeckiae* (Arthur & Holw.) Arthur & Bisby

See: *Uromyces rudbeckiae* Arthur & Holw.

***Thekopsora americana* (Farl.) Aime & McTaggart**

*Rubus idaeus* L. (Rosaceae)

***Tranzschelia arthurii* Tranzschel & M.A. Litv.**

Hepatica nobilis var. acuta (Pursh) Steyerm. (Ranunculaceae); var. acuta (=*H. acutiloba* DC.) (Underwood 1893; Jackson 1915; Anderson and Anderson 1919; Van Hook 1934); var. obtusa (Pursh) Steyerm. (=*H. triloba* Chaix) (Anderson and Anderson 1919)

*Prunus serotina* Ehrh. (Rosaceae) (Jackson 1920; Lopez-Franco and Hennen 1990); var. serotina Ehrh. (Lopez-Franco and Hennen 1990)

*P. virginiana* L. (Rosaceae) (Lopez-Franco and Hennen 1990)

*Tranzschelia fusca* (Pers.) Dietel

See: *Tranzschelia pseudofusca* M. Scholler & M. Abbasi

***Tranzschelia pruni-spinosae* (Pers.) Dietel**

*Anemone quinquefolia* L. (Ranunculaceae) (Jackson 1920; Lopez-Franco and Hennen 1990)

*Thalictrum dioicum* L. (Ranunculaceae) (USDA 1960)

**Tranzschelia pruni-spinosae var. americana López-Franco & Hennen**

*Prunus americana* Marshall (Rosaceae)

***Tranzschelia pseudofusca* M. Scholler & M. Abbasi**

*Anemone quinquefolia* L. (Ranunculaceae) (Underwood 1893; Arthur 1899; Jackson 1915; Jackson 1920; Lopez-Franco and Hennen 1990)

*Ranunculus repens* L. (Ranunculaceae) (Underwood 1893)

*Tranzschelia punctata* Arthur

Note: This name as applied could refer to *Tranzschelia arthurii* Tranzschel & M.A. Litv., *T. pruni-spinosae* (Pers.) Dietel or *Aecidium dakotense* Griffiths

***Tranzschelia thalictri* (Chev.) Dietel**

*Thalictrum dioicum* L. (Ranunculaceae) (Underwood 1893; Arthur 1899; Jackson 1915; Anderson and Anderson 1919; Lopez-Franco and Hennen 1990)

***Triphragmium ulmariae* (DC.) Link**

*Filipendula rubra* (Hill.) B.L. Rob. (=*Spiraea rubra* (Hill) Britton) (Rosaceae) (Jackson 1915; Jackson 1920; Arthur 1934; Monoson 1975)

*F. ulmaria* (L.) Maxim. (USDA 1960)

*Ulmaria* sp. (Rosaceae) (Monoson 1975)

***Uredinopsis americana* Syd. & P. Syd.**

*Onoclea sensibilis* L. (Onocleaceae) (Jackson 1917; Hiratsuka 1958)

***Uredinopsis arthurii* Faull**

*Woodwardia virginica* (L.) Sm. (Blechnaceae) (USDA 1960)

***Uredinopsis atkinsonii* Magnus**

*Cystopteris bulbifera* (L.) Bernh. (=*Filix bulbifera* (L.) Underw.) (Cystopteridaceae) (Jackson 1920)

Thelypteris palustris var. pubescens (G. Lawson) Fernald (=Dryopteris thelypteris var. pubescens (G. Lawson) A.R. Prince ex Weath.) (Thelypteridaceae) (Hiratsuka 1958); (=*D. thelypteris* (L.) A. Gray) (Jackson 1917)

Host not reported (Van Hook 1934)

***Uredinopsis ceratophora* Faull**

*Cystopteris bulbifera* (L.) Bernh. (=*Filix bulbifera* (L.) Underw.) (Cystopteridaceae) (USDA 1960)

*Uredinopsis mirabilis* (Peck) Magnus

See: *Uredinopsis americana* Syd. & P. Syd.

***Uredinopsis osmundae* Magnus**

*Abies balsamea* (L.) Mill. (Pinaceae)

*Osmunda claytoniana* L. (Osmundaceae)

***Uredinopsis pteridis* Dietel & Holw.**

*Pteridium aquilinum* (Dennstaedtiaceae) [as *Pteris aquilina* L. (Pteridaceae)] (Lee 1929)

***Uredinopsis struthiopteridis* F.C.M. Störmer**

Athyrium filix-foemina var. cyclosorum Rupr. (Dryopteridaceae)

*Cystopteris bulbifera* (L.) Bernh. (=*Filix bulbifera* (L.) Underw.) (Cystopteridaceae)

*Thelypteris palustris* Schott (Thelypteridaceae)

*Woodwardia virginica* (L.) Sm. (Blechnaceae)

*W. virginica* (=*Anchistea virginica* (L.) C. Presl) (Jackson 1920)

*Uredo bigelowii* (Thüm.) Arthur

See: *Melampsora paradoxa* Dietel & Holw.

*Uredo hydrangeae* Berk. & M.A. Curtis

See: *Pucciniastrum hydrangeae* (Magnus) Arthur

*Uredo polypodii* (Pers.) DC.

See: *Hyalopsora polypodii* (Pers.) Magnus

*Uredo similis* Ellis

See: *Puccinia globosipes* Peck

***Uromyces acuminatus* Arthur**

*Collomia linearis* Nutt. (Polemoniaceae)

*Lysimachia ciliata* L. (Primulaceae)

*Maianthemum stellatum* (L.) Link (=*Smilacina stellata* (L.) Desf.) (Asparagaceae) (McCain et al. 1990)

*Phlox divaricata* L. (Polemoniaceae)

*P. maculata* L. (Jackson 1920)

*Polygonatum biflorum* (Walter) Elliott (Asparagaceae) (Jackson 1917)

*Polemonium reptans* L. (Polemoniaceae) (Jackson 1915; USDA 1960)

*Smilacina* sp. (Liliaceae)

*Spartina pectinata* Bosc ex Link (Poaceae); (=*S. michauxiana* Hitchc.) (Jackson 1915)

*S. pectinata* Link

*Spartina* sp.

Uromyces acuminatus var. polemonii (Arthur) Davis

See: *Uromyces acuminatus* Arthur

***Uromyces americanus* Speg.**

*Schoenoplectus tabernaemontani* (C.C. Gmelin) Palla (=*Scirpus validus* Vahl) (Cyperaceae) (USDA 1960)

***Uromyces amphidymus* Syd. & P. Syd.**

*Glyceria septentrionalis* Hitchc. (Poaceae) (USDA 1960); (=*Panicularia septentrionalis* (Hitchc.) E.P. Bicknell) (Jackson 1915)

***Uromyces andropogonis* Tracy**

Andropogon gyrans Ashe var. gyrans (=*elliottii* Chapm.) (Poaceae) (Hennen 1965)

*A. virginicus* L. (Hennen 1965)

*Viola cucullata* Aiton (=*V. obliqua* Hill) (Violaceae) (Arthur and Holway 1901)

*V. affinis* Le Conte

**Uromyces appendiculatus var. appendiculatus (Pers.) Unger**

*Phaseolus diversifolius* Pers. (Fabaceae) (Snyder 1896)

*Phaseolus* sp. (Underwood 1893)

*P. vulgaris* L. (Jackson 1915; Pipal 1915; Anderson and Anderson 1919; Ruhl et al. 1981, 1982)

*Strophostyles angulosa* Elliott (Fabaceae) (Underwood 1893)

*S. helvola* (L.) Elliott (Arthur 1899; Jackson 1915; Anderson and Anderson 1919)

*S. leiosperma* (Torr. & A. Gray) Piper (=*S. pauciflora* (Benth.) S. Watson) (Jackson 1915)

*S. umbellata* (Muhl. ex Willd.) Britton (Jackson 1915)

*Vigna angularis* (Willd.) Ohwi & Ohashi (=*P. angularis* (Willd.) W. Wight) (Fabaceae) (USDA 1960) [Doubtful host]

Host not reported (Van Hook 1910)

***Uromyces ari-triphylli* (Schwein.) Seeler**

*Arisaema dracontium* (L.) Schott (Araceae) (Underwood 1893; Snyder 1896; Arthur 1899; Jackson 1915; Anderson and Anderson 1919)

*A. triphyllum* (L.) Schott (Underwood 1893; Snyder 1896, 1898; Arthur 1899; Jackson 1915; Anderson and Anderson 1919; Cooke 1967, 1975)

*Peltandra virginica* (L.) Schott (Araceae) (Jackson 1915; USDA 1960)

Host not reported (Van Hook 1910)

***Uromyces asclepiadis* Cooke**

*Asclepias cornuti* Decne. (Apocynaceae) (Underwood 1893; Snyder 1896, 1898)

*A. incarnata* L. (Jackson 1915, 51; Snyder 1896; Arthur 1899; Anderson and Anderson 1919)

*A. purpurascens* L. (Underwood 1893; Arthur 1899; Jackson 1915; Anderson and Anderson 1919)

*A. syriaca* L. (Arthur 1899; Jackson 1915; Anderson and Anderson 1919)

*A. tuberosa* L.

*Matelea obliqua* (Jacq.) Woodson (Asclepiadaceae); (=*Vincetoxicum shortii* (A. Gray) Britton) (Jackson 1915)

Host not reported (Van Hook 1910)

*Uromyces caladii* Farl.

See: *Uromyces ari-triphylli* (Schwein.) Seeler

*Uromyces caryophyllinus* (Schrank) J. Schröt.

See: *Uromyces dianthi* (Pers.) Niessl

***Uromyces coloradensis* Ellis & Everh.**

*Vicia americana* Muhl. ex Willd. (Fabaceae) (Jackson 1920)

***Uromyces dactylidis* G.H. Otth**

*Poa pratensis* L. (Poaceae) (Jackson 1915; USDA 1960)

***Uromyces dianthi* (Pers.) Niessl**

*Dianthus caryophyllus* L. (Caryophyllaceae) (Arthur 1899; Jackson 1915; Pipal 1915; Anderson and Anderson 1919)

*Dianthus* spp. (Underwood 1893; Van Hook 1912)

***Uromyces dictyospermae* Ellis & Everh. ex Tranzschel**

*Euphorbia spathulata* Lam. (Euphorbiaceae)

***Uromyces elegans* (Berk. & M.A. Curtis) Lagerh.**

*Trifolium carolinianum* Michx. (Fabaceae)

***Uromyces eragrostidis* Tracy**

*Echeandia flavescens* (Schult. & Schult. f.) Cruden (=*Anthericum torreyi* Baker) (Asparagaceae)

*Uromyces euphorbiae* Cooke & Peck

See: *Uromyces proëminens* (DC.) Lév.

*Uromyces fabae* (Pers.) de Bary

See: *Uromyces viciae-fabae* (Pers.) J. Schröt.

*Uromyces fallens* (Desm.) Barthol.

See: Uromyces trifolii-repentis var. fallens (Desm.) Arthur

*Uromyces gaurinus* (Peck) Snyder [as ‘*gaurina*’]

See: *Uromyces plumbarius* Peck

***Uromyces geranii* (DC.) Lév.**

*Geranium maculatum* L. (Geraniaceae) (Underwood 1893; Snyder 1896, 1898)

*Uromyces graminicola* Burrill

See: *Puccinia graminicola* (Burrill) Demers & Castl.

***Uromyces halstedii* De Toni**

*Leersia oryzoides* (L.) Sw. (Poaceae) (USDA 1960)

*Trillium* sp. (Melanthiaceae) (Snyder 1896; Jackson 1917)

***Uromyces hedysari-paniculati* (Schwein.) Farl.**

*Desmodium canadense* (L.) DC. (=*Meibomia canadensis* (L.) Kuntze) (Fabaceae) (Snyder 1896; Arthur 1899)

*D. canescens* (L.) DC. (Underwood 1893; Anderson and Anderson 1919); (=*Meibomia canescens* (L.) Kuntze) (Arthur 1899; Jackson 1915)

*D. ciliare* (Muhl. ex Willd.) DC. (McCain et al. 1990)

D. cuspidatum (Muhl. ex Willd.) DC. ex D. Don var. cuspidatum (=*M. bracteosa* (Michx.) Kuntze) (Jackson 1915); var. longifolium (Torr. & A. Gray) B.G. Schub. (McCain et al. 1990)

*D. glabellum* (Michx.) DC. (=*D. dillenii* Darl.) (Anderson and Anderson 1919)

*D. laevigatum* (Nutt.) DC. (=*M. laevigata* (Nutt.) Kuntze) (Underwood 1893; Arthur 1899; Jackson 1915; Anderson and Anderson 1919)

*D. paniculatum* (L.) DC. (Underwood 1893; Anderson and Anderson 1919); var. paniculatum (=*M. paniculata* (L.) Kuntze) (Arthur 1899; Jackson 1915)

*D. perplexum* B.G. Schub.; (=*M. dillenii* (Darl.) Kuntze) (Snyder 1896; Arthur 1899; Jackson 1915); (=*D. dillenii* Darl.) (Underwood 1893)

*D. rotundifolium* DC. (Emmons et al. 1960); (=*M. michauxii* Vail) (Jackson 1920)

*D. sessilifolium* (Torr.) Torr. & A. Gray (McCain et al. 1990)

*Desmodium* sp. (Underwood 1893; Cooke 1967, 1975)

*D. viridiflorum* (L.) DC. (Underwood 1893)

*D. viridiflorum* (L.) DC. (=*M. viridiflora* (L.) Kuntze) (Arthur 1899; Jackson 1915)

*Uromyces hordei* Tracy

See: *Uromyces hordeinus* (Arthur) Barthol.

***Uromyces hordeinus* (Arthur) Barthol.**

*Hordeum pusillum* Nutt. (Poaceae) (Jackson 1920)

*Nothoscordum bivalve* (L.) Britton (Amaryllidaceae)

***Uromyces houstoniatus* (Schwein.) J. Sheld.**

*Houstonia caerulea* L. (Rubiaceae) (Jackson 1917)

*H. longifolia* Gaertn.

*Sisyrinchium angustifolium* Mill. (=*S. gramineum* Curtis) (Iridaceae)

*Uromyces howei* Peck

See: *Uromyces asclepiadis* Cooke

*Uromyces hyperici* M.A. Curtis

See: *Uromyces triquetrus* Cooke

*Uromyces hyperici-frondosi* (Schwein.) Arthur

See: *Uromyces triquetrus* Cooke

***Uromyces junci* (Desm.) Tul. & C. Tul.**

*Ambrosia artemisiifolia* Bess. (Asteraceae)

*Ambrosia psilostachya* DC. (Asteraceae)

*Cirsium flodmanii* Arthur (Asteraceae)

*Juncus tenuis* Willd. (Juncaceae) (Snyder 1896, 1898; Arthur 1899)

***Uromyces junci* - *effusi* P. Syd. & Syd.**

*Juncus effusus* L. (Juncaceae) (McCain et al. 1990)

*Uromyces lespedezae* Peck

See: *Uromyces lespedezae-procumbentis* (Schwein.) M.A. Curtis

***Uromyces lespedezae-procumbentis* (Schwein.) M.A. Curtis**

*Lespedeza capitata* Michx. (Fabaceae) (Jackson 1915)

*L. frutescens* (L.) Hornem. (Arthur 1899; Jackson 1915; Anderson and Anderson 1919); (=*L. intermedia* (S. Watson) Britton, nom. inq.) (McCain et al. 1990)

*L. hirta* (L.) Hornem. (Jackson 1915)

*L. procumbens* Michx. (Underwood 1893; Arthur 1899; Jackson 1915; Anderson and Anderson 1919)

*L. repens* (L.) W.P.C. Barton (Snyder 1896; Arthur 1899; Jackson 1915; McCain et al. 1990)

*L. reticulata* Pers. (Underwood 1893)

*Lespedeza* sp. (Underwood 1893; Cooke 1975, 1967)

*L. stuevei* Nutt. (Jackson 1915)

*L. violacea* (L.) Pers. (Coulter 1876; McCain et al. 1990)

*L. virginica* (L.) Britton (Jackson 1915); f. deamii M. Hopkins (McCain et al. 1990)

*Lespedeza × brittonii* E.P. Bicknell (pro sp.) [*procumbens × virginica*] (McCain et al. 1990)

*Lespedeza × nuttallii* Darl. (pro sp.) [*hirta × violacea*] (McCain et al. 1990)

*Uromyces lineolatus* (Desm.) J. Schröt.

See: Uromyces lineolatus subsp. nearcticus Savile

**Uromyces lineolatus subsp. nearcticus Savile**

*Cicuta maculata* L. (Apiaceae) (Jackson 1915)

*Sium suave* Walter (=*Sium cicutifolium* Schrank) (Apiaceae)

*Schoenoplectus americanus* (Pers.) Volkart ex Schinz & R. Keller (=*Scirpus americanus* Pers.) (Cyperaceae) (Jackson 1915; Anderson and Anderson 1919)

*S. fluviatilis (Torr*.) M.T. Strong (=*Bolboschoenus fluviatilis* (Torr.) Soják); (=*Scirpus fluviatilis* (Torr.) A. Gray) (Jackson 1920; Savile 1972)

*S. tabernaemontani* (C.C. Gmel.) Palla (=*Scirpus validus* Vahl) (Jackson 1915; Cummins 1935)

*Uromyces magnatus* Arthur

See: *Uromyces acuminatus* Arthur

*Uromyces medicaginis* Pass.

See: *Uromyces striatus* J. Schröt.

***Uromyces minutus* Dietel**

*Carex lanuginosa* Michx. (Cyperaceae) (Jackson 1915)

*C. virescens* Muhl. ex Willd. (Jackson 1915)

*Uromyces orobi* (Pers.) G. Winter

See: *Uromyces viciae-fabae* (Pers.) J. Schröt.

***Uromyces peckianus* Farl.**

*Atriplex prostrata* Bouchér ex DC. (=*Atriplex hastata* L.) (Chenopodiaceae)

***Uromyces perigynius* Halst.**

Carex albicans var. albicans Willd. ex Spreng. (=*C. varia* Muhl. ex Wild) (Cyperaceae) (Jackson 1915) PUR: 12781; (=*C. artitecta* Mack.)

*C. grayi* Carey; (=*C. asa-grayi* L.H. Bailey) (Jackson 1920)

*C. intumescens* Rudge

*C. pubescens* Poir. (Underwood 1893; Arthur 1899)

*Carex* sp.

*C. tribuloides* Wahlenb. (Jackson 1915)

*Gaura biennis* L. (Onagraceae)

*Oenothera biennis* L. (Onagraceae)

*Rudbeckia laciniata* L. (Asteraceae) (Jackson 1920)

*Solidago caesia* L. (Asteraceae)

*S. canadensis* L. PUR: 12708

*S. flexicaulis* L.

*S. rugosa* Mill.

*S. serotina* Aiton

Symphyotrichum ericoides var. ericoides (L.) G.L. Nesom (=*Aster ericoides* L.) (Asteraceae)

Symphyotrichum foliaceum var. canbyi (A. Gray) G.L. Nesom (=*A. tweedyi* Rydb.)

Symphyotrichum lanceolatum subsp. lanceolatum var. lanceolatum (Willd.) G.L. Nesom (=*A. paniculatus* Lam. p.p., non)

*Uromyces phaseoli* G. Winter

See: *Uromyces appendiculatus* (Pers.) Unger

Uromyces phaseoli var. strophostylis Arthur

See: Uromyces appendiculatus var. appendiculatus (Pers.) Unger

Uromyces phaseoli var. typica Arthur, nom. inval.

See: Uromyces appendiculatus var. appendiculatus (Pers.) Unger

***Uromyces plumbarius* Peck**

*Gaura biennis* L. (Onagraceae) (Jackson 1915; Snyder 1896; Arthur 1899)

*Oenothera biennis* L. (Onagraceae) (Jackson 1915)

*Uromyces polemonii* (Peck) Barthol.

See: *Uromyces acuminatus* Arthur

*Uromyces polygoni* (Pers.) Fuckel

See: *Uromyces polygoni-avicularis* (Pers.) G.H. Otth

***Uromyces polygoni-avicularis* (Pers.) G.H. Otth**

*Polygonum aviculare* L. (Polygonaceae) (Underwood 1893; Snyder 1896; Arthur 1899; Jackson 1915; Anderson and Anderson 1919)

*P. erectum* L. (Underwood 1893; Arthur 1899; Jackson 1915)

P. punctatum Elliott var. punctatum (=*P. acre* Kunth) (Underwood 1893)

*Uromyces porosus* (Peck) H.S. Jacks.

See: *Uromyces coloradensis* Ellis & Everh.

***Uromyces proëminens* (DC.) Lév.**

*Chamaesyce humistrata* (Engelm. ex A. Gray) Small (Euphorbiaceae) (Jackson 1915); (=*Euphorbia humistrata* Engelm. ex A. Gray) (Anderson and Anderson 1919)

*C. hypericifolia* (L.) Millsp. (=*E. hypericifolia* L.) (Underwood 1893; Snyder 1896, 1898)

*C. hyssopifolia* (L.) Small

*C. maculata* (L.) Small (=*E. maculata* L.) (Underwood 1893; Snyder 1896; Jackson 1915; Anderson and Anderson 1919)

*C. nutans* (Lag.) Small (=*C. preslii* (Guss.) Arthur) (Jackson 1915); (=*E. nutans* Lag.) (Arthur 1899)

*Euphorbia corollata* L. (Euphorbiaceae) (Underwood 1893); (=*E. preslii* Guss.) (Underwood 1893; Anderson and Anderson 1919; Van Hook 1934)

*E. dentata* Michx. (Underwood 1893; Snyder 1896; Anderson and Anderson 1919); var. dentata (=*Poinsettia dentata* (Michx.) Klotzsch & Garcke) (Snyder 1896; Arthur 1899; Jackson 1915)

*E. glyptosperma* (Engelm.) Small

*E. heterophylla* L.; (=*P. heterophylla* (L.) Klotzsch & Garcke) (Jackson 1915)

*E. maculata* L.

*Euphorbia* sp. (Underwood 1893)

Host not reported (Van Hook 1910)

***Uromyces punctatus* J. Schröt.**

*Astragalus canadensis* L. (Fabaceae)

***Uromyces rhynchosporae* Ellis**

*Rhynchospora alba* (L.) Vahl (Cyperaceae) (Jackson 1915)

*R. capitellata* (Michx.) Vahl

*R. glomerata* (L.) Vahl (Jackson 1920)

***Uromyces rudbeckiae* Arthur & Holw.**

*Rudbeckia laciniata* L. (Asteraceae) (Snyder 1898; Arthur 1899; Jackson 1915; Anderson and Anderson 1919)

*Uromyces scirpi* Burrill

See: Uromyces lineolatus subsp. nearcticus Savile

***Uromyces seditiosus* F. Kern**

*Aristida longespica* Poir. (Poaceae) (Cummins and Husain 1966)

*A. oligantha* Michx. (=*A. ramosissima* Engelm.) (Jackson 1917; Jackson 1920; Cummins and Husain 1966)

*A. adscensionis* L. (McCain et al. 1990)

*Plantago aristata* Michx. (Plantaginaceae)

*P. lanceolata* L.

*P. rugelii* Decne.

***Uromyces silphii* (Syd. & P. Syd.) Arthur**

*Heliopsis helianthoides* (L.) Sweet (Asteraceae)

*Juncus anthelatus* (Wiegand) R.E. Brooks (=J. tenuis var. anthelatus Wiegand) (Juncaceae)

*J. dudleyi* Wiegand (Jackson 1915)

*Juncus* sp.

*J. tenuis* Willd. (Jackson 1915)

*Silphium integrifolium* Michx. (Asteraceae)

*S. perfoliatum* L. (Jackson 1915)

*Uromyces solidaginis-caricis* Arthur

See: *Uromyces perigynius* Halst.

***Uromyces sparganii* Cooke & Peck**

*Acorus calamus* L. (Acoraceae) (Parmelee and Savile 1954)

***Uromyces spermacoces* (Schwein.) M.A. Curtis**

*Diodia teres* Walter (Rubiaceae) (Jackson 1915; Emmons et al. 1960)

***Uromyces sporoboli* Ellis & Everh.**

*Allium canadense* L. (Amaryllidaceae)

*Allium* sp.

Sporobolus compositus var. compositus (Poir.) Merr. (=*S. asper* (P. Beauv.) Kunth) (Poaceae) (Cummins and Greene 1961)

*S. vaginiflorus* (Torr. ex A. Gray) Alph. Wood

***Uromyces striatus* J. Schröt.**

*Medicago lupulina* L. (Fabaceae) (Jackson 1915; McCain et al. 1990)

*M. sativa* L. (Jackson 1915; Pipal 1915; Ruhl et al. 1982)

*Uromyces terebinthi* (DC.) G. Winter

See: *Pileolaria brevipes* Berk. & Ravenel

*Uromyces trifolii* (Alb. & Schwein.) G. Winter, sensu Underwood (1893)

See: Uromyces trifolii-repentis var. trifolii-repentis Liro and U. trifolii-repentis var. fallens (Arthur) Cumm.

*Uromyces trifolii* (Alb. & Schwein.) G. Winter

See: Uromyces trifolii-repentis var. fallens (Arthur) Cumm.

Host not reported (Van Hook 1910)

*Uromyces trifolii* (R. Hedw.) Lév.

See: Uromyces trifolii-repentis var. trifolii-repentis

Uromyces trifolii var. fallens (Desm.) Arthur

See: Uromyces trifolii-repentis var. fallens (Desm.) Arthur

**Uromyces trifolii-repentis var. trifolii-repentis Liro**

*Trifolium hybridum* L. (Fabaceae) (Underwood 1893; Arthur 1899; Jackson 1915)

*T. medium* L. (Jackson 1915)

*T. repens* L. (Underwood 1893; Arthur 1899; Jackson 1915)

*T. campestre* Schreb. (=*T. procumbens* L.) (Anderson and Anderson 1919) [Doubtful host]

*Trifolium* sp. (Pipal 1915)

**Uromyces trifolii-repentis var. fallens (Desm.) Cumm.**

*Trifolium medium* L. (Fabaceae) (Underwood 1893; USDA 1960)

*T. pratense* L. (Underwood 1893; Snyder 1896, 1898; Arthur 1899; Jackson 1915; Anderson and Anderson 1919)

*T. repens* L. (Jackson 1915) PUR: 15471

*Trifolium* sp. (Pipal 1915)

***Uromyces triquetrus* Cooke**

*Elodes campanulata* Pursh (Clusiaceae) (Underwood 1893)

*Hypericum canadense* L. (Clusiaceae) (Underwood 1893; Arthur 1899; Jackson 1915)

*H. adpressum* W.P.C. Barton (McCain et al. 1990)

*H. cistifolium* Lam.

*H. ellipticum* Hook.

*H. kalmianum* L. (McCain and Hennen 1981)

*H. mutilum* L. (Underwood 1893; Arthur 1899; Jackson 1915)

*H. prolificum* L. (Jackson 1920; Emmons et al. 1960)

*Hypericum* sp. (Emmons et al. 1960)

*Triadenum virginicum* (L.) Raf. (Clusiaceae) (Arthur 1899; Jackson 1915)

***Uromyces valens* F. Kern**

*Carex lupulina* Muhl. ex Willd. (Cyperaceae) (Jackson 1915); (=C. lupulina Muhl. ex Willd. var. pedunculata A. Gray) (Underwood 1893)

*Carex* sp. (USDA 1960)

*C. rostrata* Stokes (Jackson 1915)

*C. utriculata* Boott (Kern 1910)

***Uromyces verruculosus* J. Schröt.**

Silene latifolia subsp. alba (Mill.) Greuter & Burdet (=*Lychnis alba* Mill.) (Caryophyllaceae) (USDA 1960)

***Uromyces viciae-fabae* (Pers.) J. Schröt.**

*Lathyrus palustris* L. (Fabaceae) (Anderson and Anderson 1919)

*Lathyrus* sp.

*L. venosus* Muhl. ex Willd. (Jackson 1915); (reported as *Vicia americana* Muhl. ex Willd.) (Snyder 1896; Arthur 1899)

***Uromyces vignae* Barcl.**

*Vigna unguiculata* (L.) Walp. (Fabaceae); (=*Vigna sinensis* (L.) Savi ex Hassk.) (Fabaceae) (Jackson 1915)

***Uropyxis amorphae* (M.A. Curtis) J. Schröt.**

*Amorpha canescens* Pursh (Fabaceae) (Underwood 1893; Arthur 1899; Jackson 1915)

## ﻿Additional information

### Author ORCIDs

Terry J. Torres-Cruz https://orcid.org/0000-0002-9193-3249

D. Rabern Simmons https://orcid.org/0000-0002-7033-225X

M. Catherine Aime https://orcid.org/0000-0001-8742-6685

### Data availability

All of the data that support the findings of this study are available in the main text or Supplementary Information.

## Supplementary materials

Supplementary material 1 Number of rust specimens deposited in the Arthur Fungarium by number of specimens per Indiana county ranked from highest to lowest Data type xls https://binary.pensoft.net/file/1405098This dataset is made available under the Open Database License (http://opendatacommons.org/licenses/odbl/1.0/). The Open Database License (ODbL) is a license agreement intended to allow users to freely share, modify, and use this Dataset while maintaining this same freedom for others, provided that the original source and author(s) are credited.Terry J. Torres-Cruz

Supplementary material 2 Metadata of specimens evaluated in the study Data type xls https://binary.pensoft.net/file/1405099This dataset is made available under the Open Database License (http://opendatacommons.org/licenses/odbl/1.0/). The Open Database License (ODbL) is a license agreement intended to allow users to freely share, modify, and use this Dataset while maintaining this same freedom for others, provided that the original source and author(s) are credited.Terry J. Torres-Cruz

Supplementary material 3 Results of searching all taxa reported for Indiana in MycoBank and IndexFungorum databases Data type xlsx https://binary.pensoft.net/file/1405100This dataset is made available under the Open Database License (http://opendatacommons.org/licenses/odbl/1.0/). The Open Database License (ODbL) is a license agreement intended to allow users to freely share, modify, and use this Dataset while maintaining this same freedom for others, provided that the original source and author(s) are credited.Terry J. Torres-Cruz

## References

[B1] Abbasi M Aime MC (2016) Two new *Puccinia* species on *Melica* (Poaceae) from USA. Mycotaxon 131(1): 247–253. 10.5248/131.247

[B2] Abbasi M Goodwin SB Scholler M (2005) Taxonomy, phylogeny, and distribution of *Puccinia graminis*, the black stem rust: New insights based on rDNA sequence data. Mycoscience 46(4): 241–247. 10.47371/mycosci.MYC46241

[B3] Abbasi M Aime MC Creswell TC Ruhl GE (2016a) First report of rust disease caused by Coleosporium apocynaceum on Amsonia ‘Blue Ice’ in Indiana. Plant Disease 100(8): 1786. 10.1094/PDIS-01-16-0019-PDN

[B4] Abbasi M Klimek JF Aime MC (2016b) First report of rust disease caused by *Puccinia liliacearum* on *Ornithogalum umbellatum* from Indiana and Maryland with notes on the spread of the rust fungus in the United States. Plant Disease 100(10): 2169. 10.1094/PDIS-04-16-0509-PDN

[B5] Aime MC Abbasi M (2018) *Puccinia modiolae* in North America: Distribution and natural host range. MycoKeys 11(39): 63–73. 10.3897/mycokeys.39.27378 PMC6160790 30271258

[B6] Aime MC McTaggart AR (2021) A higher-rank classification for rust fungi, with notes on genera. Fungal Systematics and Evolution 7(1): 21–47. 10.3114/fuse.2021.07.02 34124616 PMC8165960

[B7] Aime MC Bell CD Wilson AW (2018) Deconstructing the evolutionary complexity between rust fungi (Pucciniales) and their plant hosts. Studies in Mycology 89: 143–152. 10.1016/j.simyco.2018.02.002 29910520 PMC6002339

[B8] Anderson HW Anderson PJ (1919) The parasitic Fungi of Montgomery County, I. Proceedings of the Indiana Academy of Sciences 29: 175–222. https://journals.indianapolis.iu.edu/index.php/ias/article/view/13500

[B9] Arthur JC (1899) Indiana plant rusts, listed in accordance with latest nomenclature. Proceedings of the Indiana Academy of Sciences 8: 174–186. https://journals.indianapolis.iu.edu/index.php/ias/article/view/13487

[B10] Arthur J (1902) The Uredineæ Occurring upon Phragmites, Spartina, and Arundinaria in America. Botanical Gazette (Chicago, Ill.) 34(1): 1–20. 10.1086/328256

[B11] Arthur JC (1909) Cultures of Uredineae in 1908. Mycologia 1(6): 225–256. 10.1080/00275514.1909.12020595

[B12] Arthur JC (1918) New Species of Uredineae-X. Bulletin of the Torrey Botanical Club 45(4): 141–156. 10.2307/2479631

[B13] Arthur JC (1934) Manual of the rusts in United States and Canada. Purdue Research Foundation, Lafayette, Indiana, 438 pp.

[B14] Arthur JC Holway E (1901) Violet rusts in North America. Minnesota Botanical Studies 2: 631–641.

[B15] Bates ST Bailey EM Tobey NT (2019) Checklist of Indiana Fungi II: Microfungi. Proceedings of the Indiana Academy of Sciences 128(2): 107–130.

[B16] Batra SWT (1981) *Puccinia xanthii* forma specialis *ambrosiatrifidae*, a microcyclic rust for the biological control of giant ragweed, *Ambrosia trifida* (Compositae). Mycopathologia 73(2): 61–64. 10.1007/BF00562590

[B17] Bean DW Gladem K Rosen K Blake A Clark RE Henderson C Kaltenbach J Price J Smallwood EL Berner DK Young SL Schaeffer RN (2024) Scaling use of the rust fungus *Puccinia punctiformis* for biological control of Canada thistle (*Cirsium arvense* (L.) Scop.): First report on a U.S. statewide effort. Biological Control 192: 105481. 10.1016/j.biocontrol.2024.105481

[B18] Bennett C Aime MC Newcombe G (2011) Molecular and pathogenic variation within *Melampsora* on *Salix* in western North America reveals numerous cryptic species. Mycologia 103(5): 1004–1018. 10.3852/10-289 21558505

[B19] Böllmann J Scholler M (2006) Life cycle and life strategy features of *Puccinia glechomatis* (Uredinales) favorable for extending the natural range of distribution. Mycoscience 47(3): 152–158. 10.47371/mycosci.MYC47152

[B20] Buriticá P (1998) La Familia Phakopsoraceae en el Neotrópico - II. Generos: *Arthuria*, *Nothoravenelia*, *Uredopeltis*, *Kweilingia*, *Aplopsora* y *Pucciniostele*. Revista de la Academia Colombiana de Ciencias Exactas, Físicas y Naturales 22(80): 325–334. 10.18257/raccefyn.22(84).1998.2916

[B21] Buriticá P (1999) La familia Phakopsoraceae en el Neotrópico III, géneros: *Batistopsora* y *Phakopsora*. Revista de la Academia Colombiana de Ciencias Exactas, Físicas y Naturales 23(87): 271–305. 10.18257/raccefyn.23(87).1999.2906

[B22] Cantonwine E Nienow J Blackmore M Griffin B Bergstrom B (2019) Results of a fall and spring BioBlitz at Grassy Pond Recreational Area, Lowndes County, Georgia. Georgia Journal of Science 77: 17.

[B23] Cash EK (1952) A record of the fungi named by J.B. Ellis., Part I. Division of Mycology and Disease Survey, Bureau of Plant Industry, Soils, and Agricultural Engineering, Agricultural Research Administration, USDA. 10.5962/bhl.title.149755

[B24] Caubel J Launay M Ripoche D Gouache D Buis S Huard F Huber L Brun F Bancal MO (2017) Climate change effects on leaf rust of wheat: Implementing a couple crop-disease model in a French regional application. European Journal of Agronomy 90: 53–66. 10.1016/j.eja.2017.07.004

[B25] Cellerino GP (1999) Review of fungal diseases in Poplar. Grugliasco, Turin, 53 pp. https://openknowledge.fao.org/server/api/core/bitstreams/a247e7ff-5ddf-4e78-9bfc-2f8326c0e8d1/content

[B26] Çelekli A Zariç ÖE (2023) Utilization of herbaria in ecological studies: biodiversity and landscape monitoring. Herbarium Turcicum. 10.26650/HT.2023.1345916

[B27] Cooke WB (1967) The 1961 Indiana Foray. Mycologia 59(2): 375–381. https://www.jstor.org/stable/3756813

[B28] Cooke WB (1975) The 1970 Indiana Foray. Mycologia 67(5): 1065–1071. 10.1080/00275514.1975.12019846

[B29] Cooke WB (1986) The 1981 Indiana Foray. Mycologia 78(2): 321–323. 10.1080/00275514.1986.12025252

[B30] Coulter JM (1876) Uromyces lespedezae. Botanical Gazette (Chicago, Ill.) 1(5): 20.

[B31] Creswell TC Ruhl GE Aime MC Beckerman JL Abbasi M (2016) First report of rust disease of Bradford pear caused by *Gymnosporangium clavipes*. Plant Disease 100(4): 860. 10.1094/PDIS-10-15-1125-PDN

[B32] Cummins GB (1935) Notes on some species of the Uredinales. Mycologia 27(6): 605–614. 10.1080/00275514.1935.12017104

[B33] Cummins GB (1962) Supplement to Arthur’s Manual of the rusts in United States and Canada. Hafner, New York, 24 pp.

[B34] Cummins GB (1971) The Rust Fungi of cereals, grasses and bamboos. Springer-Verlag, New York, 570 pp. 10.1007/978-3-642-88451-1

[B35] Cummins GB (1978) Rust fungi on legumes and composites in North America. University of Arizona Press, Tucson, AZ.

[B36] Cummins G Greene H (1961) The rust fungi of *Muhlenbergia*, *Sporobolus*, and related genera. Brittonia 13(3): 271–285. 10.2307/2805343

[B37] Cummins GB Greene HC (1966) A review of the grass rust fungi that have uredial paraphyses and aecia on Berberis-Mahonia. Mycologia 58(5): 702–721. 10.1080/00275514.1966.12018364

[B38] Cummins GB Husain SM (1966) The rust fungi on the genus *Aristida*. Bulletin of the Torrey Botanical Club 93(1): 56–67. 10.2307/2483886

[B39] Cummins G Britton M Baxter J (1969) The autoecious species of *Puccinia* on North American Eupatoriae. Mycologia 61(5): 924–944. 10.1080/00275514.1969.12018816

[B40] Demers JE Liu M Hambleton S Castlebury LA (2017) Rust fungi on *Panicum*. Mycologia 109(1): 1–17. 10.1080/00275514.2016.1262656 28402789

[B41] Dudney J Willing CE Das AJ Latimer AM Nesmith JCB Battles JJ (2021) Nonlinear shifts in infections rust disease due to climate change. Nature Communications 12(1): 5102. 10.1038/s41467-021-25182-6 PMC8385051 34429405

[B42] Durkin L Jansson T Sanchez M Khomich M Ryberg M Kristiansson E Nilsson RH (2020) When mycologists describe new species, not all relevant information is provided (clearly enough). MycoKeys 72: 109–128. 10.3897/mycokeys.72.56691 32982558 PMC7498475

[B43] Emmons CW Cummins GB Cooke WB (1960) The 1958 Foray of the Mycological Society of America. Mycologia 52(5): 808–817. https://www.jstor.org/stable/3755883

[B44] Farr DF Rossman AY (2018) Fungal Databases, U.S. National Fungus Collections, ARS, USDA. https://nt.ars-grin.gov/fungaldatabases/ [Accessed October 2024]

[B45] Furnas A (1874) Rust in blackberries. Transactions of the Indiana Horticultural Society 14: 74.

[B46] Greve M Lykke AM Fagg CW Gereau RE Lewis GP Marchant R Marshall AR Ndayishimiye J Bogaert J Svenning J-C (2016) Realising the potential of herbarium records for conservation biology. South African Journal of Botany 105: 317–323. 10.1016/j.sajb.2016.03.017

[B47] Hanlin RT Foudin LL Berisford Y Glover SU Jones JP Huang LH (1978) Plant disease index for maize in the US part 1: Host index. Agricultural Experimental Stations, University of Georgia. Research Reports (Montgomery) 277: 1–62.

[B48] Heberling JM Prather LA Tonsor SJ (2019) The changing uses of herbarium data in an era of global change: An overview using automated content analysis. Bioscience 69(10): 812–822. 10.1093/biosci/biz094

[B49] Hennen JF (1965) The species of *Uromyces* parasitic on the grass tribe Andropogoneae. Mycologia 57(1): 104–113. 10.1080/00275514.1965.12018196

[B50] Hernández JR Palm ME Castlebury LA (2002) *Puccinia hemerocallidis*, cause of daylily rust, a newly introduced disease in the Americas. Plant Disease 86(11): 1194–1198. 10.1094/PDIS.2002.86.11.1194 30818466

[B51] Hiratsuka N (1958) Revision of Taxonomy of the Pucciniastreae: With Special Reference to Species of the Japanese Archipelago. KASAI Publishing and Printing Co., Tokyo, 167 pp.

[B52] IPBES (2016) The assessment report of the intergovernmental science-policy platform on biodiversity and ecosystem services on pollinators, pollination and food production. In: Potts SG, Imperatriz-Fonseca VL, Ngo HT (Eds) Secretariat of the Intergovernmental Science-Policy Platform on Biodiversity and Ecosystem Services. IPBES Secretariat, Bonn, Germany.

[B53] IUCN (2020) The IUCN red list of threatened species. Version 2020–1.

[B54] Jackson HS (1915) The Uredinales of Indiana. Proceedings of the Indiana Academy of Sciences 25: 429–476. https://journals.indianapolis.iu.edu/index.php/ias/article/view/14504

[B55] Jackson HS (1917) The Uredinales of Indiana II. Proceedings of the Indiana Academy of Sciences 27: 133–138. https://journals.indianapolis.iu.edu/index.php/ias/article/view/13289

[B56] Jackson HS (1920) The Uredinales of Indiana III. Proceedings of the Indiana Academy of Sciences 30: 165–182. https://journals.indianapolis.iu.edu/index.php/ias/article/view/13580

[B57] Jones CA Daehler CC (2018) Herbarium specimens can reveal impacts of climate change on plant phenology; a review of methods and applications. PeerJ 6: e4576. 10.7717/peerj.4576 PMC5888139 29632745

[B58] Kaishian P Layug CRK Anderson M Berg DR Aime MC (2024) Rust HUBB: DNA barcode-based identification of Pucciniales. IMA Fungus 15(1): 3. 10.1186/s43008-023-00132-7 38402196 PMC10894486

[B59] Kaishian P Creswell T Bonkowski J Aime MC (2023) First report of smoketree rust caused by *Pileolaria cotini-coggygriae* in the Midwestern United States. Plant Health Progress 24(2): 226–227. 10.1094/PHP-08-22-0080-BR

[B60] Kenaley SC Quan M Aime MC Bergstrom GC (2018) New insight into the species diversity and life cycles of rust fungi (Pucciniales) affecting bioenergy switchgrass (*Panicum virgatum*) in the Eastern and Central United States. Mycological Progress 17(11): 1251–1267. 10.1007/s11557-018-1434-1

[B61] Kern FD (1910) Two New Species of Uromyces on Carex. Rhodora 12(138): 124–127. https://biostor.org/reference/179036

[B62] Kern FD (1919) North American rusts on *Cyperus* and *Eleocharis*. Mycologia 11(3): 134–147. 10.1080/00275514.1919.12016788

[B63] Lacher Jr TE Mallon D Kennerley RJ Relton C Young RP (2022) Tools and metrics for species prioritization for conservation planning and action: Case studies for antelopes and small mammals. Diversity 14(9): 704. 10.3390/d14090704

[B64] Lang PLM Willems FM Scheepens JF Burbano HA Bossdorf O (2019) Using herbaria to study global environmental change. The New Phytologist 221(1): 110–122. 10.1111/nph.15401 30160314 PMC6585664

[B65] Laundon GF (1963) Rust fungi 1: On Acanthaceae. Mycological Papers 89: 1–89.

[B66] Lee HJ (1929) New parasitic Fungi of Montgomery County. Proceedings of the Indiana Academy of Sciences 39: 135–136. https://journals.indianapolis.iu.edu/index.php/ias/article/view/5519

[B67] Lopez-Franco RM Hennen JF (1990) The genus *Tranzschelia* (Uredinales) in the Americas. Systematic Botany 15(4): 560–591. 10.2307/2419155

[B68] Lowman M Ruppert N Mohd Nor SA (2018) Further advancing the expert bioblitz for the rainforest conservation toolkit. Conservation Science and Practice 1(1): e2. 10.1111/csp2.2

[B69] Maharani N Kusrini MD Hamidy A (2022) Increasing herpetofauna data through citizen science in Indonesia. IOP Conference Series. Earth and Environmental Science 950(1): 012063. 10.1088/1755-1315/950/1/012063

[B70] Mains EB (1933) Host specialization in the leaf rust of grasses *Puccinia rubigo-vera*. Papers of the Michigan Academy of Science, Arts and Letters 17: 289–394.

[B71] McCain JW (1984) A preliminary review and multiple-entry key to the rust fungi on Cyperaceae and Juncaceae in Indiana. Proceedings of the Indiana Academy of Sciences 94: 447–454. https://journals.indianapolis.iu.edu/index.php/ias/article/view/8380

[B72] McCain JW Hennen JF (1981) Notes on biogeography and new records of rust Fungi in the Great Lakes Region. Proceedings of the Indiana Academy of Sciences 91: 504–514. https://journals.indianapolis.iu.edu/index.php/ias/article/view/7773

[B73] McCain JW Hennen JF (1982) The Arthur Herbarium centennial: 100 years of uredinology in Indiana and the Great Lakes Region. Proceedings of the Indiana Academy of Sciences 92: 389–395. https://journals.indianapolis.iu.edu/index.php/ias/article/view/8115

[B74] McCain JW Hennen JF Ono Y (1990) New host species and state distribution records for North American rust fungi (Uredinales). Mycotaxon 39: 281–300.

[B75] McTaggart AR Aime MC (2018) The species of *Coleosporium* (Pucciniales) on *Solidago* in North America. Fungal Biology 122(8): 800–809. 10.1016/j.funbio.2018.04.007 30007430

[B76] Menchetti M Cianferoni F Mazza G Dal Cin M Barbato D Benocci A Cervo R Dapporto L Picchi MS Vanni L Cabrini R Mori E (2021) Checklist of macro-invertebrates of the special conservation area “Poggi di Prata” (Grosseto, Central Italy) through a citizen-science and expert-based approach. Redia (Firenze) 104: 63–68. 10.19263/REDIA-104.21.07

[B77] Microsoft Corporation (2024) Microsoft Excel.

[B78] Miller IF Jiranek J Brownell M Coffrey S Gray B Stahl M Metcalf JE (2022) Predicting the effects of climate change on the cross-scale epidemiological dynamics of a fungal plant pathogen. Scientific Reports 12(1): 14823. 10.1038/s41598-022-18851-z 36050344 PMC9437057

[B79] Molano-Flores B Johnson SA Marcum PB Feist MA (2023) Utilizing herbarium specimens to assist with the listing of rare plants. Frontiers in Conservation Science 4: 1–15. 10.3389/fcosc.2023.1144593

[B80] Monoson HL (1975) The species of Triphragmium, Nyssopsora, and Triphragmiopsis. Mycopathologia 52(2): 115–131. 10.1007/BF02128054

[B81] MyCoPortal (2025) MyCoPortal. http://www.mycoportal.org/portal/index.php [Accessed on April 2025]

[B82] Newcombe G Striling B McDonals S Bradshaw HD (2000) *Melampsora × columbiana*, a natural hybrid of *M. medusae* and *M. occidentalis*. Mycological Research 104(3): 261–274. 10.1017/S0953756299001665

[B83] Nicolai A Guernion M Guillocheau S Hoeffner K Le Gouar P Ménard N Piscart C Vallet D Hervé ME Benezeth E Chedanne H Blémus J Vernon P Cylly D Hotte H Loïs G Mai B Perez G Ouisse T Monard C Wiegand C Caudal J-P Butet A Dahirel M Barbe L Balbi M Briand V Bormans M Charrier M Bouger G Jung V Lann CL Pannard A Petillon J Rantier Y Marguerie D Tougeron K Devogel P Dugravot S Dubos T Garrin M Carnet M Gouraud C Chambet A Esnault J Poupelin M Welk E Bütof A Dubois GF Humbert G Marie-Réau O Norvez O Richard G Froger B Rochais C Potthoff M Ayati K Bellido A Rissel A Santonja M Farcy J-O Collias E Sene L Cluzeau D Supper R (2020) Transdisciplinary Bioblitz: Rapid biotic and abiotic inventory allows studying environmental changes over 60 years at the biological field station of Paimpont (Brittany, France) and opens new interdisciplinary research opportunities. Biodiversity Data Journal 8: e50451. 10.3897/BDJ.8.e50451 PMC7125239 32269479

[B84] Otálora MAG Berndt R (2018) A taxonomic revision of the genus *Puccinia* on Lycieae, a tribe of Solanaceae. Mycologia 110(4): 692–709. 10.1080/00275514.2018.1478538 30067460

[B85] Park DA Lyra GM Ellison AM Barbosa Maruyama RK Reis Torquato D Asprino RC Cook BI Davis CC (2022) Herbarium records provide reliable phenology estimates in the understudied tropics. Journal of Ecology 111(2): 327–337. 10.1111/1365-2745.14047

[B86] Parker SS Pauly GB Moore J Fraga NS Knapp JJ Principe Z Brown BV Randall JM Cohen BS Wake TA (2018) Adapting the bioblitz to meet conservation needs. Conservation Biology 32(5): 1007–1019. 10.1111/cobi.13103 29493001

[B87] Parmelee JA Savile DBO (1954) Life history and relationship of the rusts of *Sparganium* and *Acorus*. Mycologia 46(6): 823–836. 10.1080/00275514.1954.12024419

[B88] Parmelee JA Savile DBO (1981) Autoecious species of *Puccinia* on Cichorieae in North America. Canadian Journal of Botany 59(6): 1078–1101. 10.1139/b81-147

[B89] Pearman PB Broennimann O Aavik T Albayrak T Alves PC Aravanopoulos FA Bertola LD Biedrzycka A Buzan E Cubric-Curik V Djan M Fedorca A Fuentes-Pardo AP Fussi B Godoy JA Gugerli F Hoban S Holderegger R Hvilsom C Lacolina L Kalamujic B Klinga P Konopiński MK Kopatz A Laikre L Lopes-Fernandes M McMahon BJ Mergeay J Neophytou C Pálsson S Paz-Vinas I Posledovich D Primmer CR Raymaekers JAM Rinkevich B Rolečková B Runġis D Schuerz L Segelbacher G Kavčič K Stefanovic M Thurfjell H Träger S Tsvetkov IN Velickovic N Vergeer P Vernesi C Vilà C Westergren M Zachos FE Guisan A Bruford M (2024) Monitoring of species’ genetic diversity in Europe varies greatly and overlooks potential climate change impacts. Nature Ecology & Evolution 8(2): 267–281. 10.1038/s41559-023-02260-0 38225425 PMC10857941

[B90] Pierson T Stratmann T White EC Clause A Carter C Herr M Jenkins A Vogel H Knoerr M Folt B (2014) New county records of amphibians and reptiles resulting from a bioblitz competition in North-Central Georgia, USA. Herpetological Review 45: 296–297.

[B91] Pipal FJ (1915) A list of plant diseases of economic importance in Indiana with bibliography. Proceedings of the Indiana Academy of Sciences 25: 379–414. https://journals.indianapolis.iu.edu/index.php/ias/article/view/14498

[B92] Ramachar P Cummins GB (1965) The species of *Puccinia* on the Paniceae. Mycopathologia 25(1–2): 7–60. 10.1007/BF02049611

[B93] Reyserhove L Desmet P Oldoni D Adriaens T Strubbe D Davis AJS Vanderhoeven S Verloove F Groom Q (2020) A checklist recipe: Making species data open and FAIR. Database (Oxford) 2020: baaa084. 10.1093/database/baaa084 PMC7661093 33181821

[B94] Ruhl GE McCain JW (1986) First report of rust caused by *Frommeella duchesneae* on false strawberry in Indiana. Plant Disease 70(5): 475. 10.1094/PD-70-475c

[B95] Ruhl GE Scott DH Pecknold PC (1980) A compilation of plant diseases and disorders in Indiana. Proceedings of the Indiana Academy of Sciences 90: 107–124. https://journals.indianapolis.iu.edu/index.php/ias/article/view/5835

[B96] Ruhl GE Latin RX Pecknold PC Scott DH (1981) A compilation of plant diseases and disorders in Indiana. Proceedings of the Indiana Academy of Sciences 91: 120–139. https://journals.indianapolis.iu.edu/index.php/ias/article/view/7435

[B97] Ruhl GE Latin RX Pecknold PC Scott DH (1982) Compilation of plant diseases and disorders in Indiana. Proceedings of the Indiana Academy of Sciences 92: 97–118. https://journals.indianapolis.iu.edu/index.php/ias/article/view/8035

[B98] Ruhl GE Latin RX Pecknold PC Scott DH (1983) A compilation of plant diseases and disorders in Indiana. Proceedings of the Indiana Academy of Sciences 93: 103–120. https://journals.indianapolis.iu.edu/index.php/ias/article/view/7515

[B99] Savile DBO (1970) Autoecious *Puccinia* species attacking Cardueae in North America. Canadian Journal of Botany 48(9): 1553–1566. 10.1139/b70-232

[B100] Savile DBO (1971) Coevolution of the Rust Fungi and Their Hosts. The Quarterly Review of Biology 46(3): 211–218. 10.1086/406895

[B101] Savile DBO (1972) Some rusts of *Scirpus* and allied genera. Canadian Journal of Botany 50(12): 2579–2596. 10.1139/b72-331

[B102] Savile DBO (1976) *Phragmidium ivesiae* and its allies in North America. Canadian Journal of Botany 54(14): 1690–1696. 10.1139/b76-182

[B103] Schall RA McCain JW Hennen JF (1983) Distribution of *Puccinia polysora* in Indiana and the absence of cool weather form as determined by comparison with *P. sorghi*. Plant Disease 67(7): 767–770. 10.1094/PD-67-767

[B104] Schoknecht JD (1981) Contribution to the Flora of Indiana: Fungi of west-central Indiana on the occasion of the sixth annual A. H. Smith Great Lakes foray. Proceedings of the Indiana Academy of Sciences 91: 140–143. https://journals.indianapolis.iu.edu/index.php/ias/article/view/7436

[B105] Scholler M (2000) Rust on ground-ivy found for the first time in North America. Plant Disease 84(3): 371. 10.1094/PDIS.2000.84.3.371D 30841261

[B106] Seebens H Blackburn TM Dyer EE Genovesi P Hulme PE Jeschke JM Pagad S Pyšek P Winter M Arianoutsou M Bacher S Blasius B Brundu G Capinha C Celesti-Grapow L Dawson W Dullinger S Fuentes N Jäger H Kartesz J Kenis M Kreft H Kühn I Lenzner B Liebhold A Mosena A Moser D Nishino M Pearman D Pergl J Rabitsch W Rojas-Sandoval J Roques A Rorke S Rossinelli S Roy HE Scalera R Schindler S Štajerová K Tokarska-Guzik B van Kleunen M Walker K Weigelt P Yamanaka T Essl F (2017) No saturation in the accumulation of alien species worldwide. Nature Communications 8(1): 14435. 10.1038/ncomms14435 PMC5316856 28198420

[B107] Snyder L (1896) The Uredineae of Tippecanoe County, Ind. Proceedings of the Indiana Academy of Sciences 6: 216–224. https://journals.indianapolis.iu.edu/index.php/ias/article/view/13130

[B108] Snyder L (1898) The Uredineae of Madison and Noble Counties with additional specimens from Tippecanoe County. Proceedings of the Indiana Academy of Sciences 8: 186–189. https://journals.indianapolis.iu.edu/index.php/ias/article/view/13488

[B109] Tanner RA Pollard KM Varia S Evans HC Ellison CA (2015) First release of a fungal classical biocontrol agent against an invasive alien weed in Europe: Biology of the rust, Puccinia komarovii var. glanduliferae. Plant Pathology 64(5): 1130–1139. 10.1111/ppa.12352

[B110] Ullstrup AJ Lavoilette FA (1959) Diseases of corn and of sorghum species in Indiana in 1958. The Plant Disease Reporter 43: 334–336.

[B111] Underwood LM (1893) List of cryptogams at present known to inhabit the state of Indiana. Proceedings of the Indiana Academy of Sciences 3: 30–67. https://journals.indianapolis.iu.edu/index.php/ias/article/view/12643

[B112] Underwood LM (1896) Additions to the published lists of Indiana cryptogams. Proceedings of the Indiana Academy of Sciences 6: 171–172. https://journals.indianapolis.iu.edu/index.php/ias/article/view/13120

[B113] United States Department of Agriculture (1960) Index of Plant Diseases in the United States. US Government Printing Office, Washington, DC. https://www.govinfo.gov/content/pkg/GOVPUB-A-PURL-gpo20004/pdf/GOVPUB-A-PURL-gpo20004.pdf

[B114] Urban Z (1971) The autoecious species of Puccinia on Vernonieae in North America. Acta Universitatis Carolinae. Biologica: 1–84.

[B115] Van Hook JM (1910) Indiana Fungi. Proceedings of the Indiana Academy of Sciences 20: 205–212. https://journals.indianapolis.iu.edu/index.php/ias/article/view/14883

[B116] Van Hook JM (1912) Indiana Fungi— III. Proceedings of the Indiana Academy of Sciences 22: 99–101. https://journals.indianapolis.iu.edu/index.php/ias/article/view/14099

[B117] Van Hook JM (1921) Indiana Fungi— VI. Proceedings of the Indiana Academy of Sciences 31: 143–148. https://journals.indianapolis.iu.edu/index.php/ias/article/view/13884

[B118] Van Hook JM (1923) Indiana Fungi— VII. Proceedings of the Indiana Academy of Sciences 33: 233–238. https://journals.indianapolis.iu.edu/index.php/ias/article/view/13999

[B119] Van Hook JM (1924) Indiana Fungi— VIII. Proceedings of the Indiana Academy of Sciences 34: 317–320. https://journals.indianapolis.iu.edu/index.php/ias/article/view/3901

[B120] Van Hook JM (1925) Indiana Fungi— IX. Proceedings of the Indiana Academy of Sciences 35: 233–236. https://journals.indianapolis.iu.edu/index.php/ias/article/view/4196

[B121] Van Hook JM (1927) Indiana Fungi— X. Proceedings of the Indiana Academy of Sciences 37: 365–372. https://journals.indianapolis.iu.edu/index.php/ias/article/view/4572

[B122] Van Hook JM (1928) Indiana Fungi— XI. Proceedings of the Indiana Academy of Sciences 38: 127–130. https://journals.indianapolis.iu.edu/index.php/ias/article/view/4976

[B123] Van Hook JM (1929) Indiana Fungi, XII. Proceedings of the Indiana Academy of Sciences 39: 75–84. https://journals.indianapolis.iu.edu/index.php/ias/article/view/5182

[B124] Van Hook JM (1934) Indiana Fungi, XIII. Proceedings of the Indiana Academy of Sciences 44: 55–64. https://journals.indianapolis.iu.edu/index.php/ias/article/view/4654

[B125] Vargas-Gaete R Doussoulin H Smith-Ramírez C Bravo S Salas-Eljatib C Andrade N Trávníček B (2019) Evaluation of rust pathogenicity (Phragmidium violaceum) as a biological control agent for the invasive plant *Rubus ulmifolius* on Robinson Crusoe Island, Chile. Australasian Plant Pathology 48(3): 201–208. 10.1007/s13313-019-0615-y

[B126] Walker JT Earhart EF (1962) Medlar is host for *Gymnosporangium clavipes*. The Plant Disease Reporter 46: 293.

[B127] Williams TM Schlichting CD Holsinger KE (2021) Herbarium records demonstrate changes in flowering phenology associated with climate change over the past century within the Cape Floristic Region, South Africa. Climate Change Ecology 1: 100006. 10.1016/j.ecochg.2021.100006

[B128] Wilson A Beckerman J Aime M (2014) First report of the White Pine Blister Rust Fungus, *Cronartium ribicola*, on *Ribes odoratum* in Indiana. Plant Disease 98(2): 277. 10.1094/PDIS-04-13-0442-PDN 30708746

